# Supplementary figures and images for: IntAct: A nondisruptive internal tagging strategy to study the organization and function of actin isoforms
Source: PLoS Biol. 2024 Mar 11;22(3):e3002551. doi: 10.1371/journal.pbio.3002551 (PMC10957077; doi:10.1371/journal.pbio.3002551)

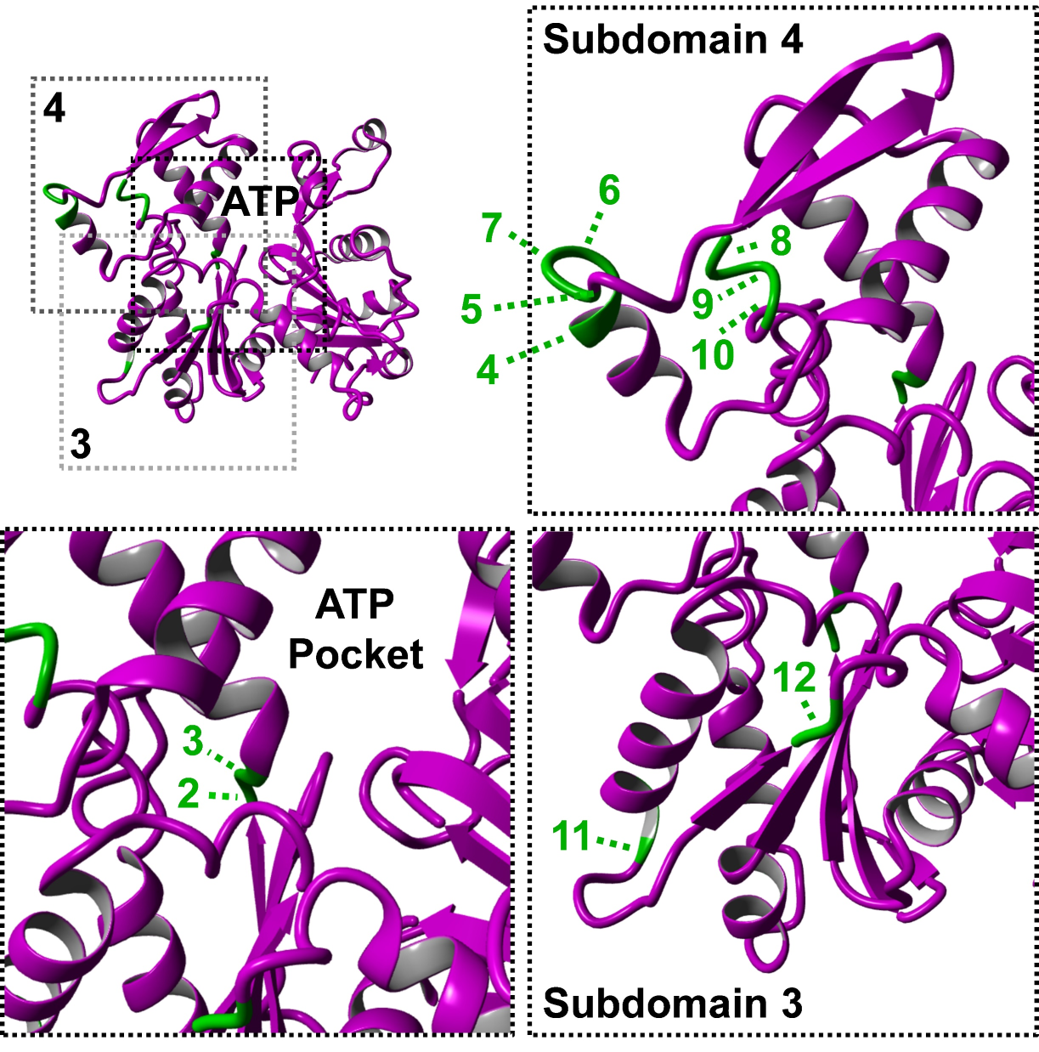

Supplement: S1 Fig — Crystal structure of uncomplexed globular actin (magenta ribbon, PBD accession number: 1J6Z32) indicating subdomain 3, subdomain 4, and the ATP pocket. Zooms show each domain and ATP pocket and their associated position for each distinct residue pair. (TIF) [file pbio.3002551.s001.tif]

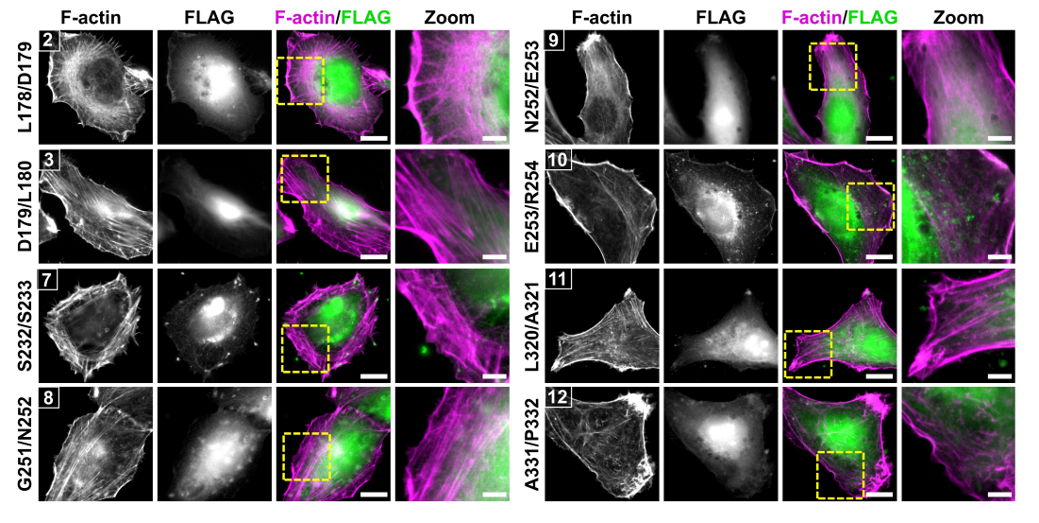

Supplement: S2 Fig — Representative widefield immunofluorescence images of F-actin (magenta) and FLAG (green) in HT1080 cells that overexpress the tagged β-actin variants. Shown are the 8 internally tagged variants that are not depicted in Fig 1B. Scale bar: 15 μm. Scale bar zoom: 5 μm. (TIF) [file pbio.3002551.s002.tif]

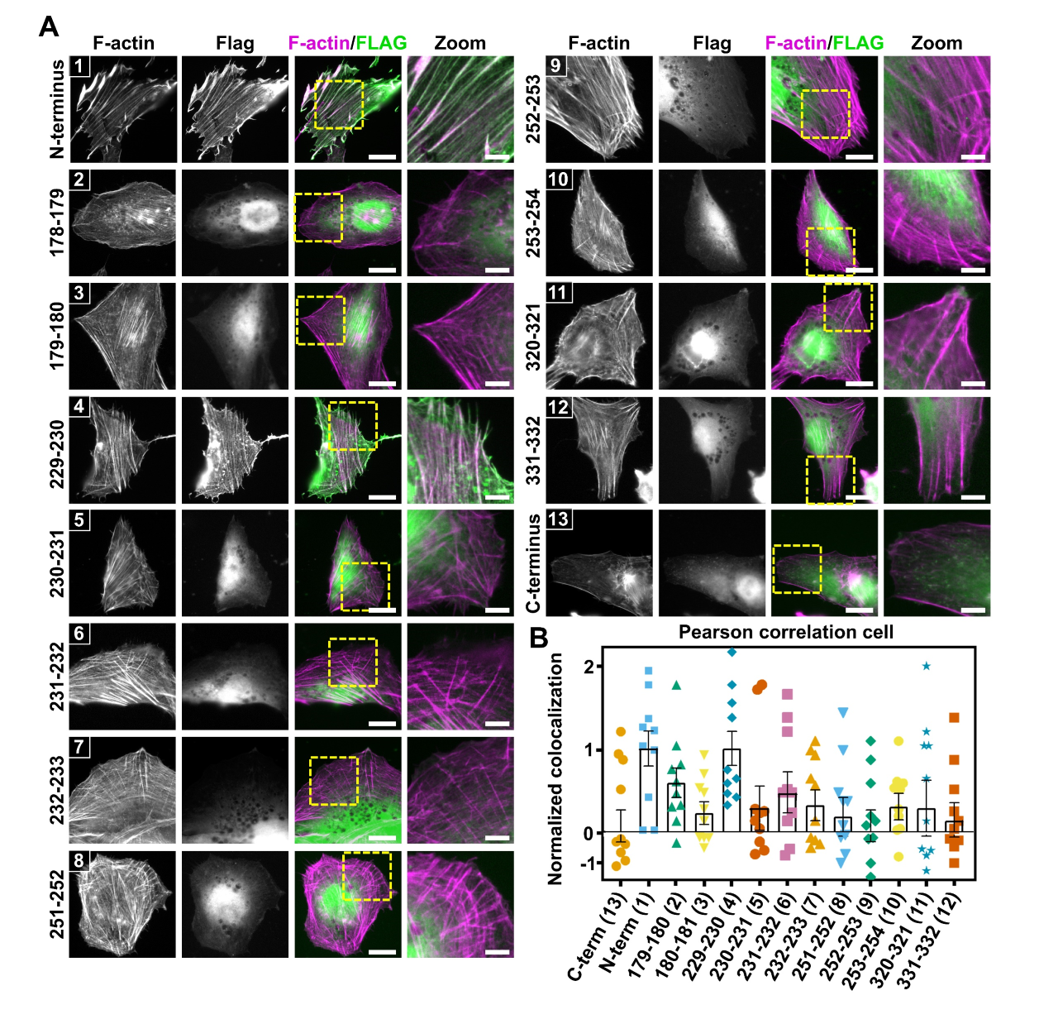

Supplement: S3 Fig — (A) Representative widefield immunofluorescence images of F-actin (magenta) and FLAG (green) in RPE1 cells that overexpress the tagged β-actin variants. Shown are 11 internally tagged variants and the N- and C-terminally tagged β-actin. Scale bar: 15 μm. Scale bar zoom: 5 μm. (B) Colocalization analysis of the microscopy results in A showing the normalized Pearson’s correlation coefficient for each of the actin variants. Individual data points indicate single cells and in total, at least 10 cells from 2 independent experiments were included in the analysis. Bars represent the mean value, and error bars represent standard error of mean (SEM). The numerical data underlying this figure can be found in S1 Data. (TIF) [file pbio.3002551.s003.tif]

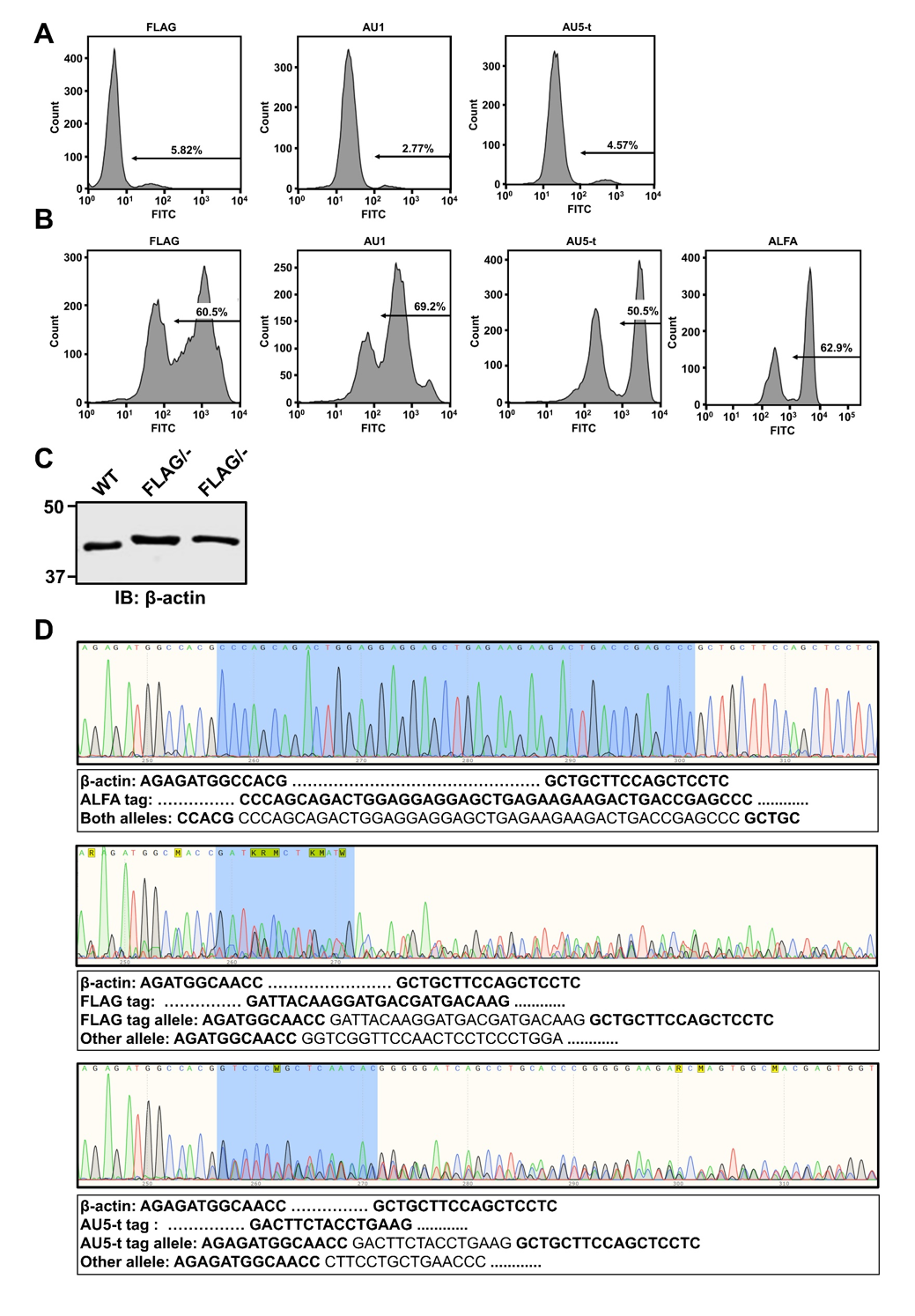

Supplement: S4 Fig — (A) Flow cytometry data of cells having a CRISPR/Cas9-mediated knock-in of FLAG, AU1, or AU5. Pool of cells was stained for their appropriate tag. (B) Flow cytometry data of cells having a CRISPR/Cas9-mediated knock-in of FLAG, AU1, AU5, or ALFA after selection with Ouabain. Pool of cells was stained for their appropriate tag. (C) Representative western blot of β-actin in parental HT1080 (WT) and 2 independent hemizygous FLAG-β-actin HT1080 clones (FLAG/-). (D) Sanger sequencing result of homozygous ALFA-β-actin, hemizygous FLAG-β-actin, and hemizygous AU5-β-actin HT1080 cells. Highlighted in blue is the ALFA, FLAG, or AU5-t sequence at position T229/A230 in β-actin. Alignment of β-actin sequence, the tag sequence in 1 or 2 alleles and the possible disrupted allele sequence. (TIF) [file pbio.3002551.s004.tif]

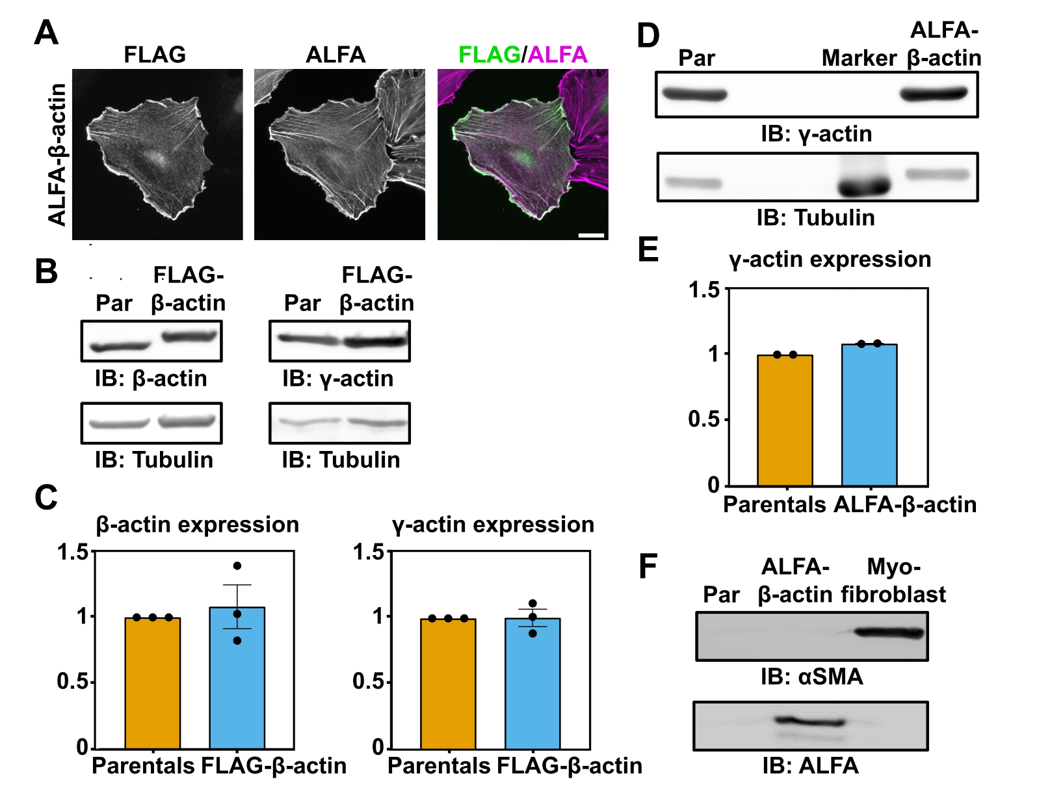

Supplement: S5 Fig — (A) Representative widefield images from cells that have a CRISPR/Cas9-mediated knock-in of FLAG in γ-actin in ALFA-β-actin cells. Cells were labeled for ALFA (magenta) and FLAG (green) staining, respectively, for β-actin and γ-actin. Scale bar: 15 μm. (B) Representative western blot showing β-actin and γ-actin expression in parental HT1080 (Par) and hemizygous FLAG-β-actin HT1080 cells. Tubulin was used as a loading control. (C) Quantification of B showing β-actin and γ-actin expression in parental HT1080 and hemizygous FLAG-β-actin HT1080 cells normalized to tubulin. Individual data points represent 3 independent western blots. Bars represent the mean value and error bars represent standard error of mean (SEM). (D) Representative western blot showing γ-actin expression in parental HT1080 (Par) and homozygous ALFA-β-actin HT1080 cells. Tubulin was used as a loading control. (E) Quantification of the γ-actin expression in ALFA-β-actin and parental HT1080 cells normalized to tubulin. Individual data points represent 2 independent western blots. Bars represent the mean value and error bars represent standard deviation. (F) Representative western blot showing αSMA and ALFA tag expression in parental HT1080 (Par), homozygous ALFA-β-actin HT1080 cells, and myofibroblast (positive control for αSMA). The numerical data underlying this figure can be found in S1 Data. (TIF) [file pbio.3002551.s005.tif]

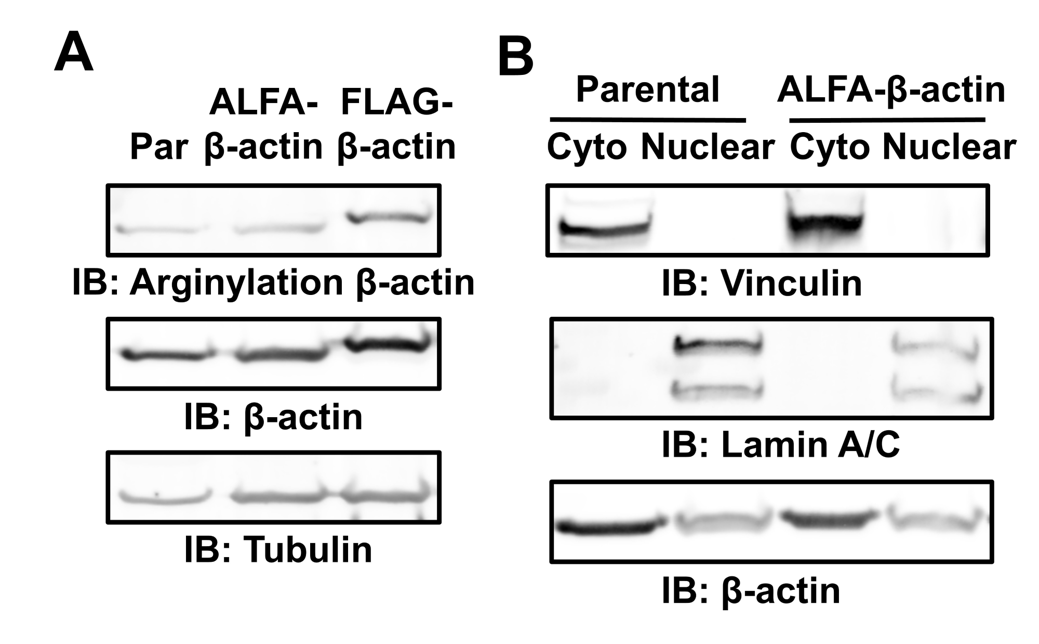

Supplement: S6 Fig — (A) Representative western blot showing arginylated β-actin and β-actin expression in parental HT1080 (Par), homozygous ALFA-β-actin HT1080 cells, and hemizygous FLAG-β-actin HT1080 cells. Tubulin was used as a loading control. (B) Representative western blot of nuclear fractionation assay showing vinculin (cytosol marker), Lamin A/C (nuclear marker), and β-actin expression in parental HT1080 and homozygous ALFA-β-actin HT1080 cells. (TIF) [file pbio.3002551.s006.tif]

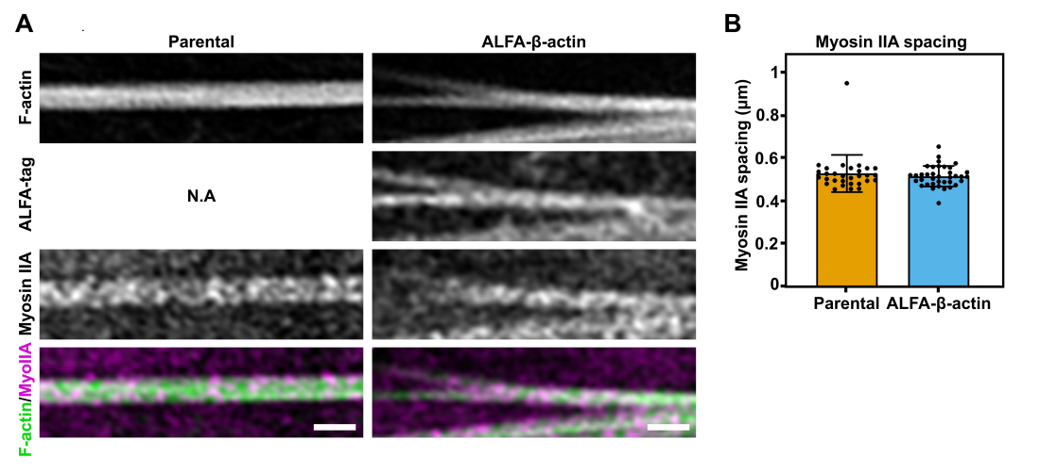

Supplement: S7 Fig — (A) Representative Airyscan images of stress fibers from parental and ALFA-β-actin cells stained against F-actin, ALFA tag, and Myosin IIA. Scale bar: 1 μm. (B) Quantification of the Myosin IIA spacing on stress fibers in A. Bars represent the mean value and error bars represent the standard deviation. In total, the average myosin spacing of at least 30 stress fibers from 15 different cells are included in the analysis. The numerical data underlying this figure can be found in S1 Data. (TIF) [file pbio.3002551.s007.tif]

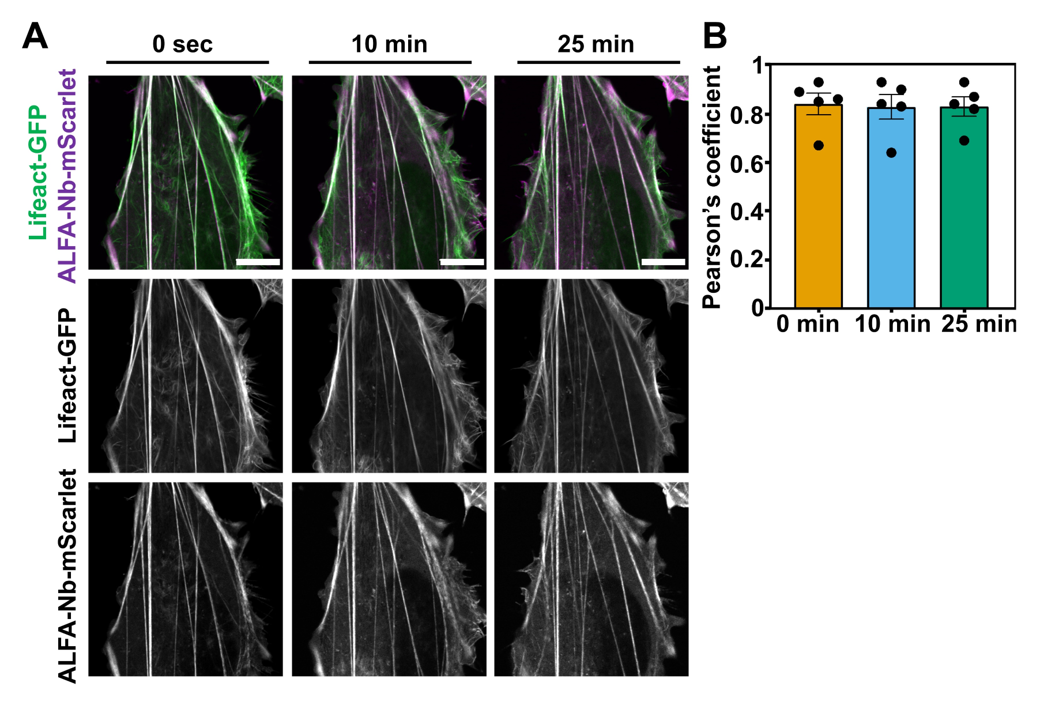

Supplement: S8 Fig — (A) Representative Airyscan images of HT1080 cells expressing LifeAct-GFP (green) and ALFA-tag Nb-mScarlet (magenta) after 0 min, 10 min, and 25 min. Full movie is available as S1 Movie. Scale bar: 10 μm. (B) Colocalization analysis of the microscopy results in A showing the Pearson’s coefficient for each time point. Individual data points indicate single cells and in total, 5 different movies were included in the analysis. Bars represent the mean value and error bars represent standard error of mean (SEM). The numerical data underlying this figure can be found in S1 Data. (TIF) [file pbio.3002551.s008.tif]

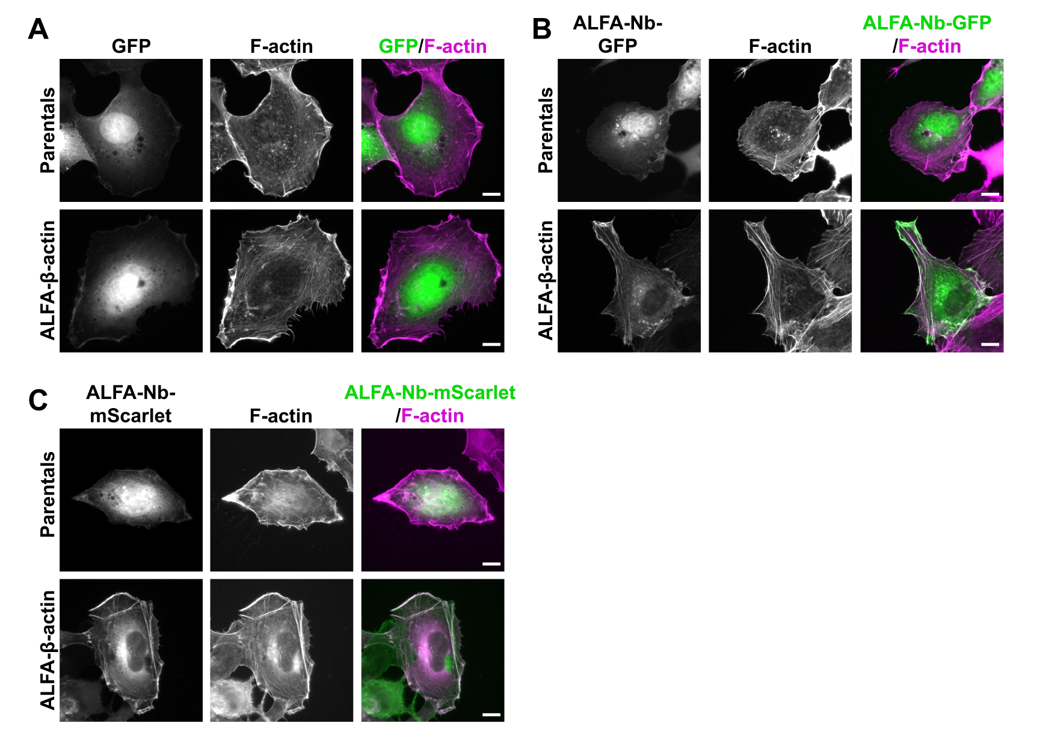

Supplement: S9 Fig — (A) Representative widefield images of HT1080 parental cells and ALFA-β-actin cells expressing GFP (green) and stained for F-actin (magenta). Scale bar: 10 μm. (B) Representative widefield images of HT1080 parental cells and ALFA-β-actin cells expressing ALFA-Nb-GFP (green) and stained for F-actin (magenta). Scale bar: 10 μm. (C) Representative widefield images of HT1080 parental cells and ALFA-β-actin cells expressing ALFA-Nb-mScarlet (green) and stained for F-actin (magenta). Scale bar: 10 μm. The numerical data underlying this figure can be found in S1 Data. (TIF) [file pbio.3002551.s009.tif]

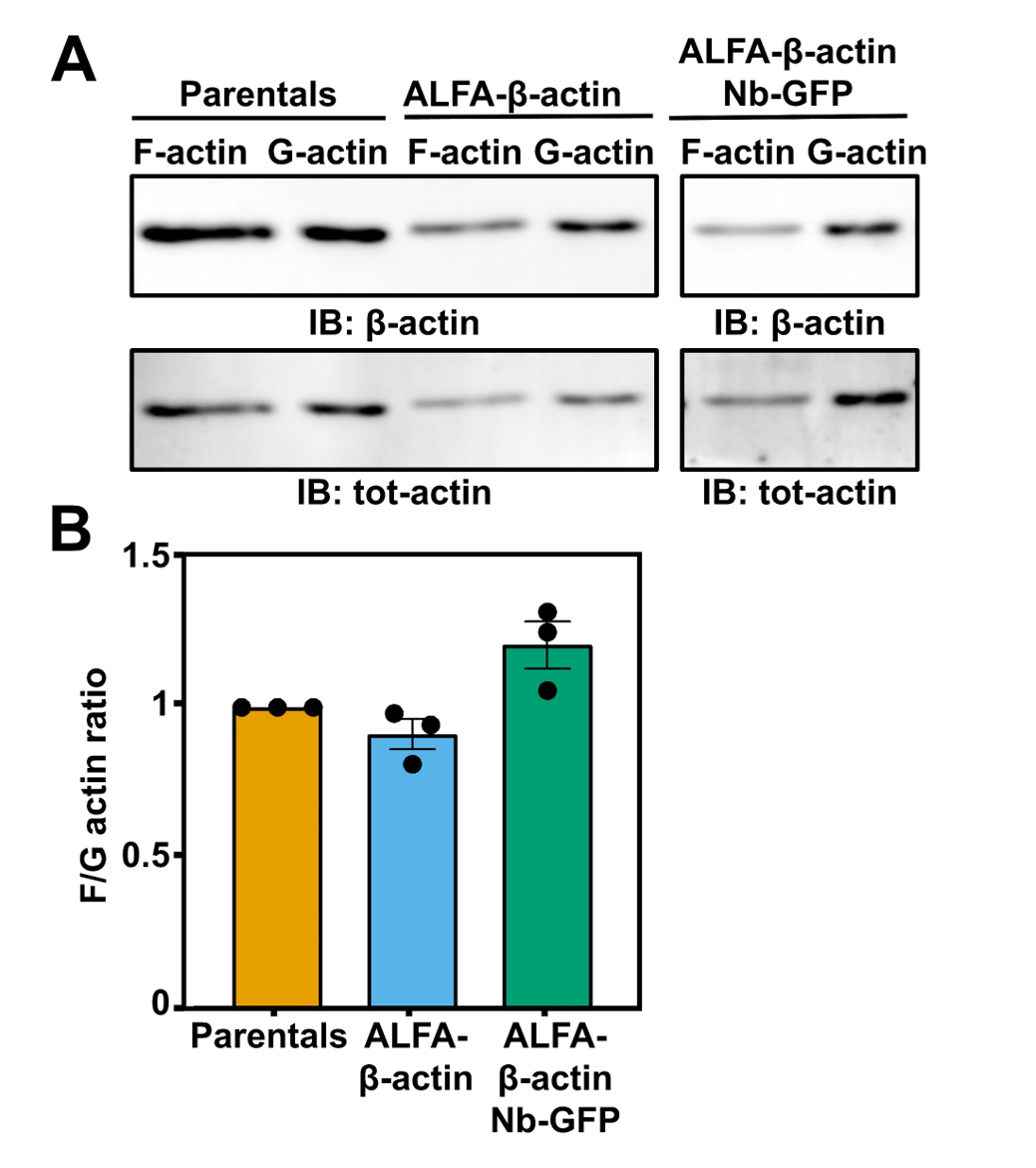

Supplement: S10 Fig — (A) Representative western blot of F-actin and G-actin fraction in parental HT1080 and homozygous ALFA-β-actin HT1080 cells with and without ALFA-Nb-GFP. Total actin was used as a loading control. (B) Quantification of the F/G-actin ratio for β-actin from the western blots shown in A. Ratios were normalized against HT1080 parentals. Individual data points represent 3 independent western blots. Bars represent the mean value and error bars represent standard error of mean (SEM). The numerical data underlying this figure can be found in S1 Data. (TIF) [file pbio.3002551.s010.tif]

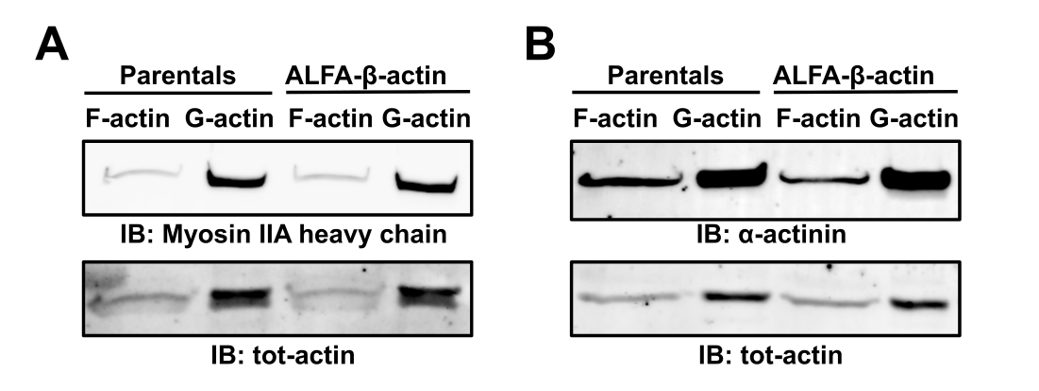

Supplement: S11 Fig — (A) Representative western blot of myosin IIA in the F-actin and G-actin fraction in parental HT1080 and homozygous ALFA-β-actin HT1080. Total actin (tot-actin) was used as a loading control. (B) Representative western blot of α-actinin in the F-actin and G-actin fraction in parental HT1080 and homozygous ALFA-β-actin HT1080 cells. Total actin was used as a loading control. (TIF) [file pbio.3002551.s011.tif]

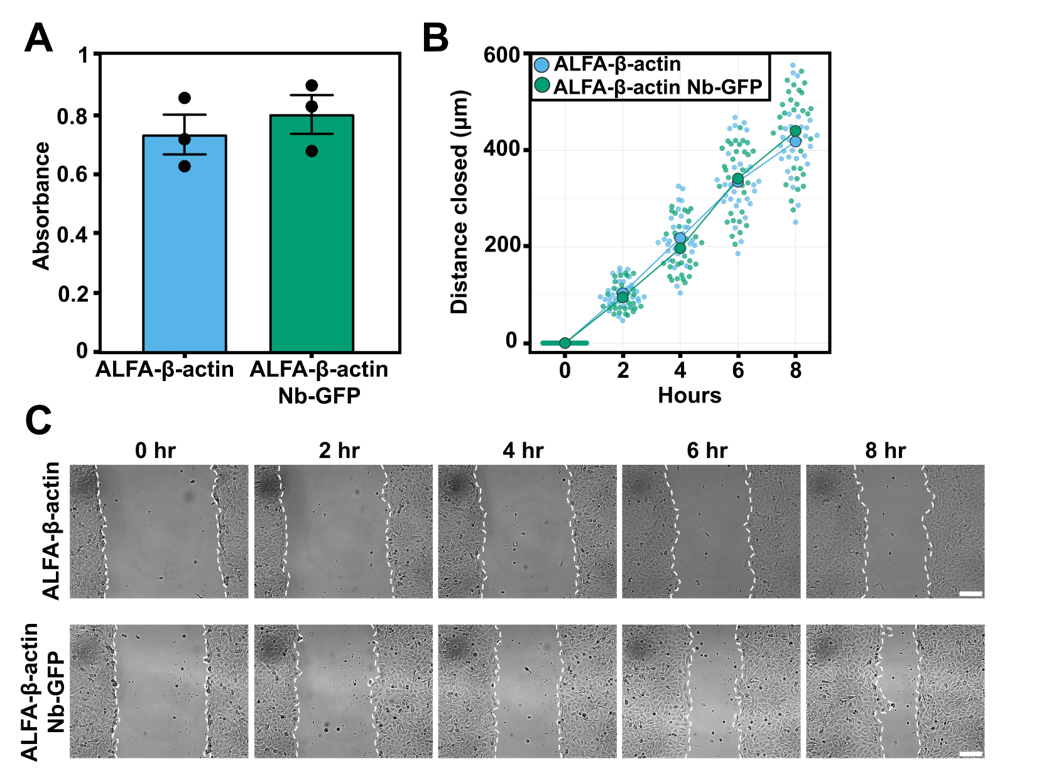

Supplement: S12 Fig — (A) Quantification of an MTT proliferation assay performed on ALFA-β-actin HT1080 cells with and without expressing the ALFA tag nanobody. Individual data points represent the average for 3 independent experiments. Bars represent the mean value and error bars represent standard error of mean (SEM). (B) Quantification of the wound closure assay shown in C indicating the distance closed in μm over time in ALFA-β-actin HT1080 cells and ALFA-β-actin HT1080 cells expressing ALFA-Nb-GFP. Large data points represent the mean of 3 independent experiments and the small data points represent the quantification of the individual images. Ten images per condition were acquired per experiment. (C) Representative widefield images of ALFA-β-actin HT1080 cells with and without expressing the ALFA tag nanobody at time point 0 h, 2 h, 4 h, 6 h, and 8 h after scratch induction. Scale bar: 30 μm. The numerical data underlying this figure can be found in S1 Data. (TIF) [file pbio.3002551.s012.tif]

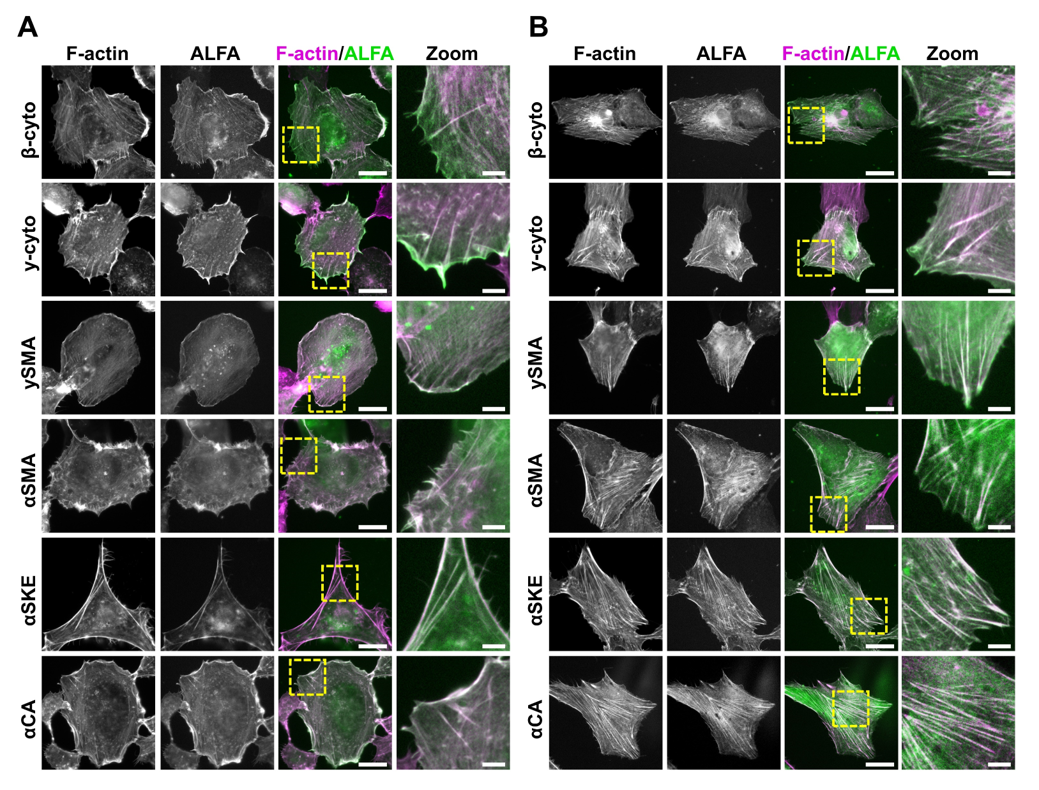

Supplement: S13 Fig — (A) Representative widefield immunofluorescence images of HT1080 with F-actin (magenta) and ALFA tag (green) in HT1080 cells that overexpress the ALFA tag in position T229/A230 in all 6 human actin isoforms. Scale bar: 15 μm. Scale bar zoom: 5 μm. (B) Representative widefield immunofluorescence images of RPE1 with F-actin (magenta) and ALFA tag (green) in HT1080 cells that overexpress the ALFA tag in position T229/A230 in all 6 human actin isoforms. Scale bar: 15 μm. Scale bar zoom: 5 μm. (TIF) [file pbio.3002551.s013.tif]

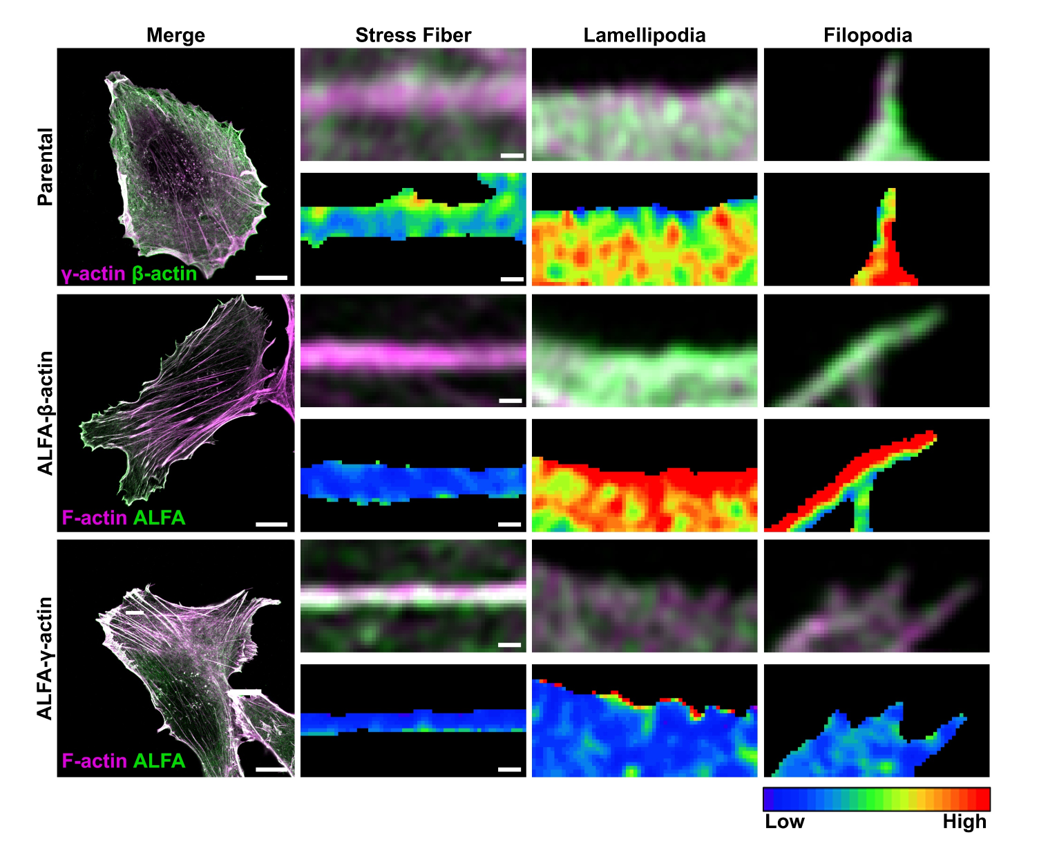

Supplement: S14 Fig — Representative Airyscan images of cells seeded on standard coverslips. Shown are parental HT1080 cells stained for β-actin (green) and γ-actin (magenta) and ALFA-β-actin and ALFA-γ-actin cells stained for ALFA tag (green) and F-actin (magenta). The zoom images present stress fibers, lamellipodia, and filopodia and the 32-color ratio images indicate the ratio between β- and γ-actin (parental cells) or the ratio between β- or γ-actin and total actin. Scale bar: 10 μm. Scale bar zoom images: 0.5 μm. (TIF) [file pbio.3002551.s014.tif]

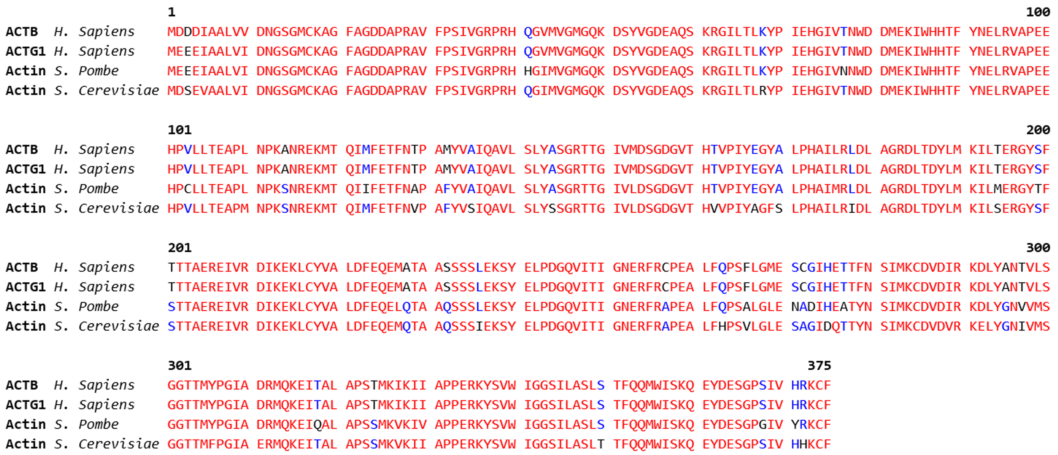

Supplement: S15 Fig — Red residues indicate complete consensus among variants, blue indicates 1 substitution with the substitution indicated in black, and black columns indicates 2 substitutions. (TIF) [file pbio.3002551.s015.tif]

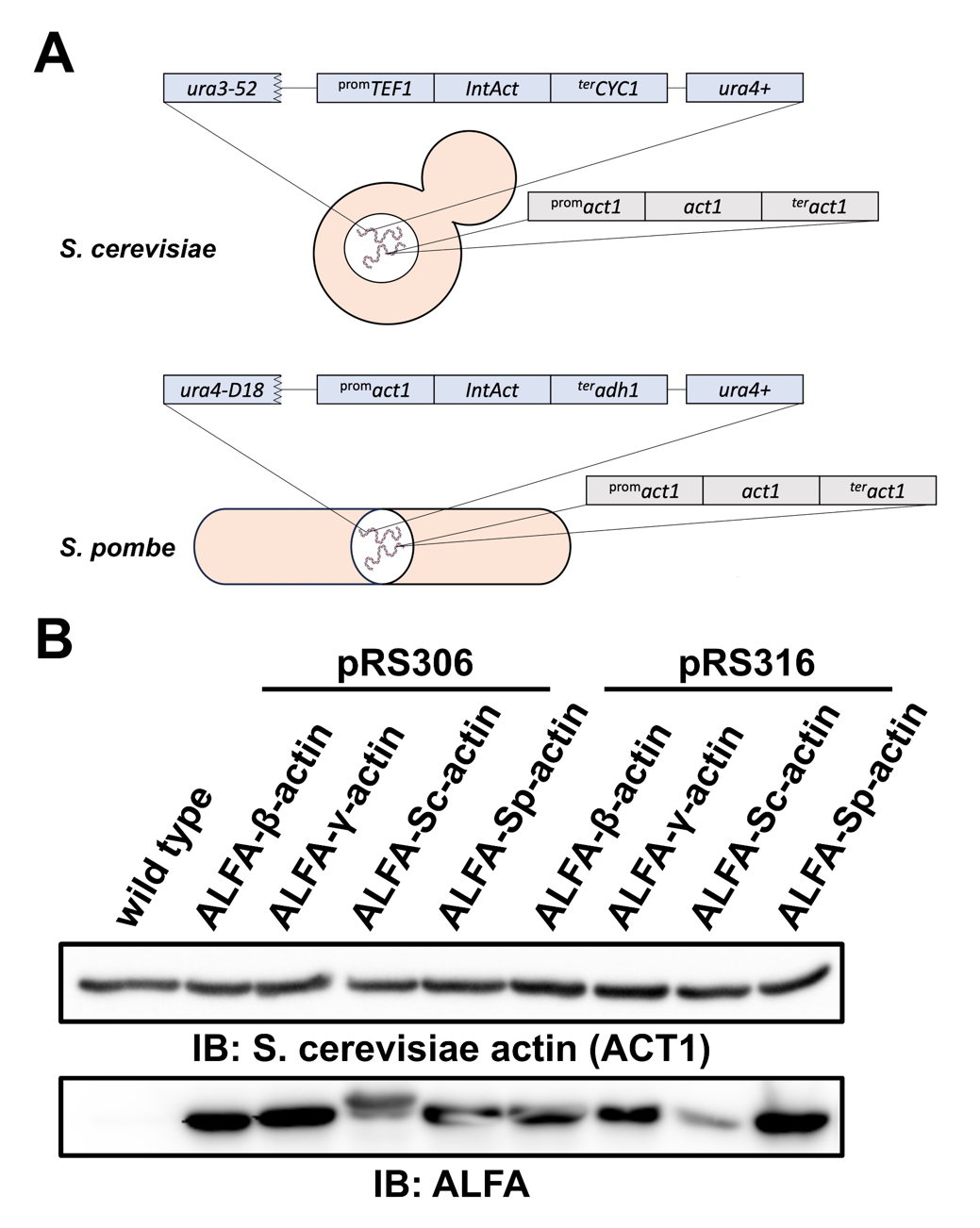

Supplement: S16 Fig — (A) Schematic overview of budding yeast (S. cerevisiae, top) and fission yeast (S. pombe, bottom) showing an extra copy of IntAct actins (integrated at auxotrophic marker locus or present in plasmid) in addition to the native yeast actin at its native locus. (B) Representative western blot showing expression of endogenous S. cerevisiae actin (ACT1) and β-IntAct, γ-IntAct, Sc-IntAct, Sp-IntAct expressed from either an integrating plasmid (pRS306) or centromeric plasmid (pRS316). (TIF) [file pbio.3002551.s016.tif]

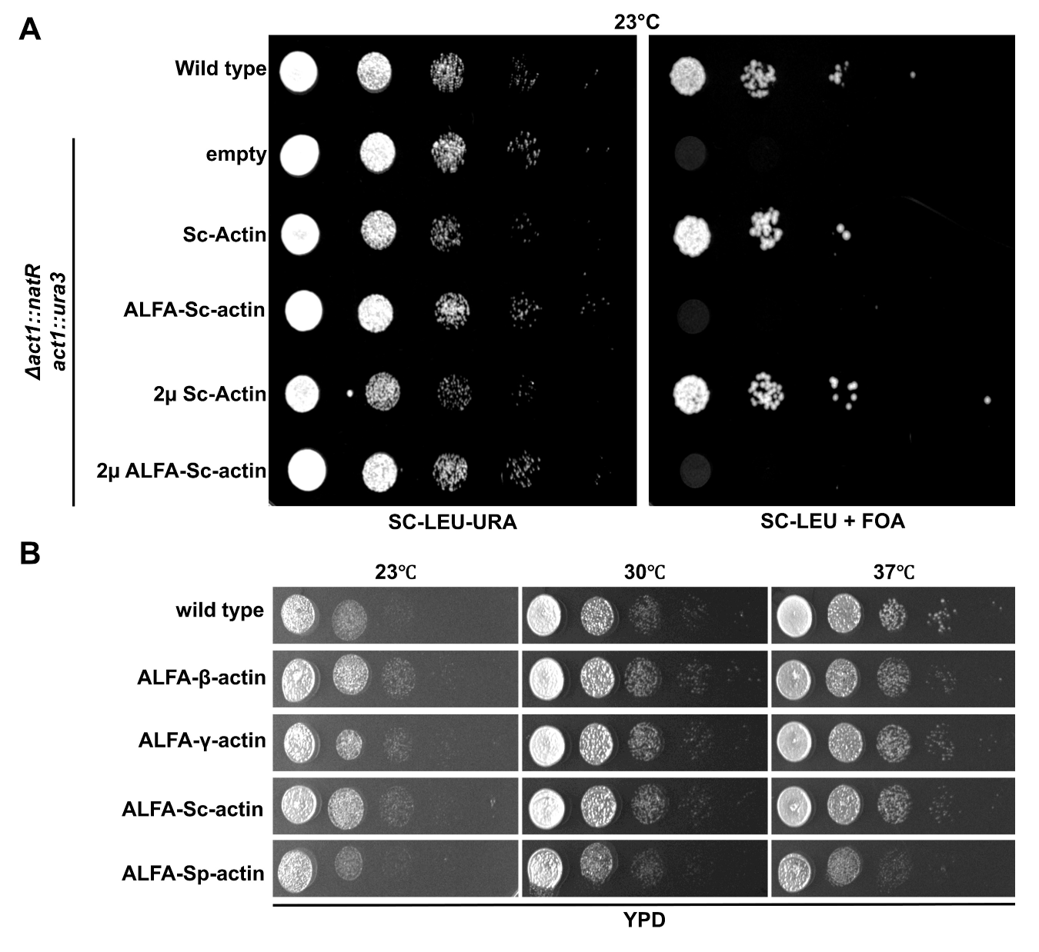

Supplement: S17 Fig — (A) Spot assay image showing strains co-expressing budding yeast native act1 with ALFA-Sc-actin in a low-copy and high-copy plasmid expression (2μ) (left) and spot assay image showing strains expressing ALFA-Sc-actin in a low-copy and high-copy plasmid expression (2μ) in the absence of native actin which was shuffled out using 5′-FOA media (right). (B) Spot assay image of strains co-expressing IntAct proteins with respect to wild-type strain. (TIF) [file pbio.3002551.s017.tif]

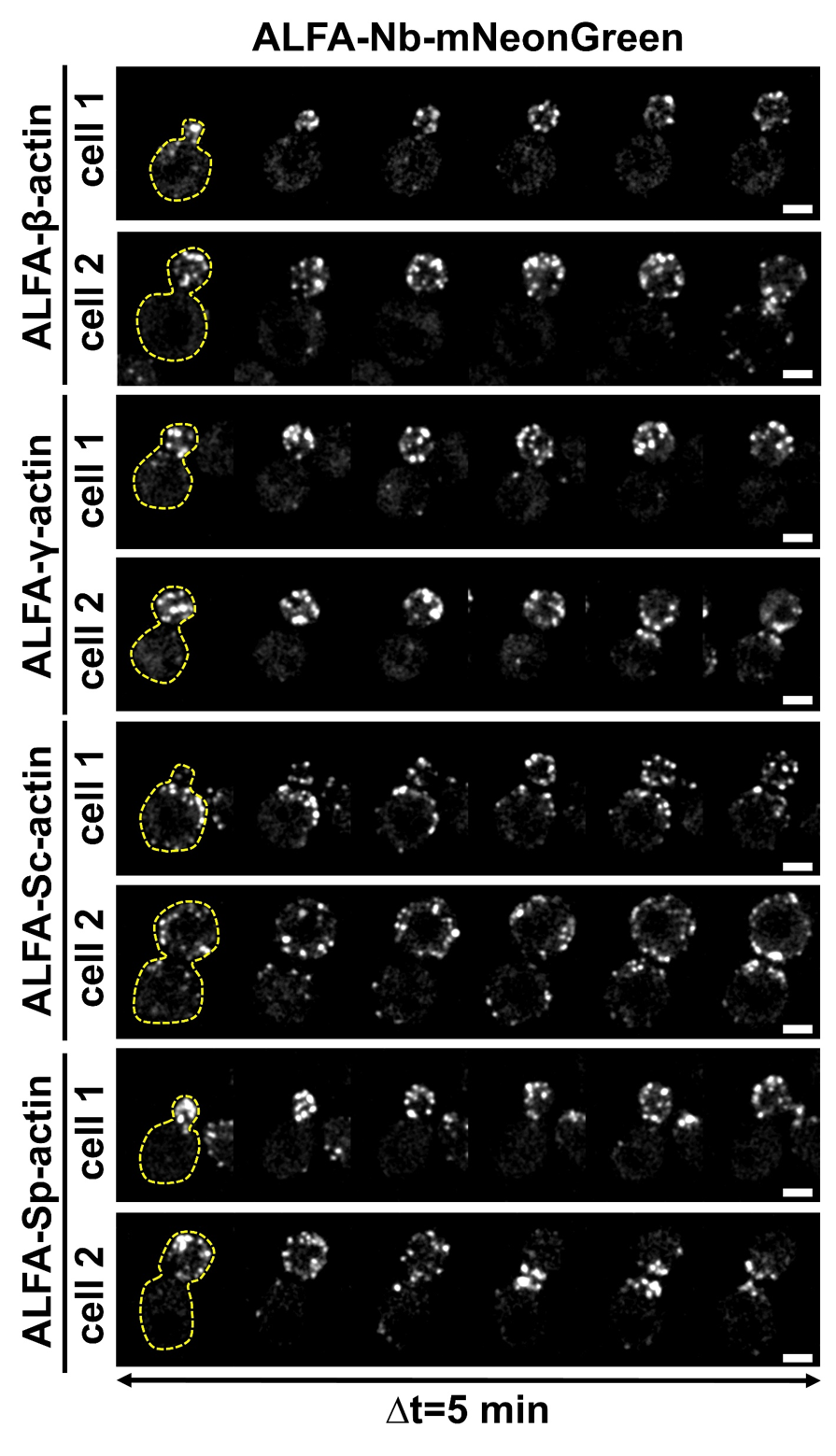

Supplement: S18 Fig — Representative confocal images of time-lapse imaging of ALFA-Nb-mNeonGreen expressing budding yeast cells co-expressing β-IntAct, γ-IntAct, Sc-IntAct, and Sp-IntAct. Yellow dashed line indicates the outline of the yeast cell. Scale bar: 3 μm. (TIF) [file pbio.3002551.s018.tif]

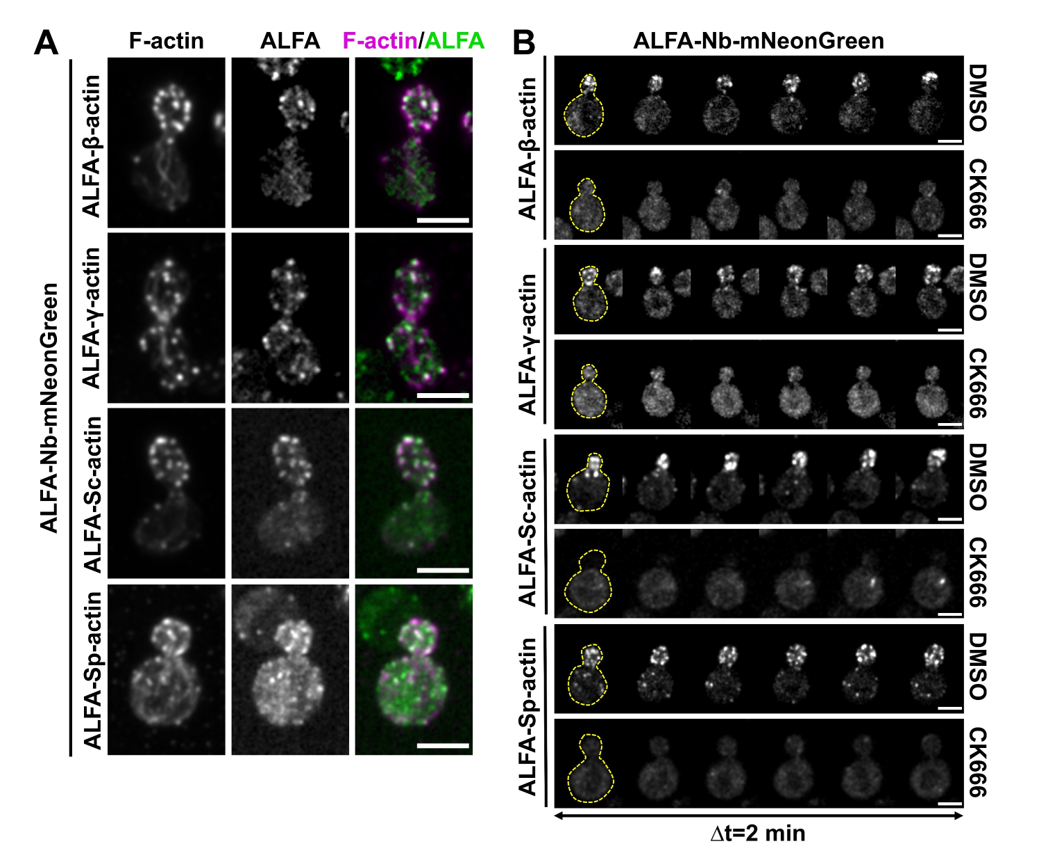

Supplement: S19 Fig — (A) Representative confocal images of ALFA-Nb-mNeonGreen (green) expressing budding yeast cells co-expressing Sc-IntAct and Sp-IntAct. Stained for F-actin (magenta). Scale bar: 3 μm. (B) Representative montages of time-lapse imaging of ALFA-Nb-mNeonGreen expressing budding yeast cells co-expressing: β-IntAct and γ-IntAct treated by either DMSO or CK666 (200 μM). Yellow dashed line indicates the outline of the yeast cell. Scale bar: 3 μm. (TIF) [file pbio.3002551.s019.tif]

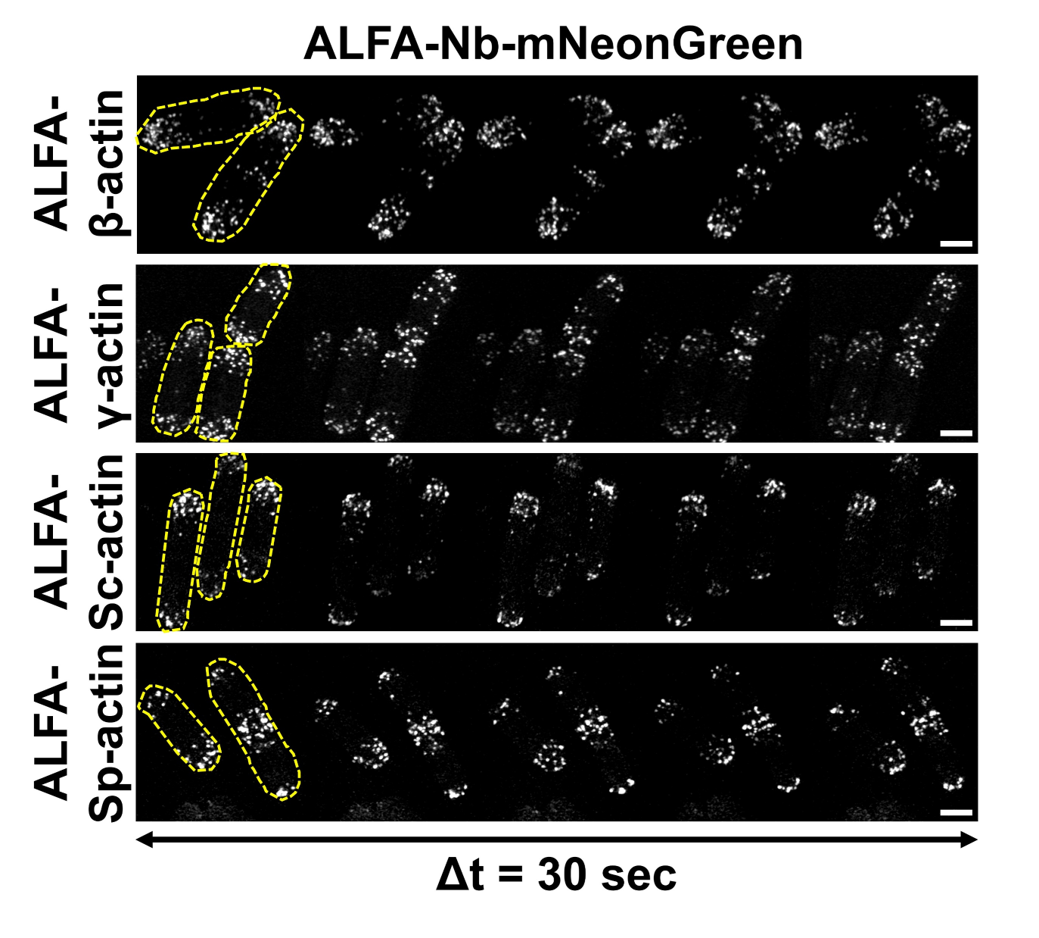

Supplement: S20 Fig — Representative confocal images of time-lapse imaging of ALFA-Nb-mNeonGreen expressing fission yeast cells co-expressing β-IntAct, γ-IntAct, Sc-IntAct, and Sp-IntAct. Yellow dashed line indicates the outline of the yeast cell. Scale bar: 3 μm. (TIF) [file pbio.3002551.s020.tif]

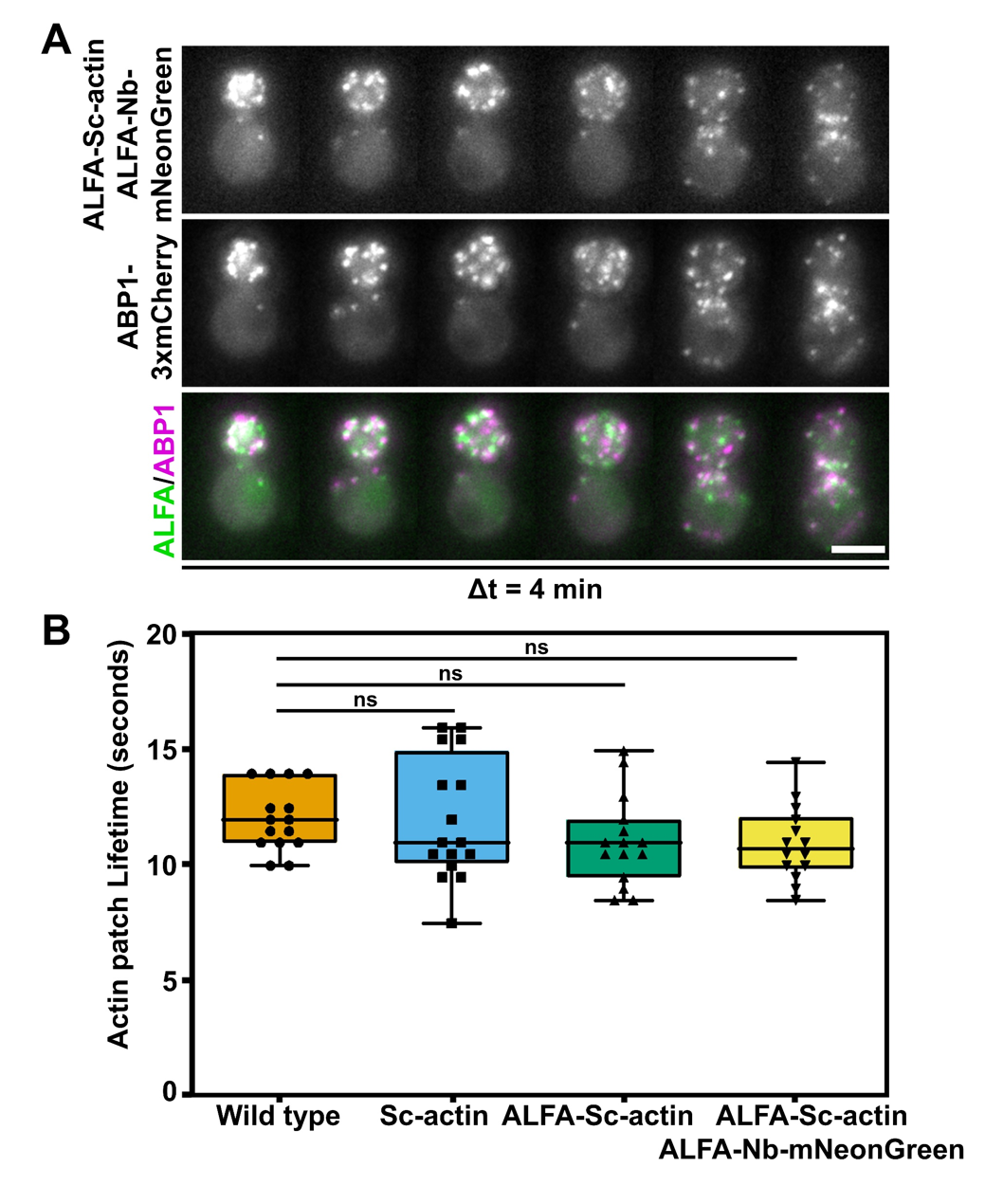

Supplement: S21 Fig — (A) Representative time-lapse montages of budding yeast cells constitutively co-expressing ALFA-Sc-actin and ALFA-Nb-mNG. Native Abp1-3xmCherry was used as an actin patch marker to assess colocalization. Scale Bar: 3 μm. (B) Quantification of actin patch lifetimes across the indicated strains. For each strain, more than 15 actin patches were measured. Statistical analysis was performed using a one-way ANOVA post hoc Tukey’s multiple comparison test. Box plots indicate median (middle line), 25th, 75th percentile (box) and minimum and maximum (whiskers). The numerical data underlying this figure can be found in S1 Data. (TIF) [file pbio.3002551.s021.tif]

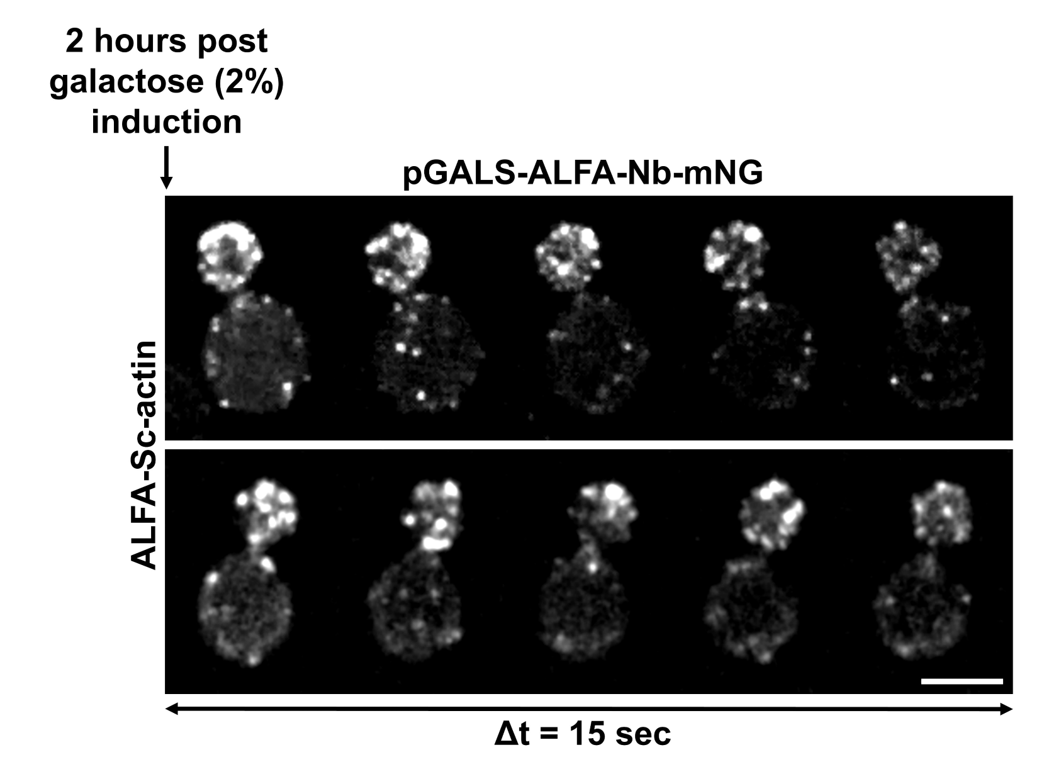

Supplement: S22 Fig — Representative time-lapse montages of budding yeast cells with constitutive expression Sc-IntAct and inducible expression of ALFA-Nb-mNeonGreen under GALS promoter. Galactose was added to a final concentration of 2% before start of imaging. Scale bar: 3 μm. (TIF) [file pbio.3002551.s022.tif]

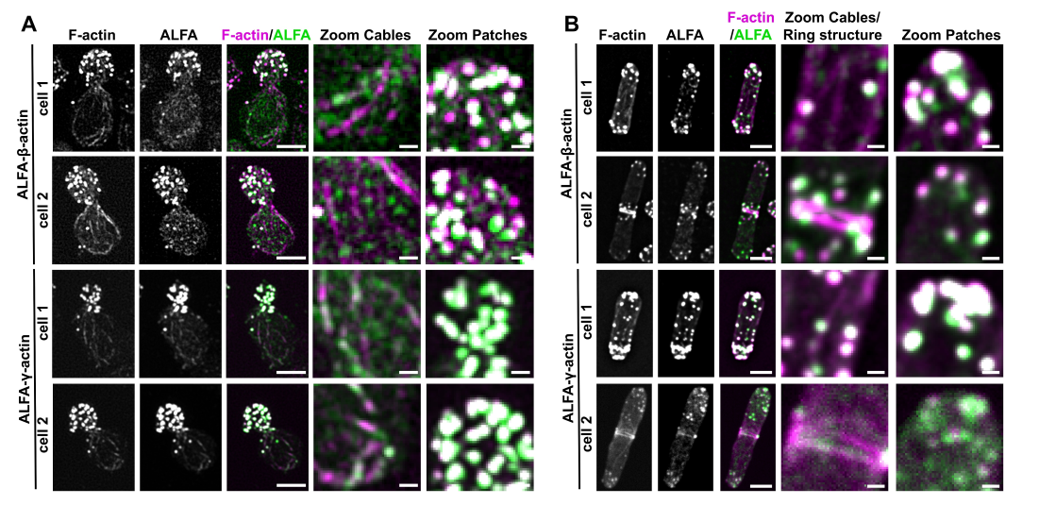

Supplement: S23 Fig — (A) Representative montages of budding yeast cells (S. cerevisiae) expressing β-IntAct and γ-IntAct. Cells were fixed and stained for F-actin (magenta) and ALFA tag nanobody (green). Scale bar: 3 μm. Scale bar zoom: 0.5 μm. (E) Representative montages of fission yeast cells (S. Pombe) expressing β-IntAct and γ-IntAct. Cells were fixed and stained for F-actin (magenta) and ALFA tag nanobody (green). Scale bar: 3 μm. Scale bar zoom: 0.5 μm. (TIF) [file pbio.3002551.s023.tif]

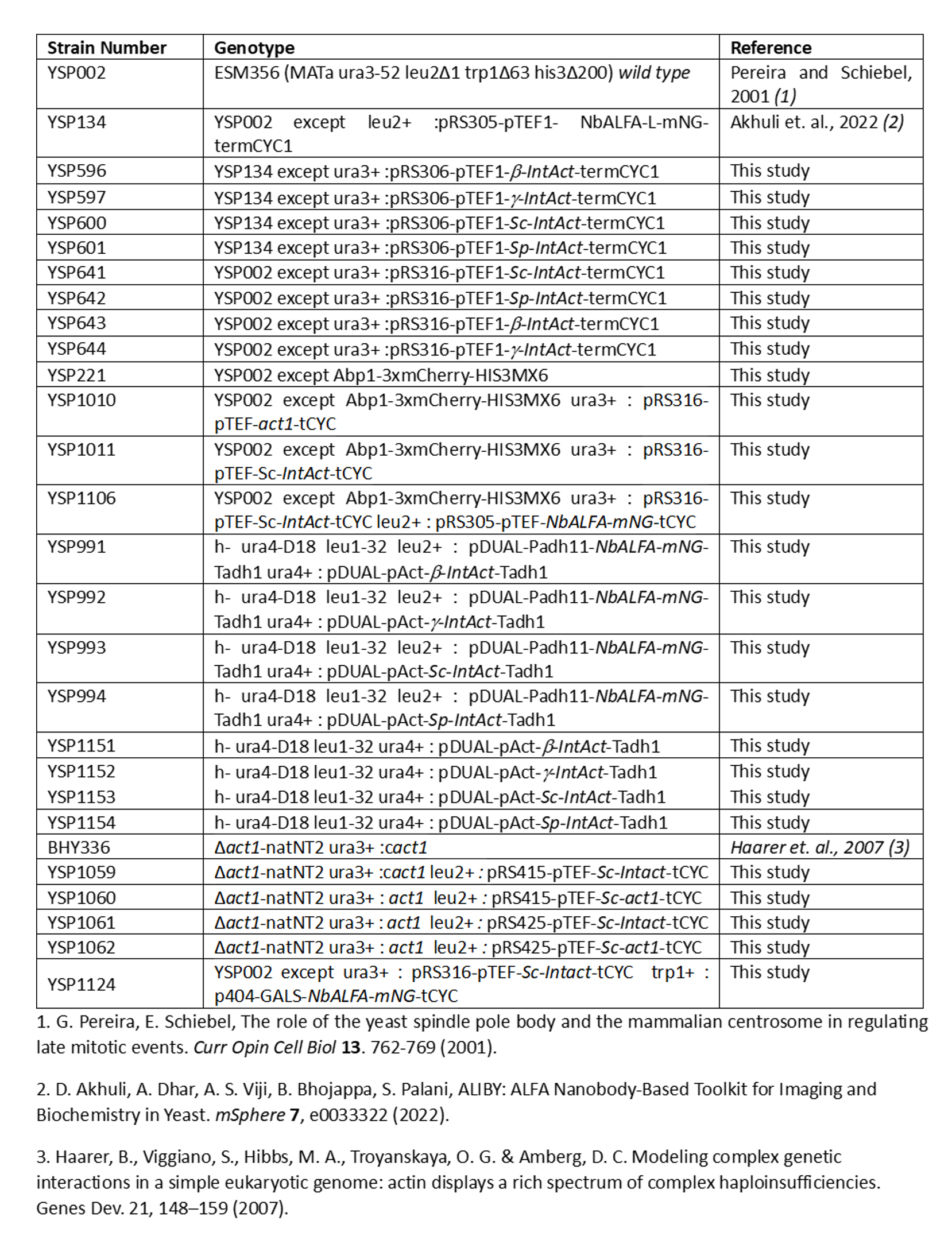

Supplement: S1 Table — (TIF) [file pbio.3002551.s024.tif]

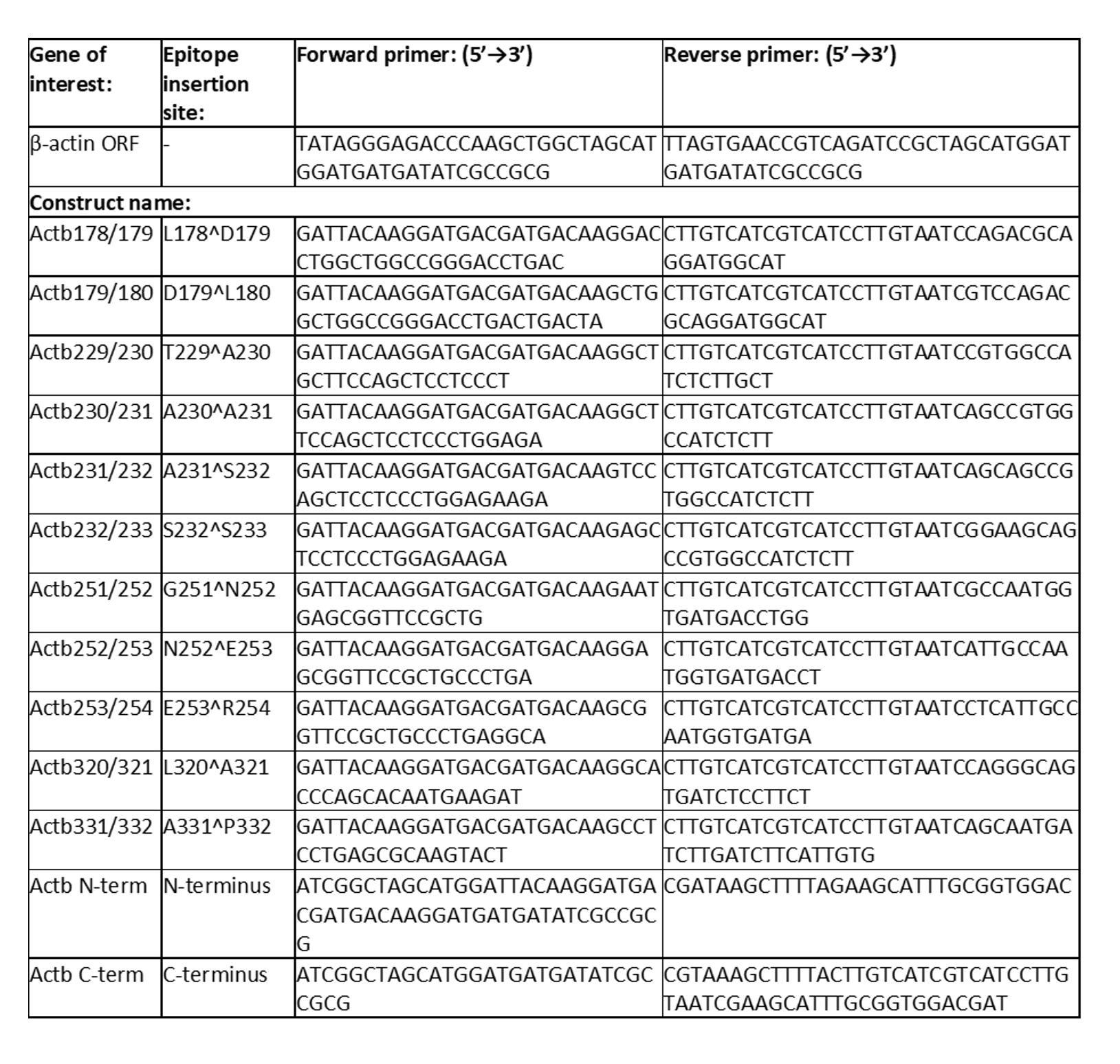

Supplement: S2 Table — (TIF) [file pbio.3002551.s025.tif]

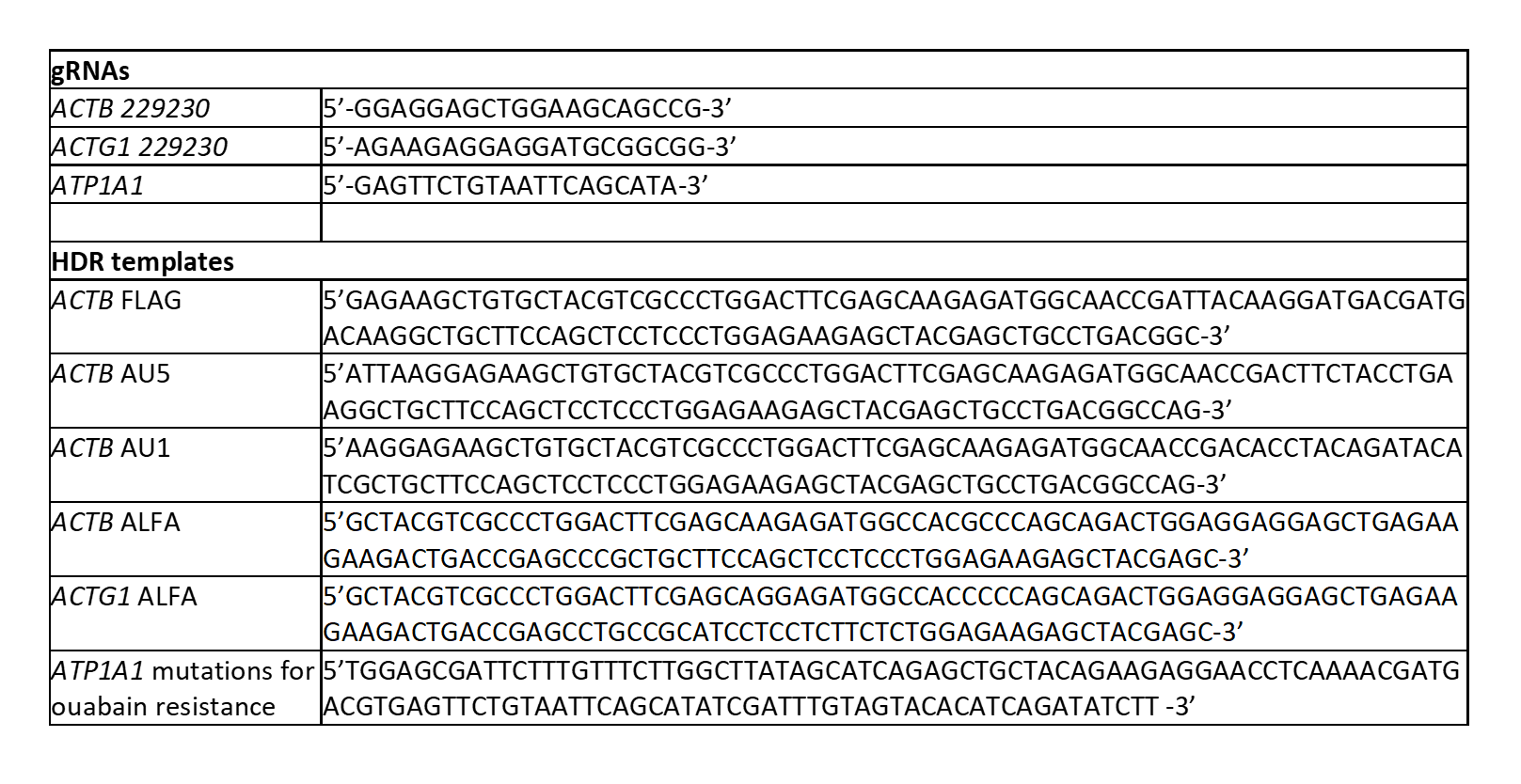

Supplement: S3 Table — All oligos were inserted into the px330-hSpCas9 vector (Addgene, 42230) and the vector with the gRNA targeting ATP1A1 was purchased (Addgene, 86611). (TIF) [file pbio.3002551.s026.tif]

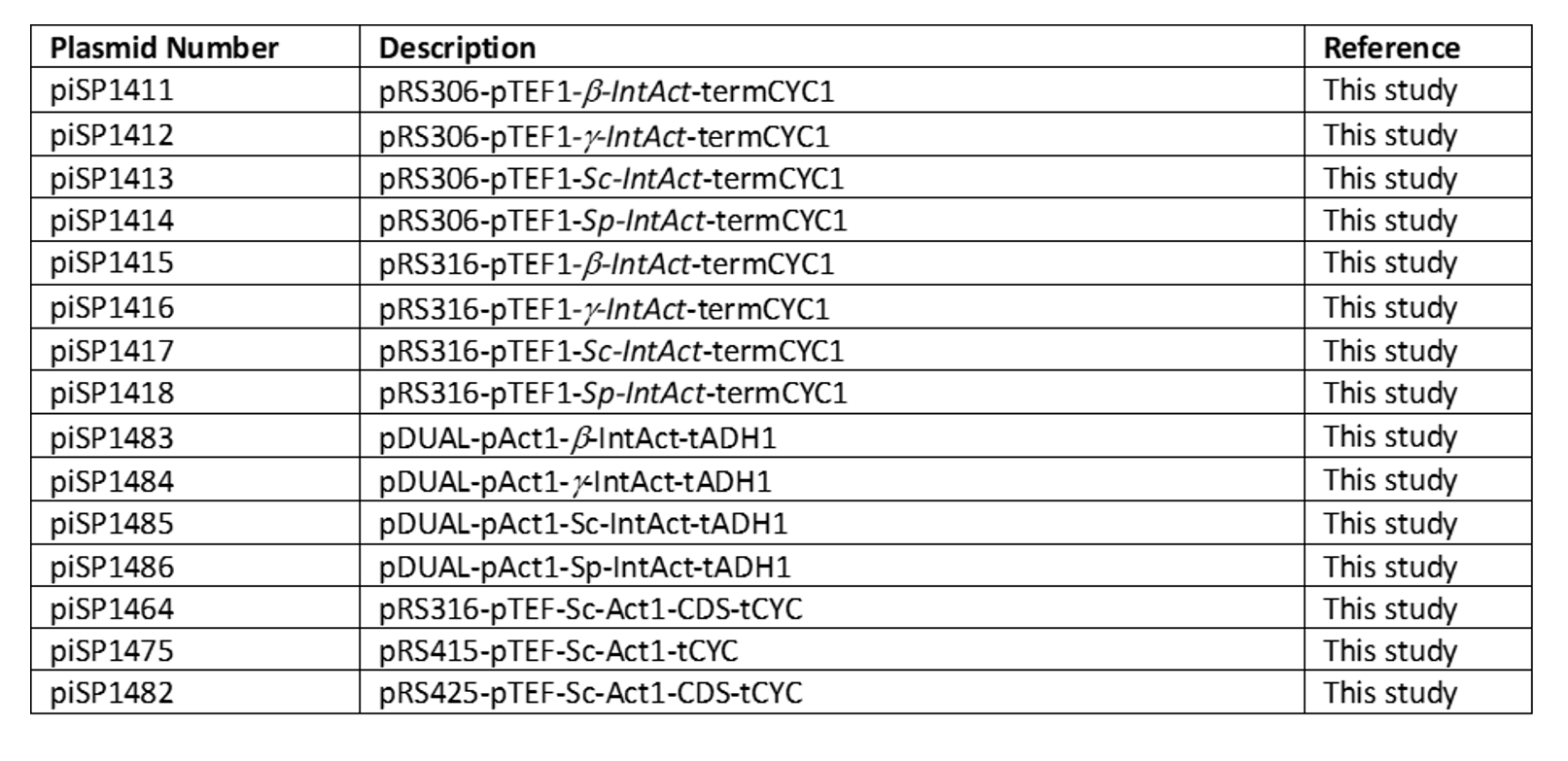

Supplement: S4 Table — (TIF) [file pbio.3002551.s027.tif]
