# Supplementary material for: IntAct: A nondisruptive internal tagging strategy to study the organization and function of actin isoforms
Source: PLoS Biol. 2024 Mar 11;22(3):e3002551. doi: 10.1371/journal.pbio.3002551 (PMC10957077; doi:10.1371/journal.pbio.3002551)

Fig 2A

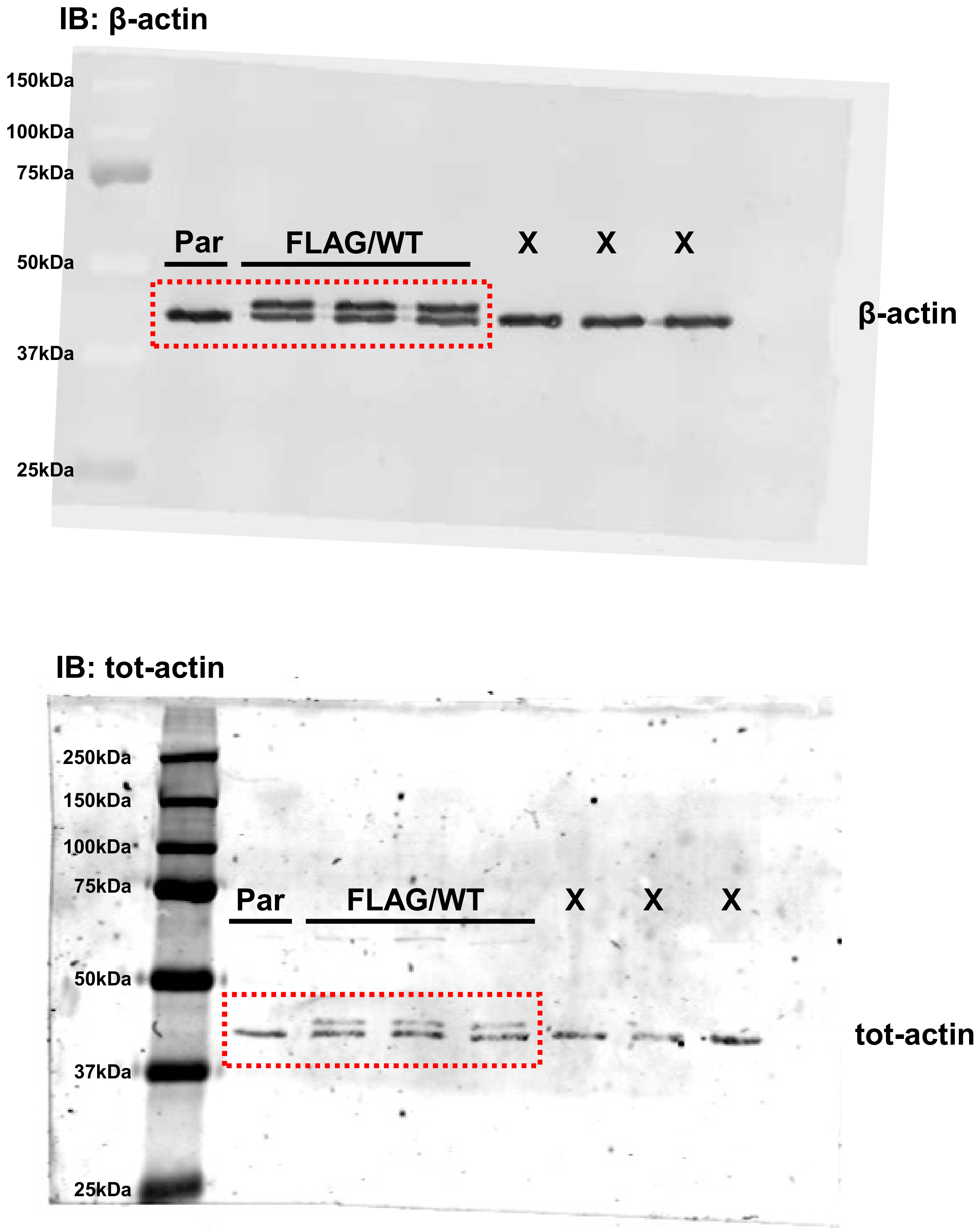

IB: β-actin
150kDa
100kDa
75kDa
50kDa
37kDa
25kDa
Par
FLAG/WT
X
X
X
β-actin
IB: tot-actin
250kDa
150kDa
100kDa
75kDa
50kDa
37kDa
25kDa
Par
FLAG/WT
X
X
X
tot-actin

Fig 2A

IB: γ-actin

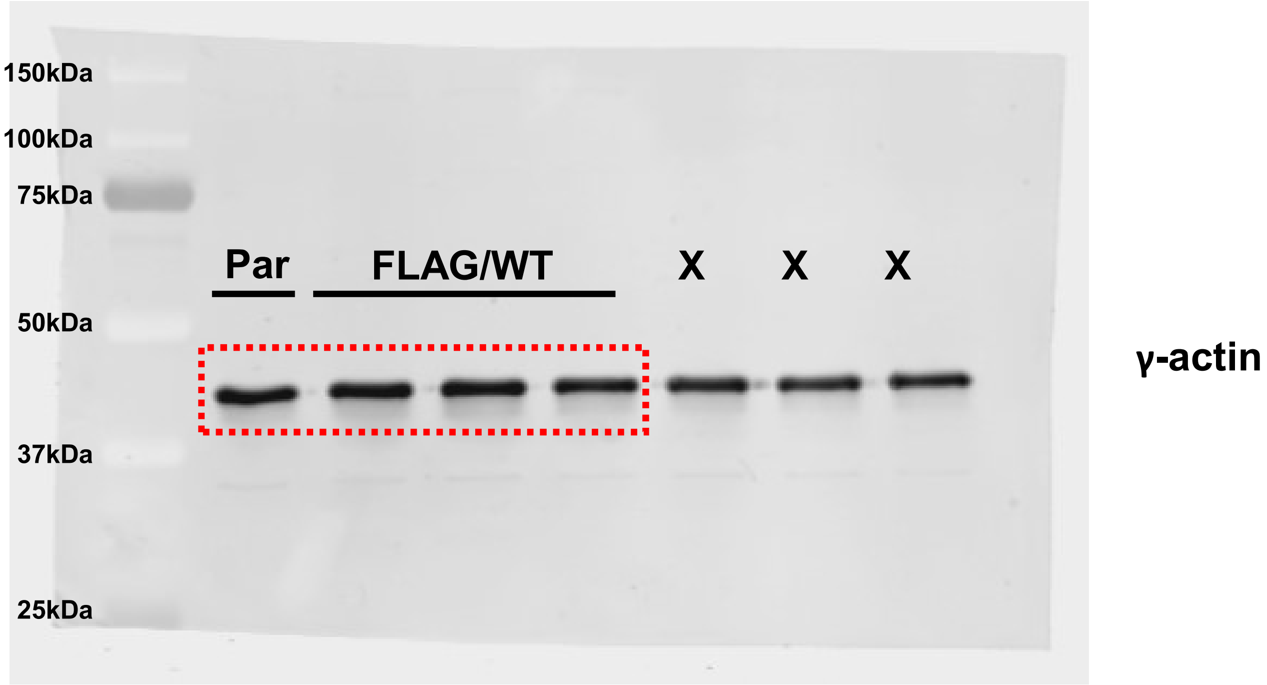

Fig 2C

IB: β-actin and Tubulin

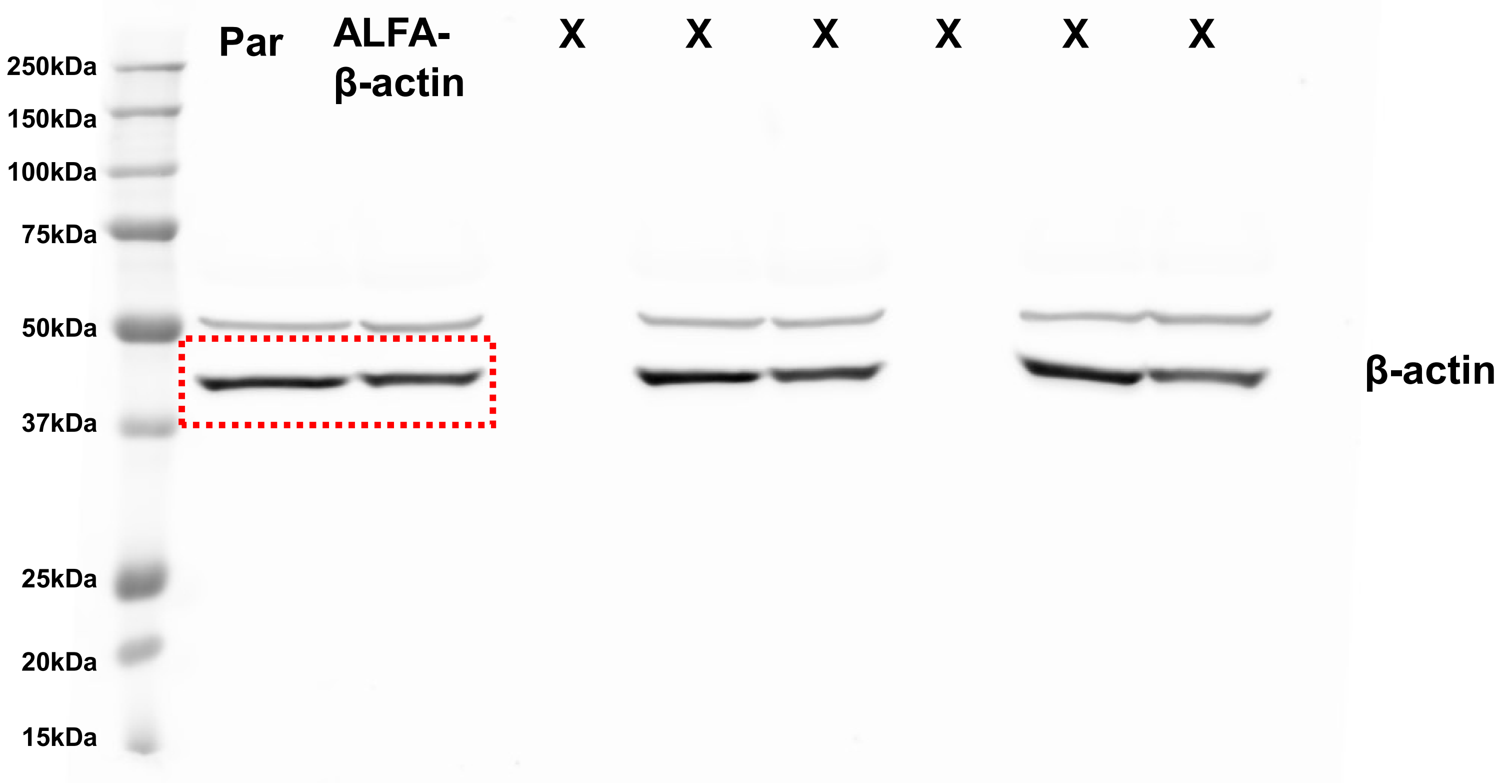

IB: β-actin and Tubulin

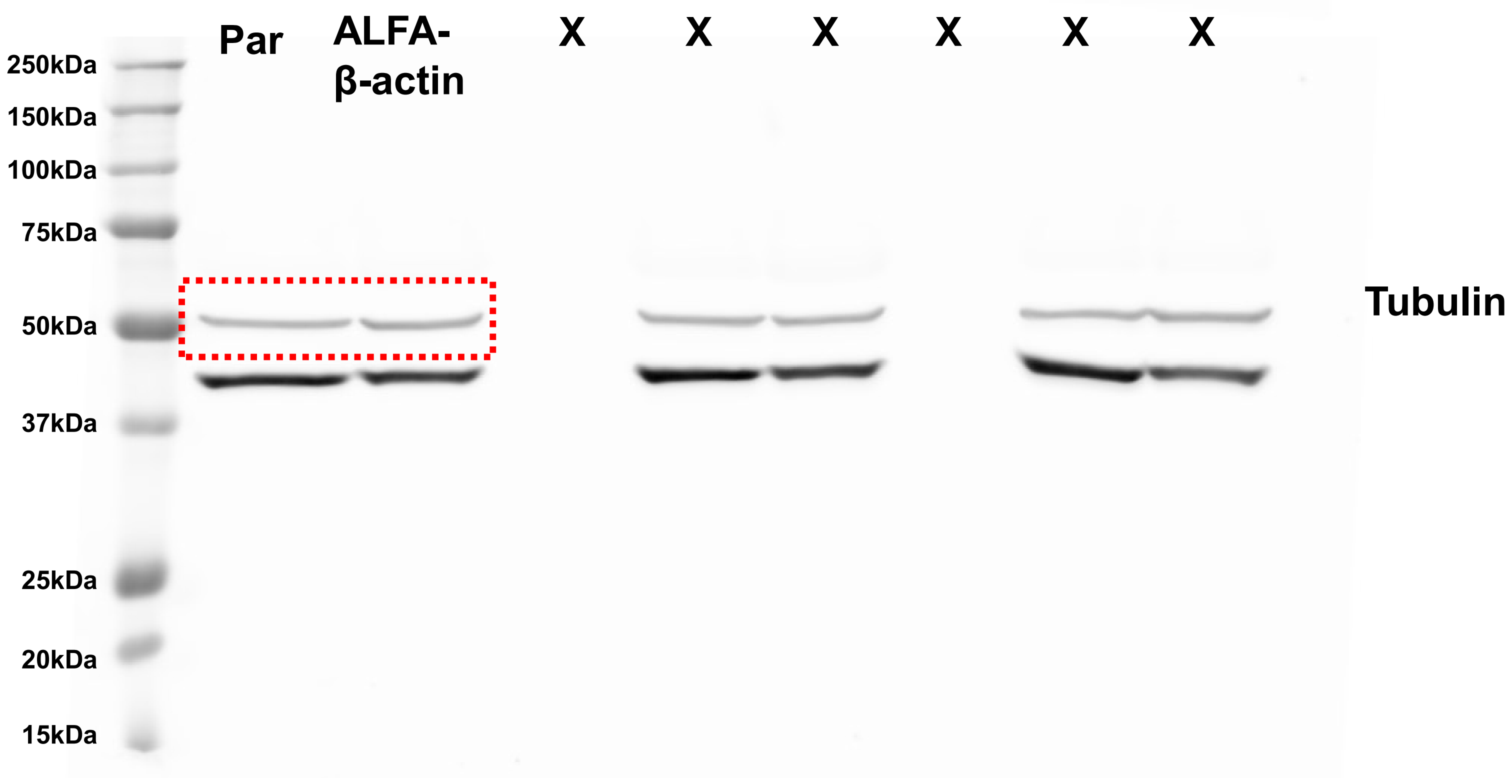

Fig 2G

IB: β-actin

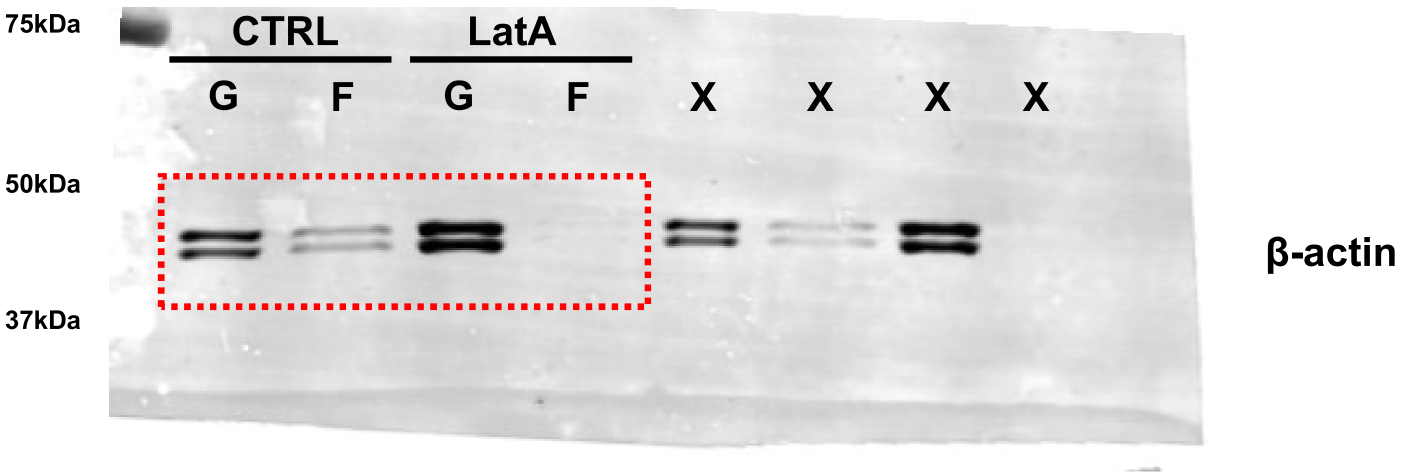

Fig 3A

IB: FLAG

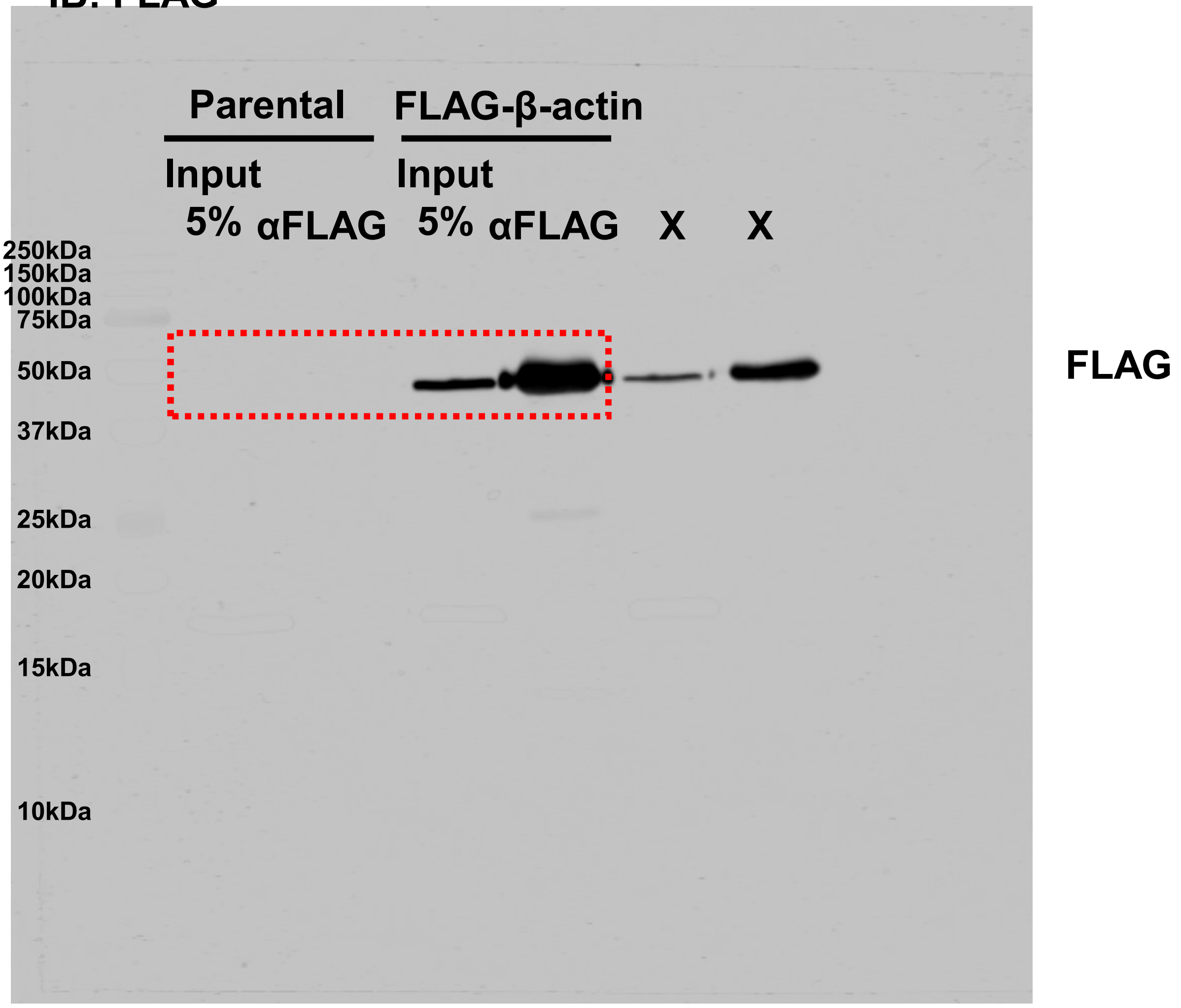

IB: Profilin

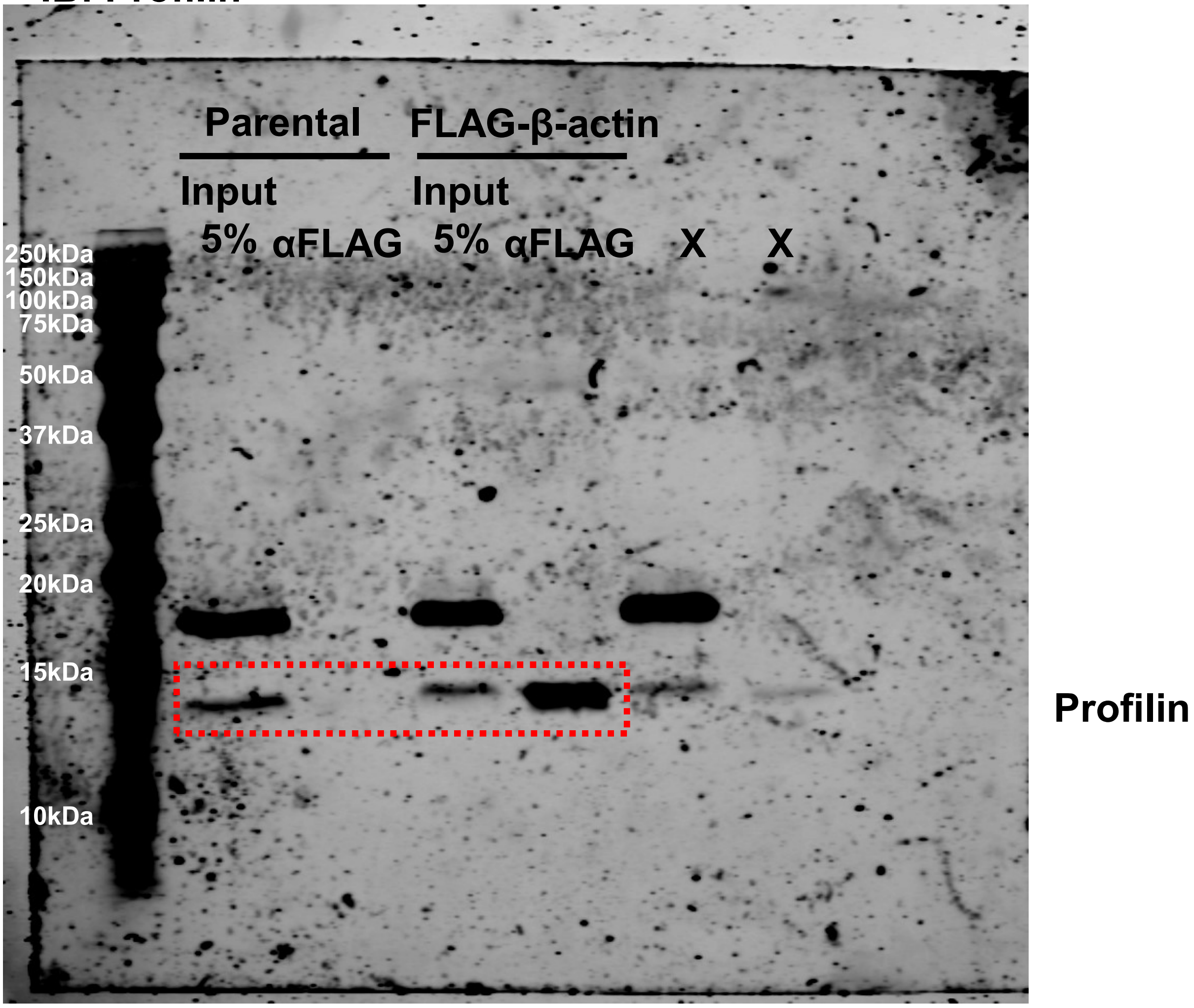

Fig 3B

IB: FLAG

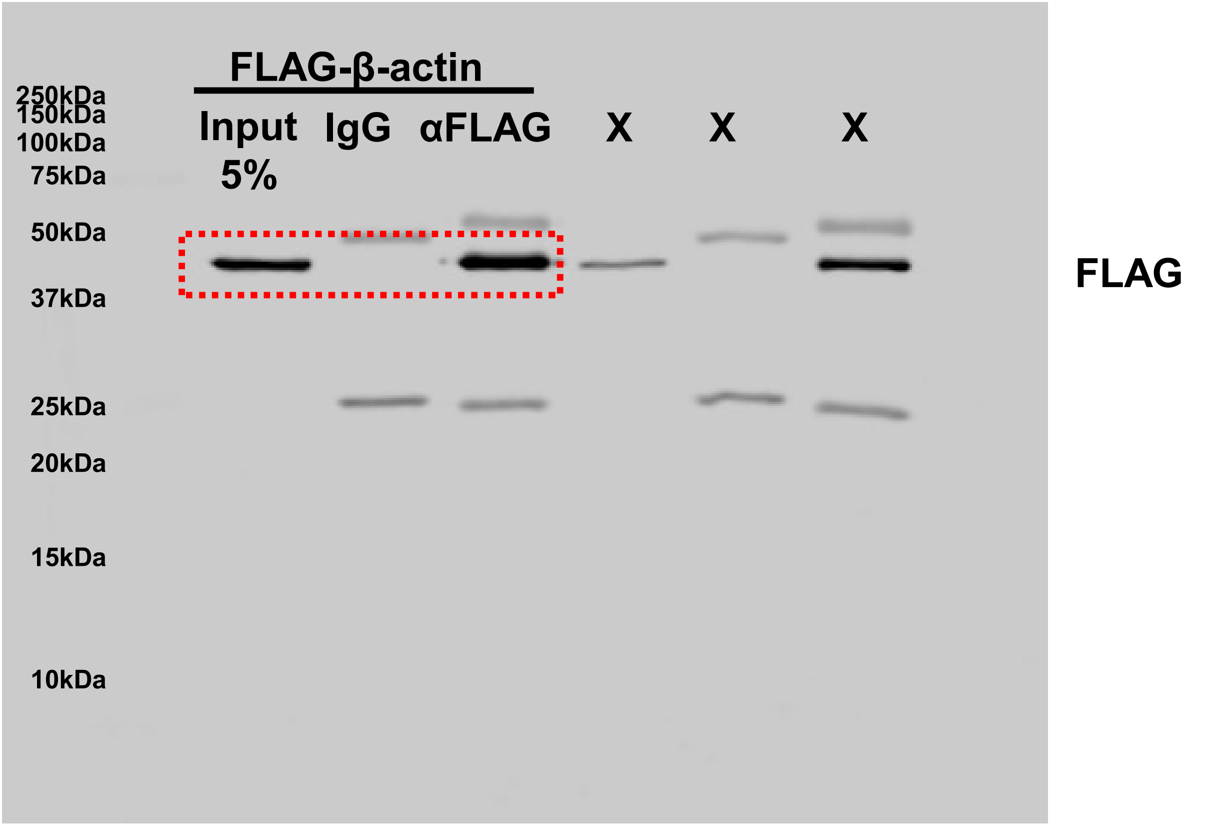

IB: Cofilin

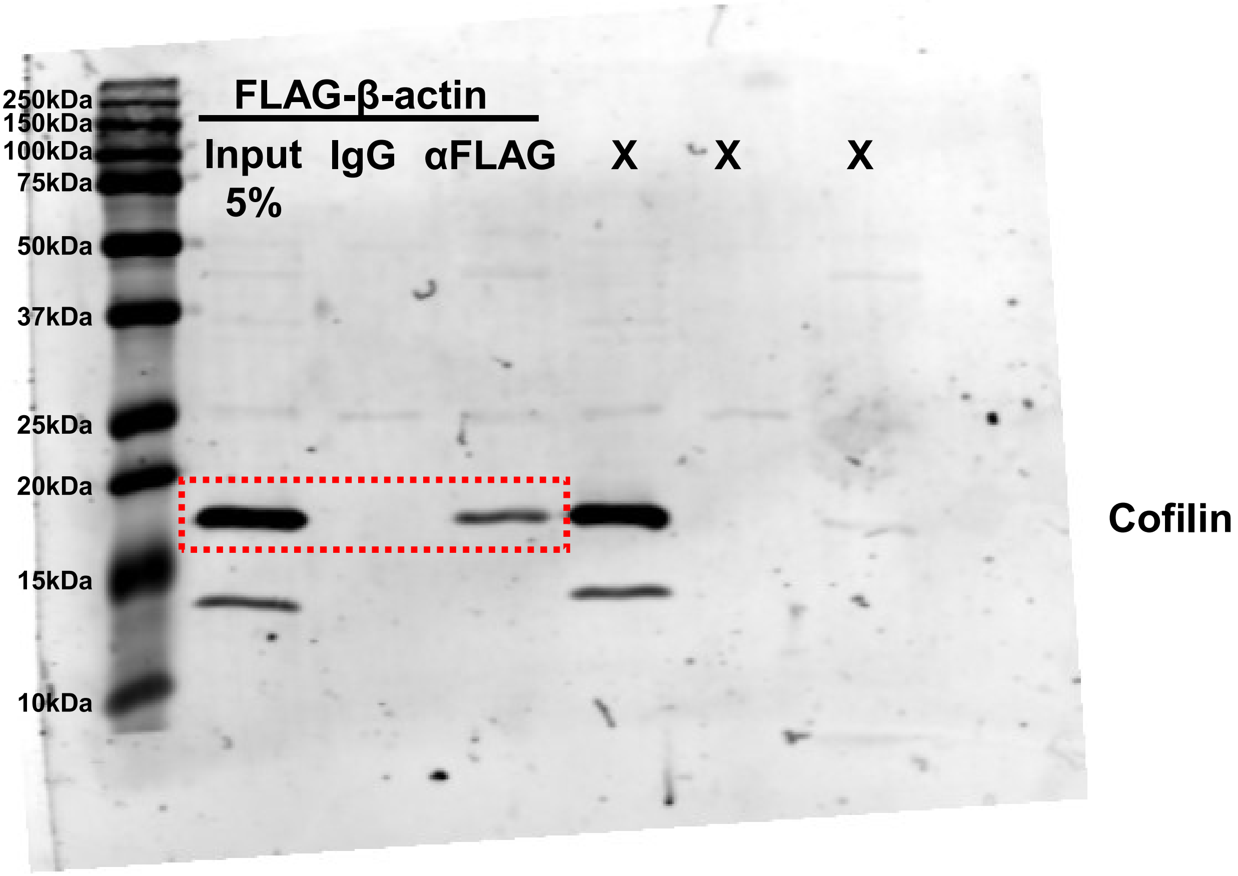

Fig 3C

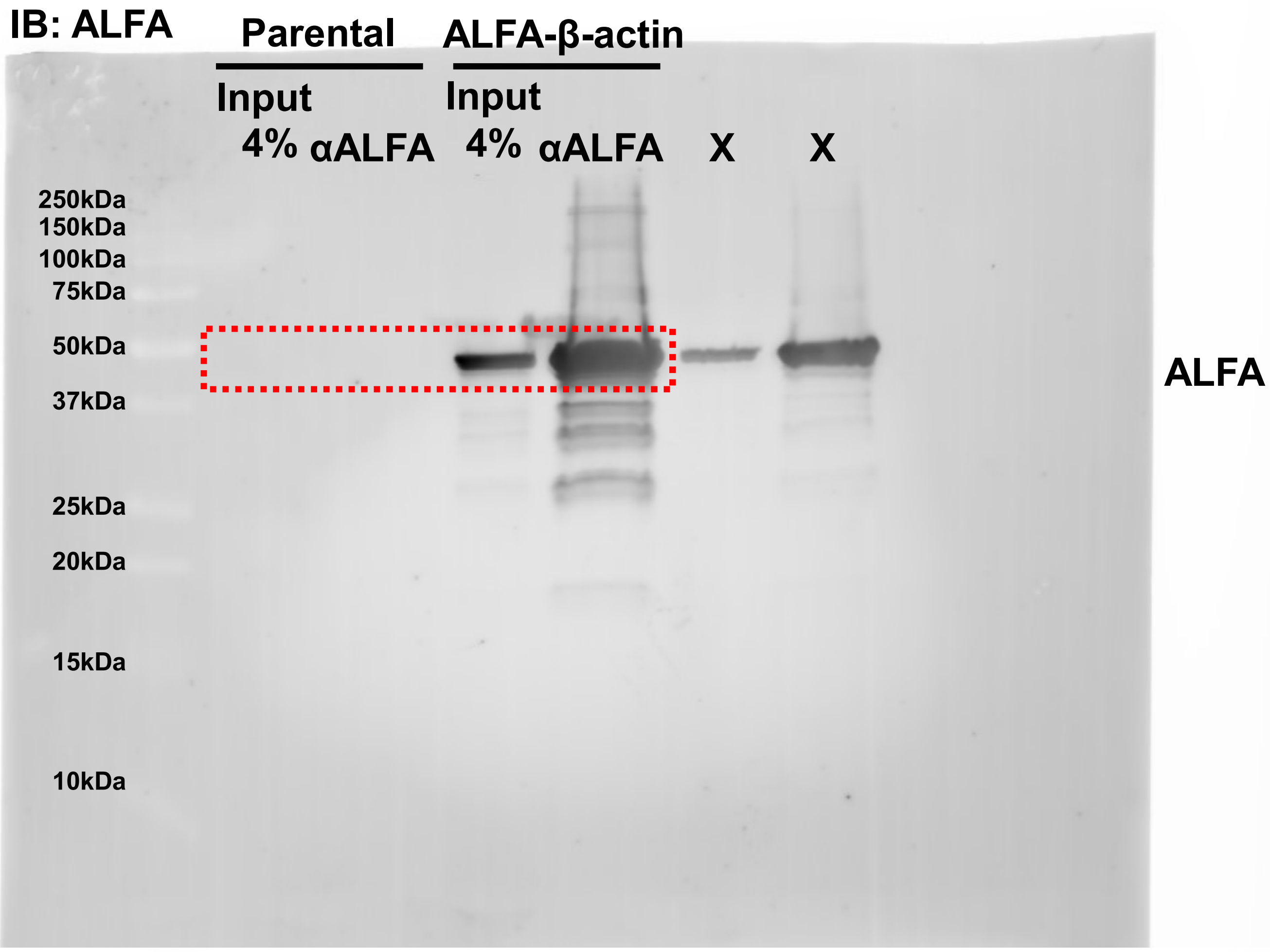
IB: ALFA
Parental
ALFA-β-actin
Input
4%
αALFA
Input
4%
αALFA
X
X
250kDa
150kDa
100kDa
75kDa
50kDa
37kDa
25kDa
20kDa
15kDa
10kDa
ALFA

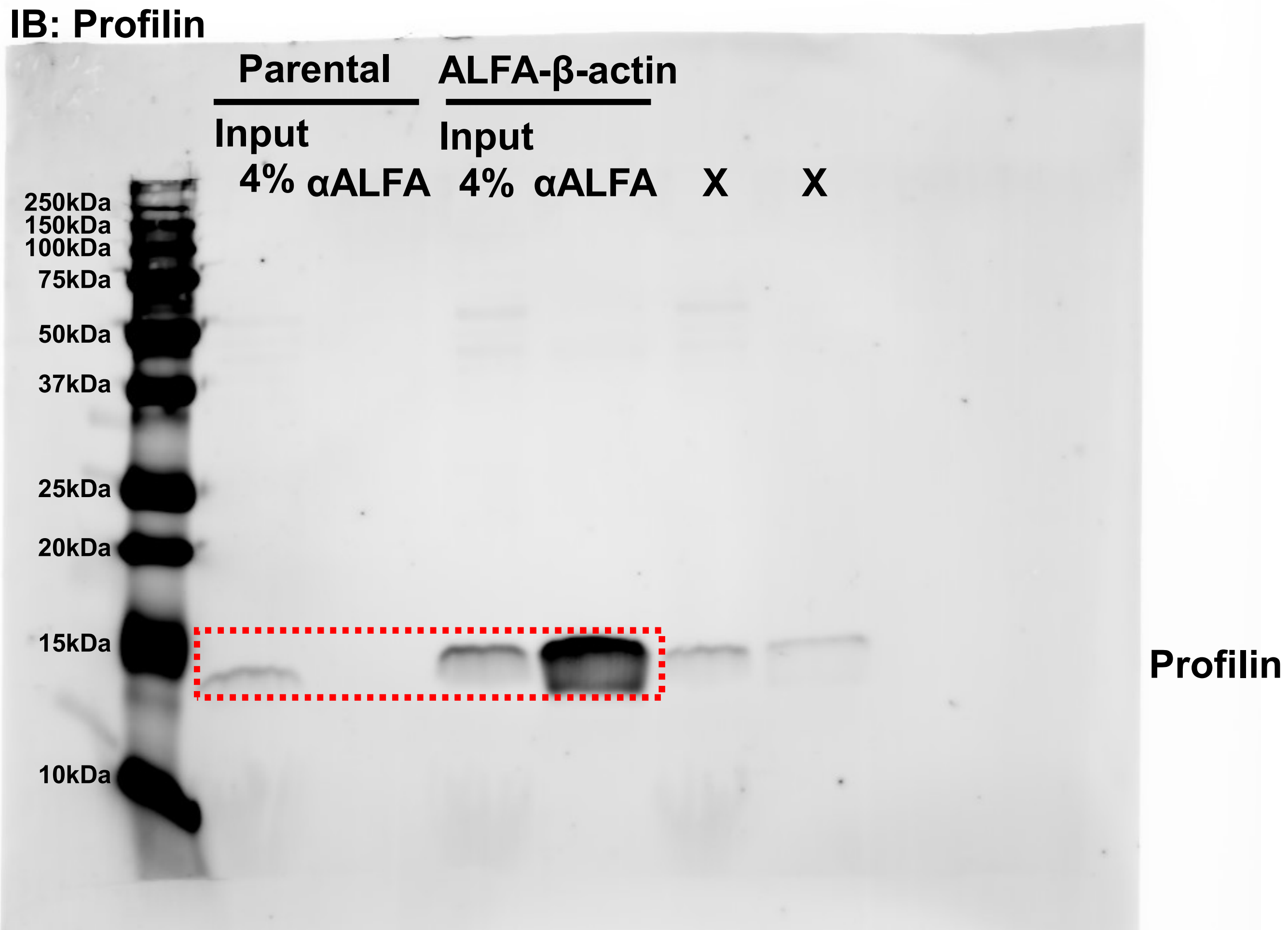
IB: Profilin
Parental
ALFA-β-actin
Input
4%
αALFA
Input
4%
αALFA
X
X
250kDa
150kDa
100kDa
75kDa
50kDa
37kDa
25kDa
20kDa
15kDa
10kDa
Profilin

Fig 3C

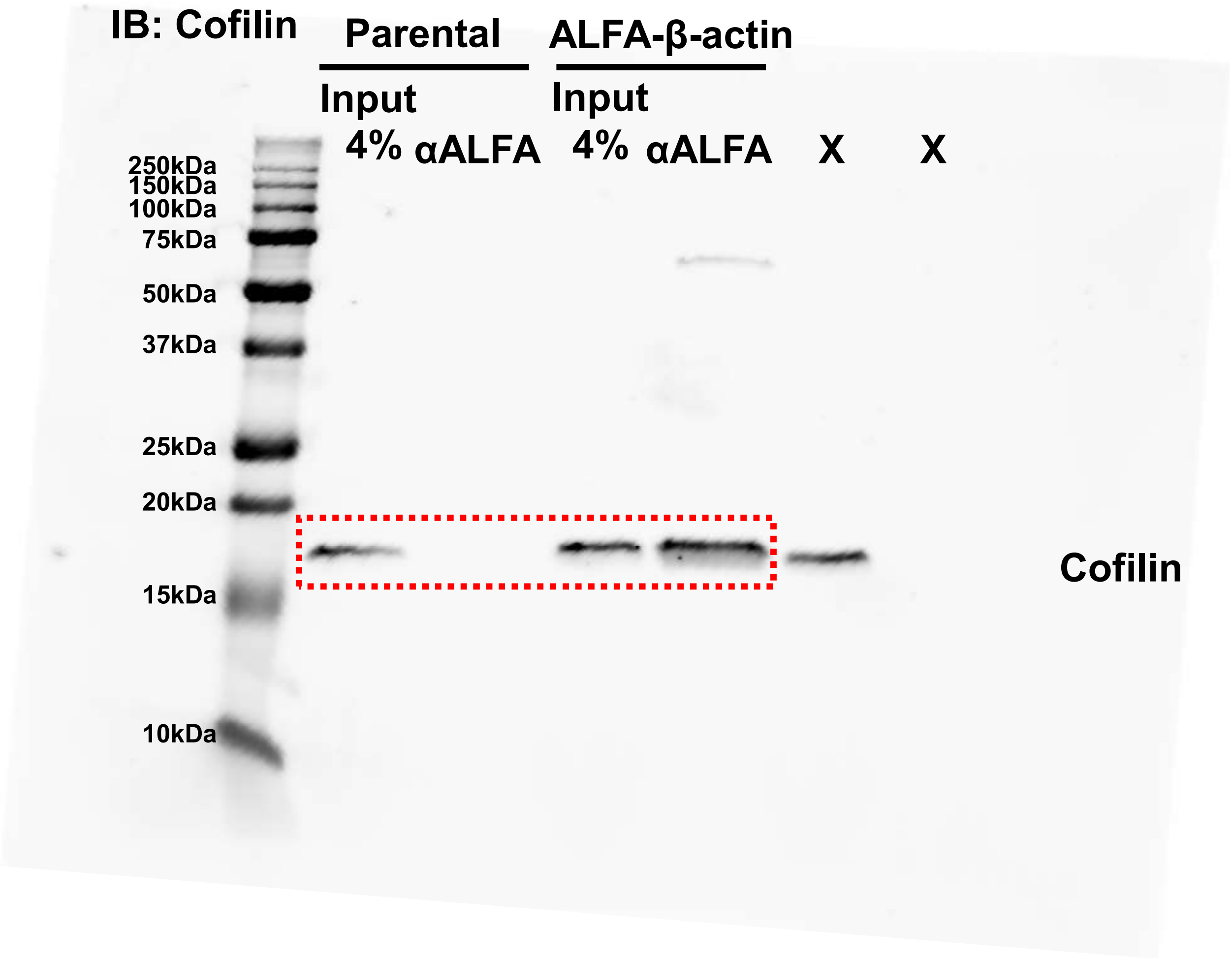
IB: Cofilin
Parental
ALFA-β-actin
Input
Input
4%
αALFA
4%
αALFA
X
X
250kDa
150kDa
100kDa
75kDa
50kDa
37kDa
25kDa
20kDa
15kDa
10kDa
Cofilin

Fig 3D

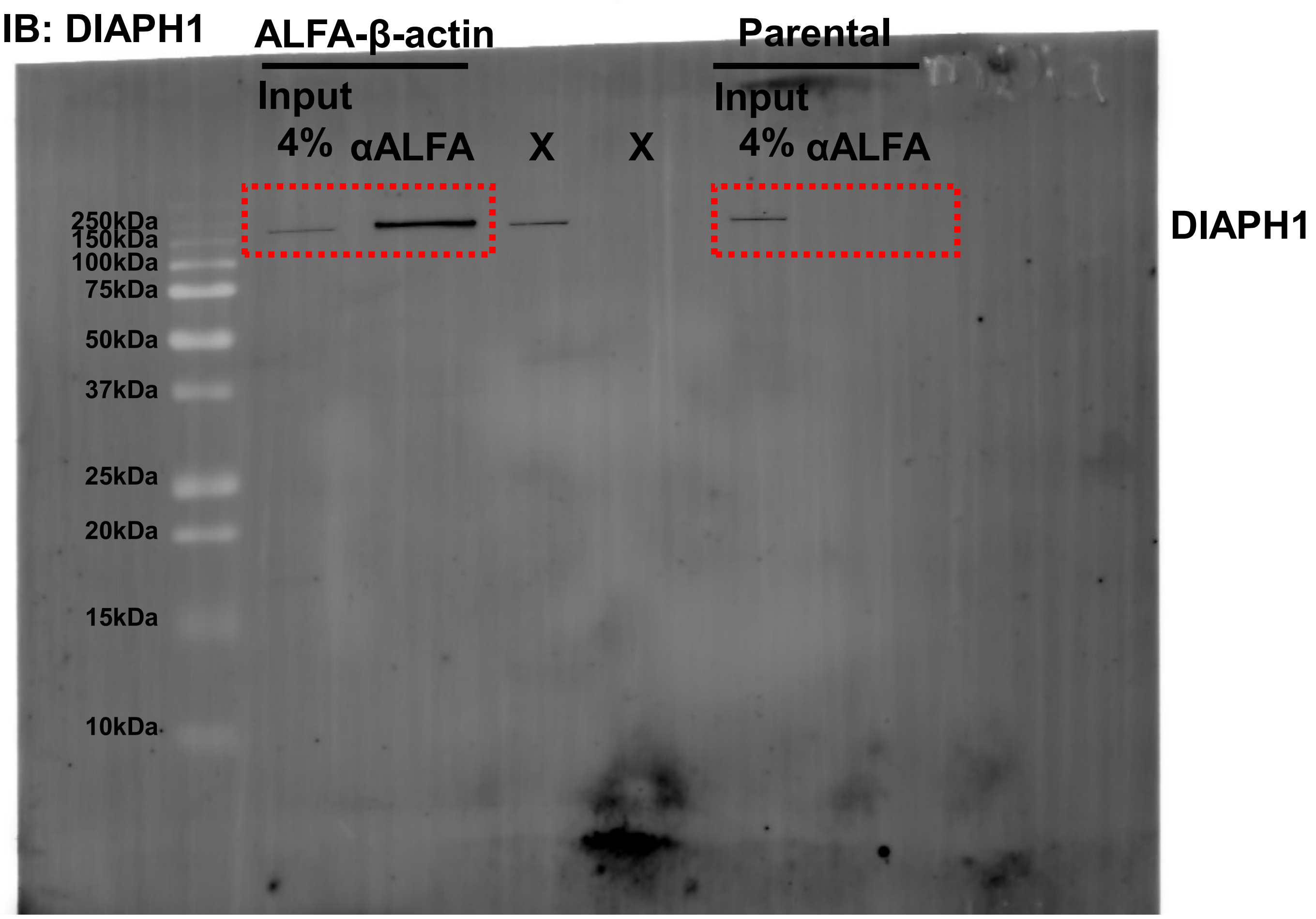
IB: DIAPH1
ALFA-β-actin
Parental
Input
4%
αALFA
X
X
Input
4%
αALFA
250kDa
150kDa
100kDa
75kDa
50kDa
37kDa
25kDa
20kDa
15kDa
10kDa
DIAPH1

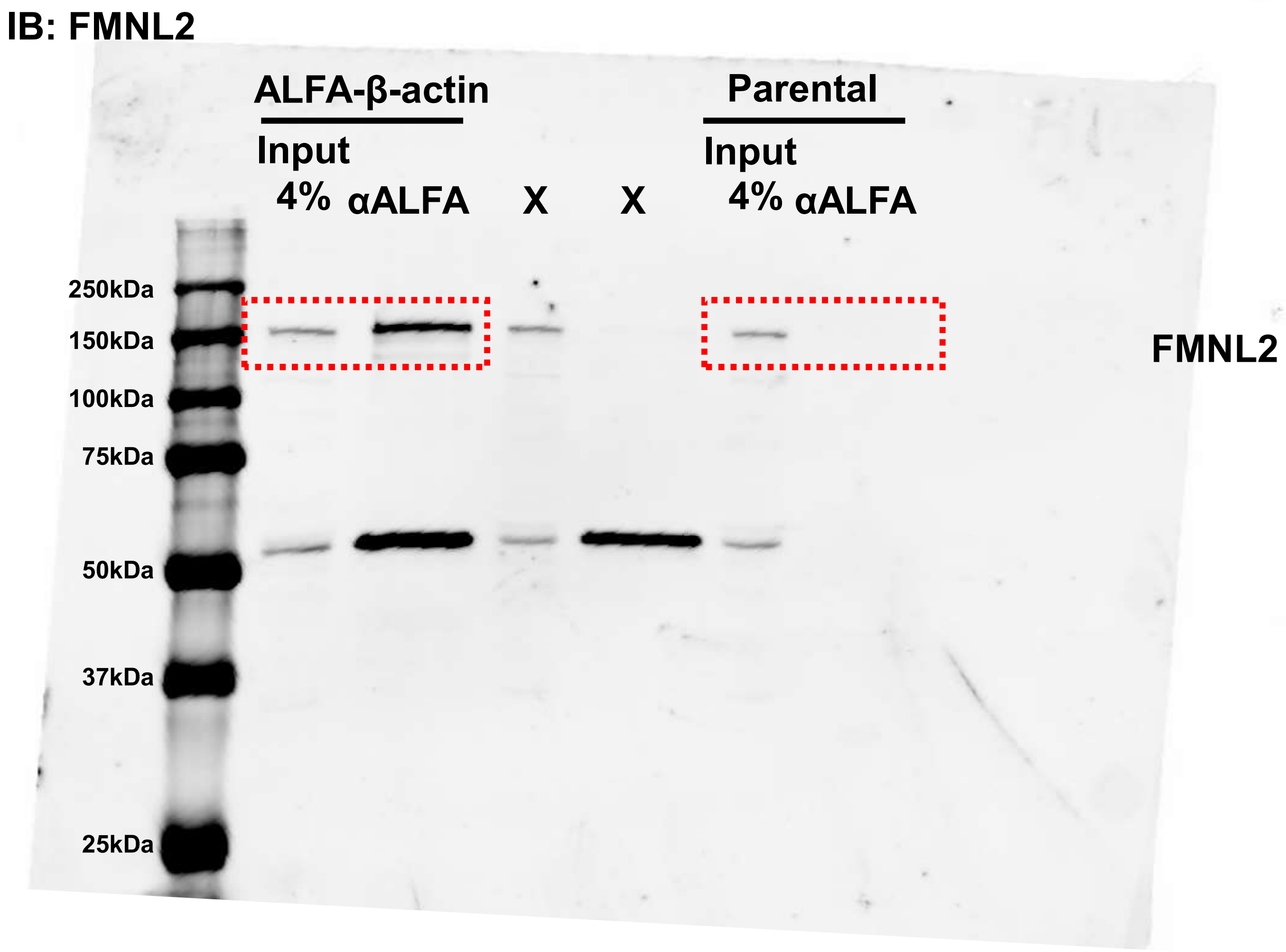
IB: FMNL2
ALFA-β-actin
Parental
Input
4%
αALFA
X
X
Input
4%
αALFA
250kDa
150kDa
100kDa
75kDa
50kDa
37kDa
25kDa
FMNL2

Fig 3D

IB: ALFA

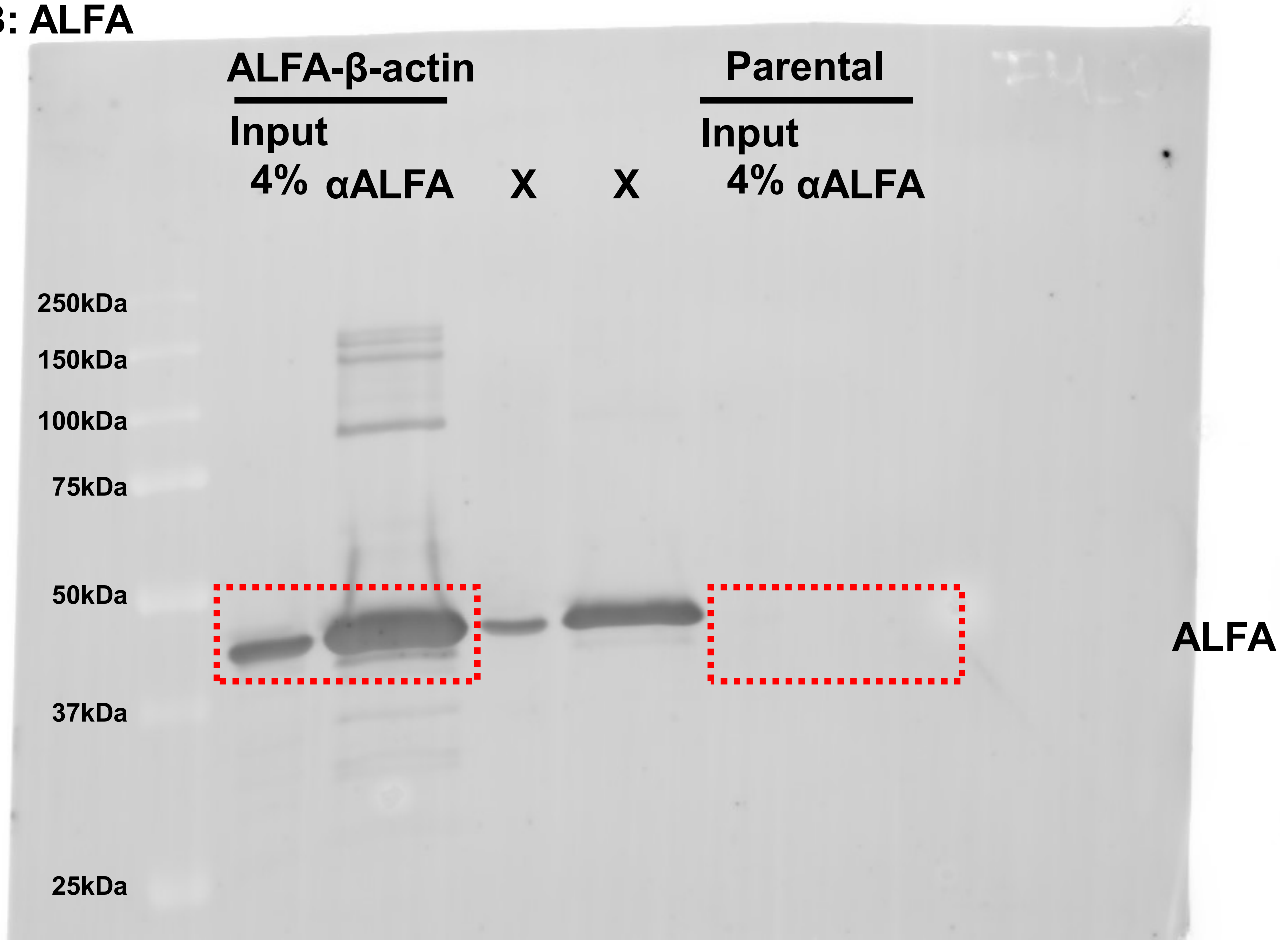

Fig S4C

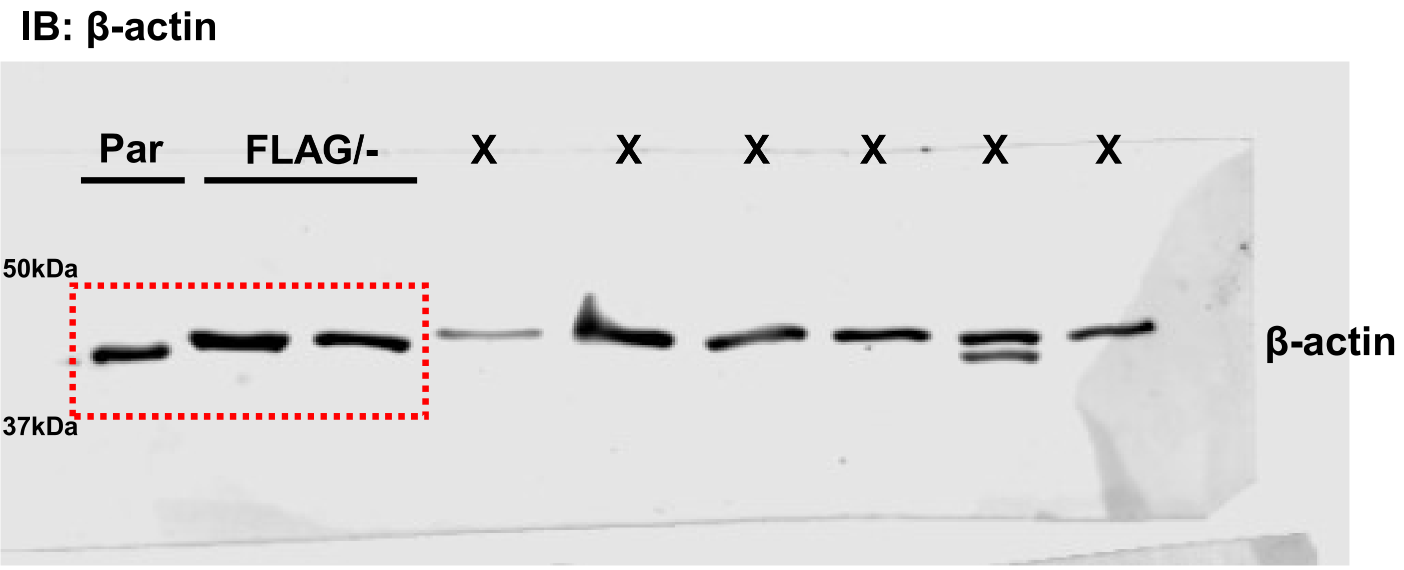

Fig S5B

IB: β-actin and Tubulin

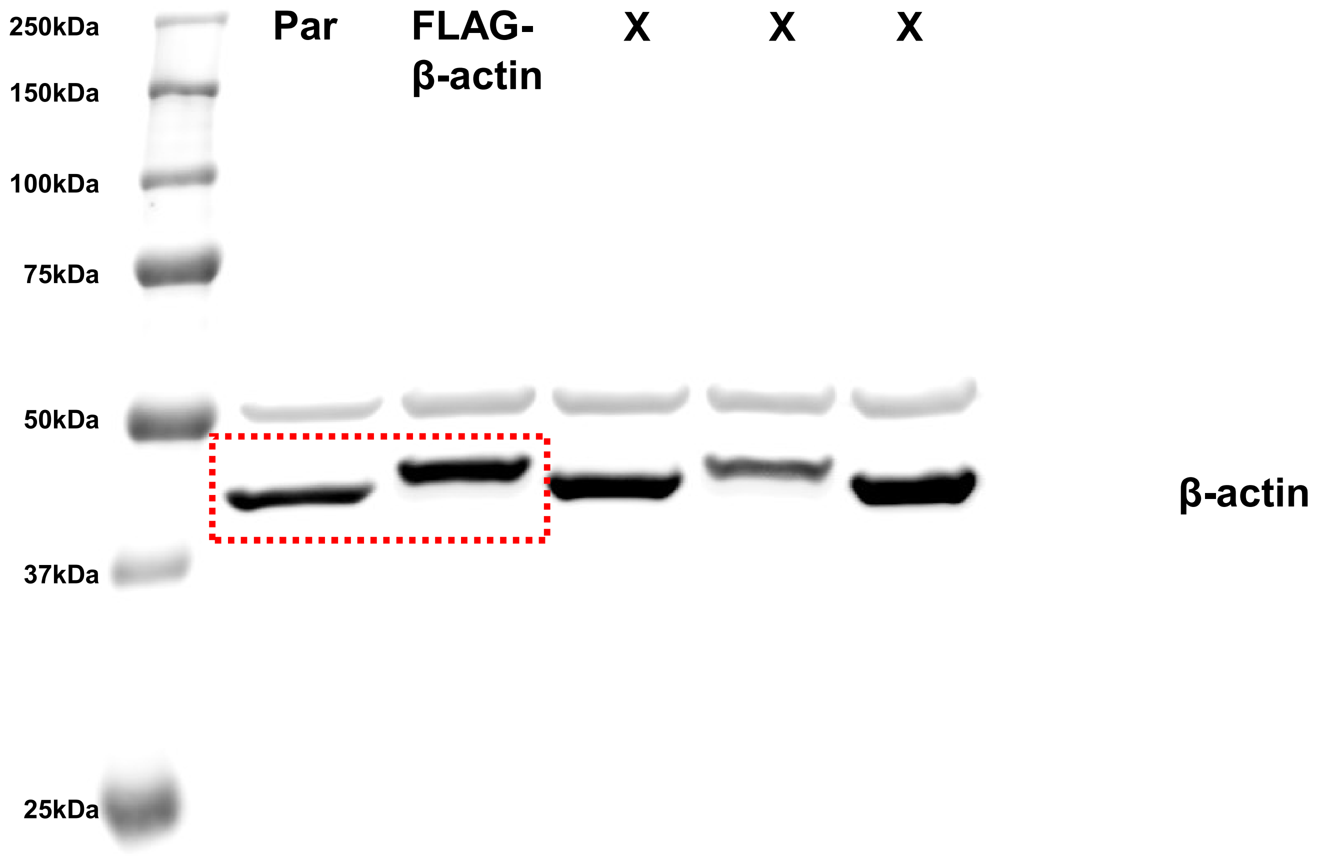

IB: β-actin and Tubulin

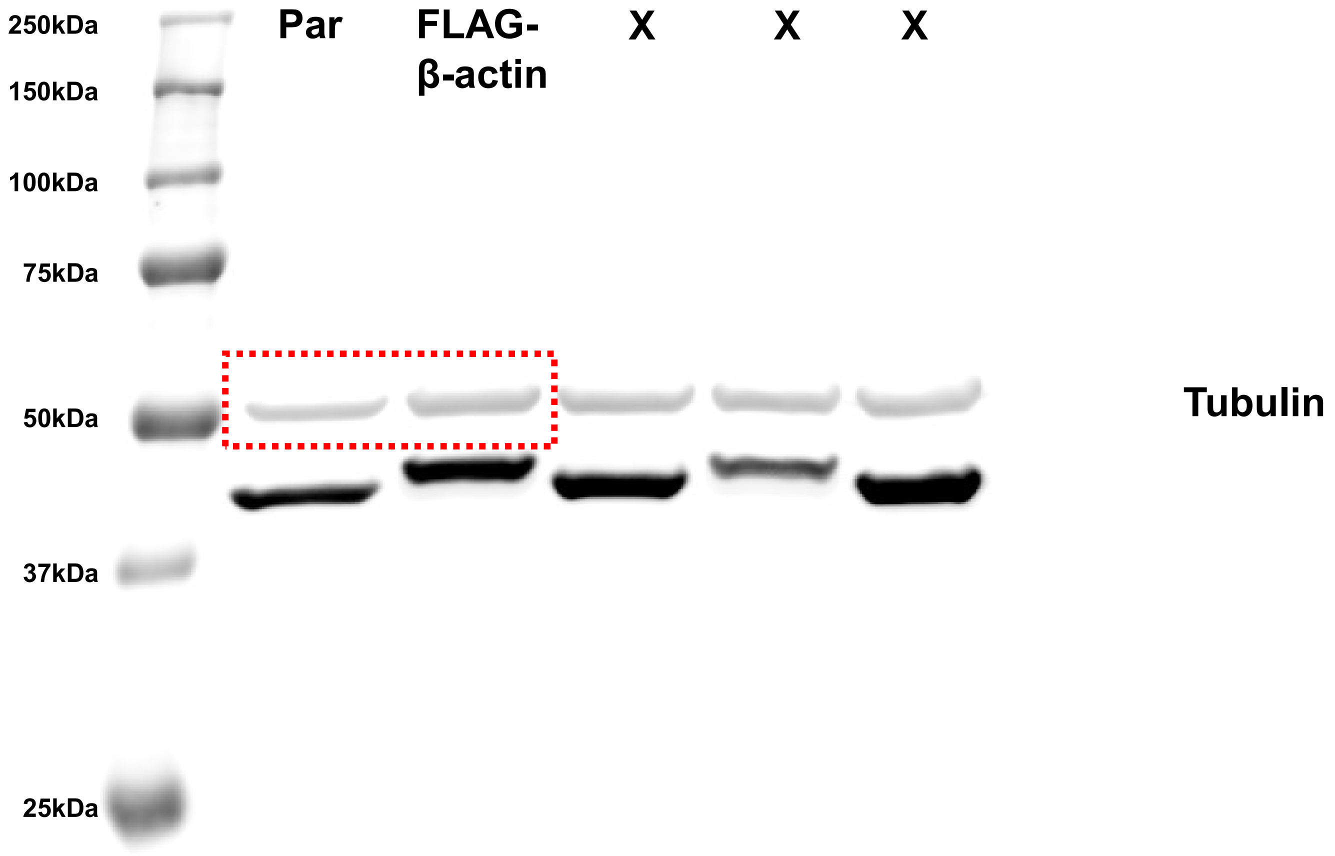

Fig S5B

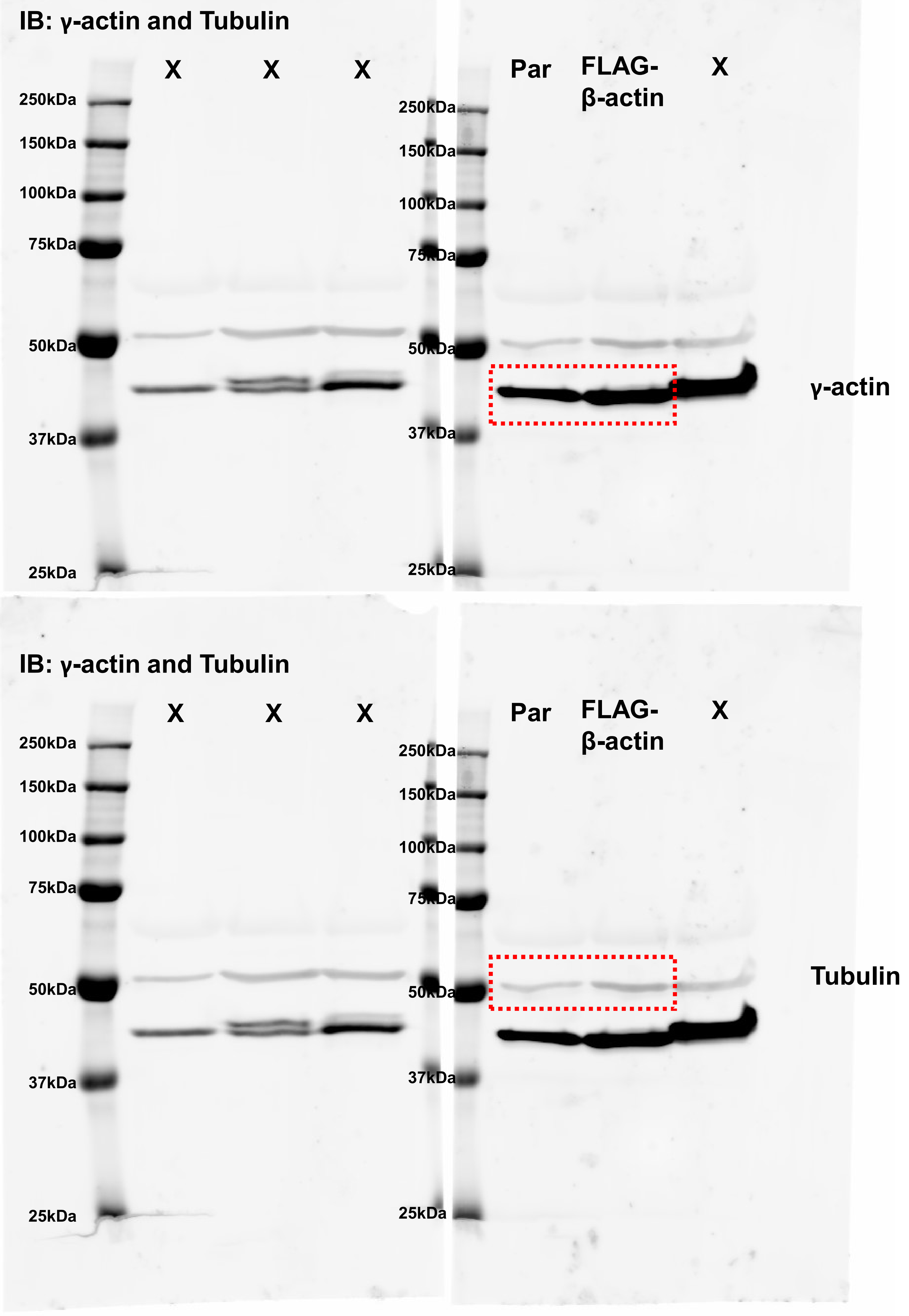
IB: γ-actin and Tubulin
X
X
X
Par
FLAG-β-actin
X
250kDa
150kDa
100kDa
75kDa
50kDa
37kDa
25kDa
γ-actin
IB: γ-actin and Tubulin
X
X
X
Par
FLAG-β-actin
X
Tubulin

Fig S5D

IB: β-actin

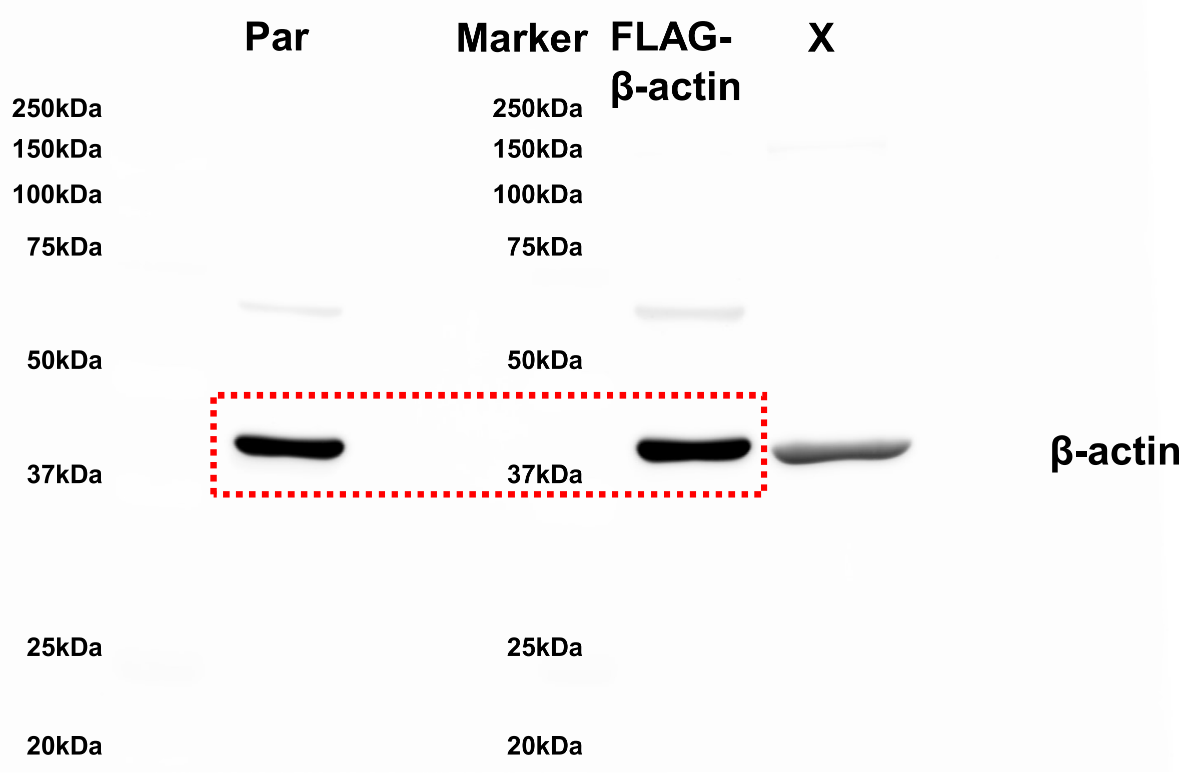

IB: β-actin, adjusted brightness/contrast to show marker

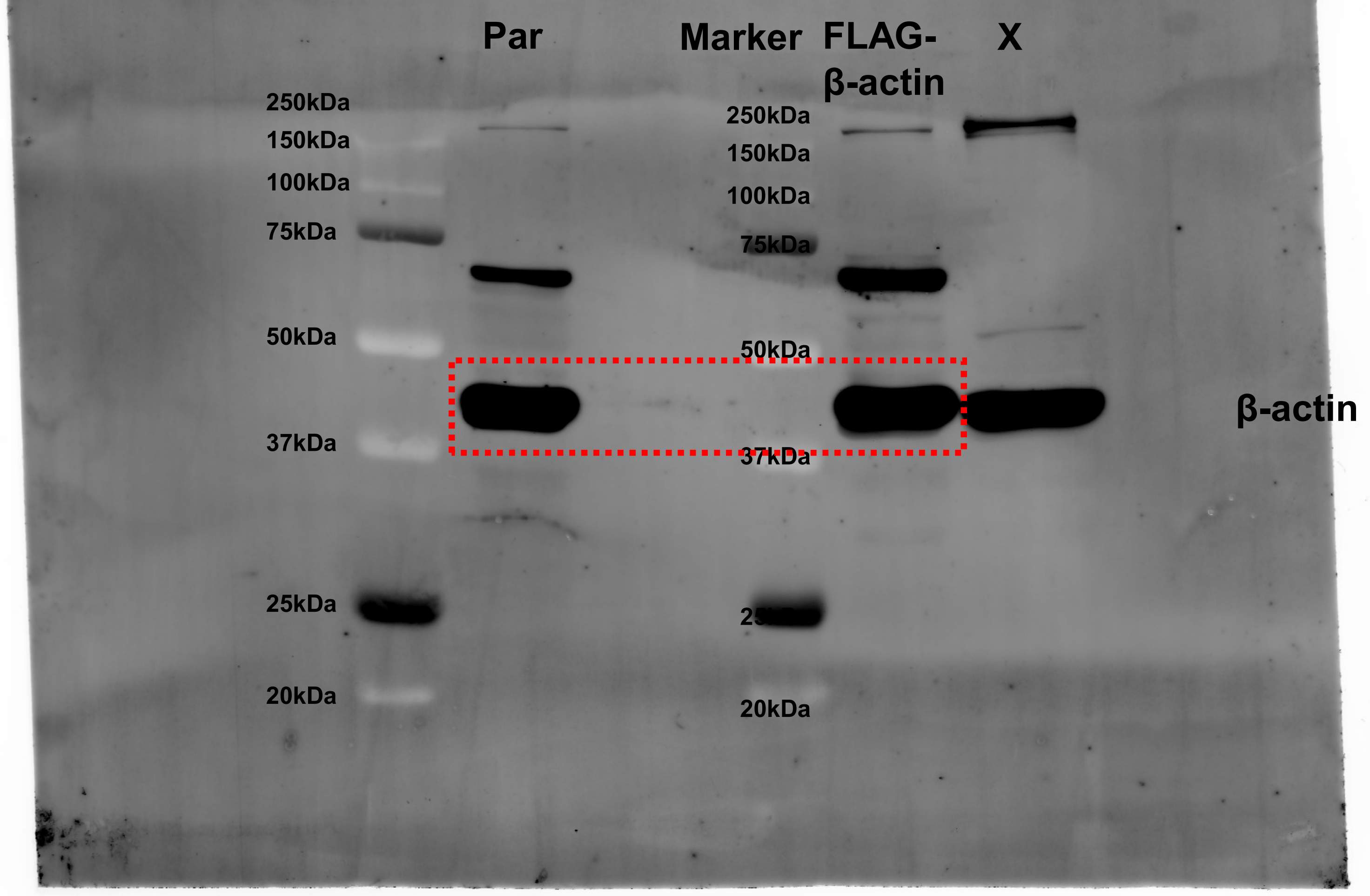

Fig S5D

IB: Tubulin

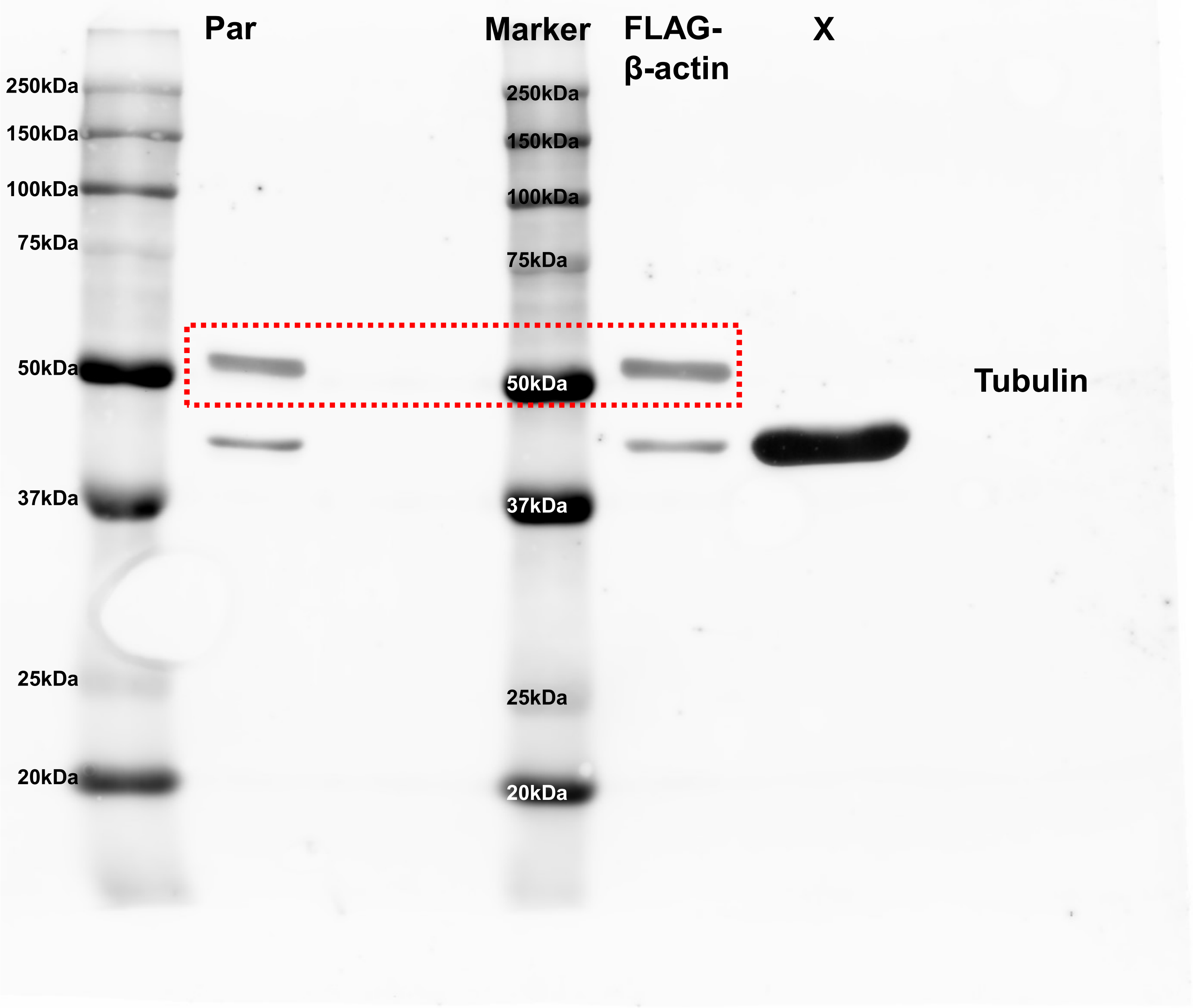

Fig S5F

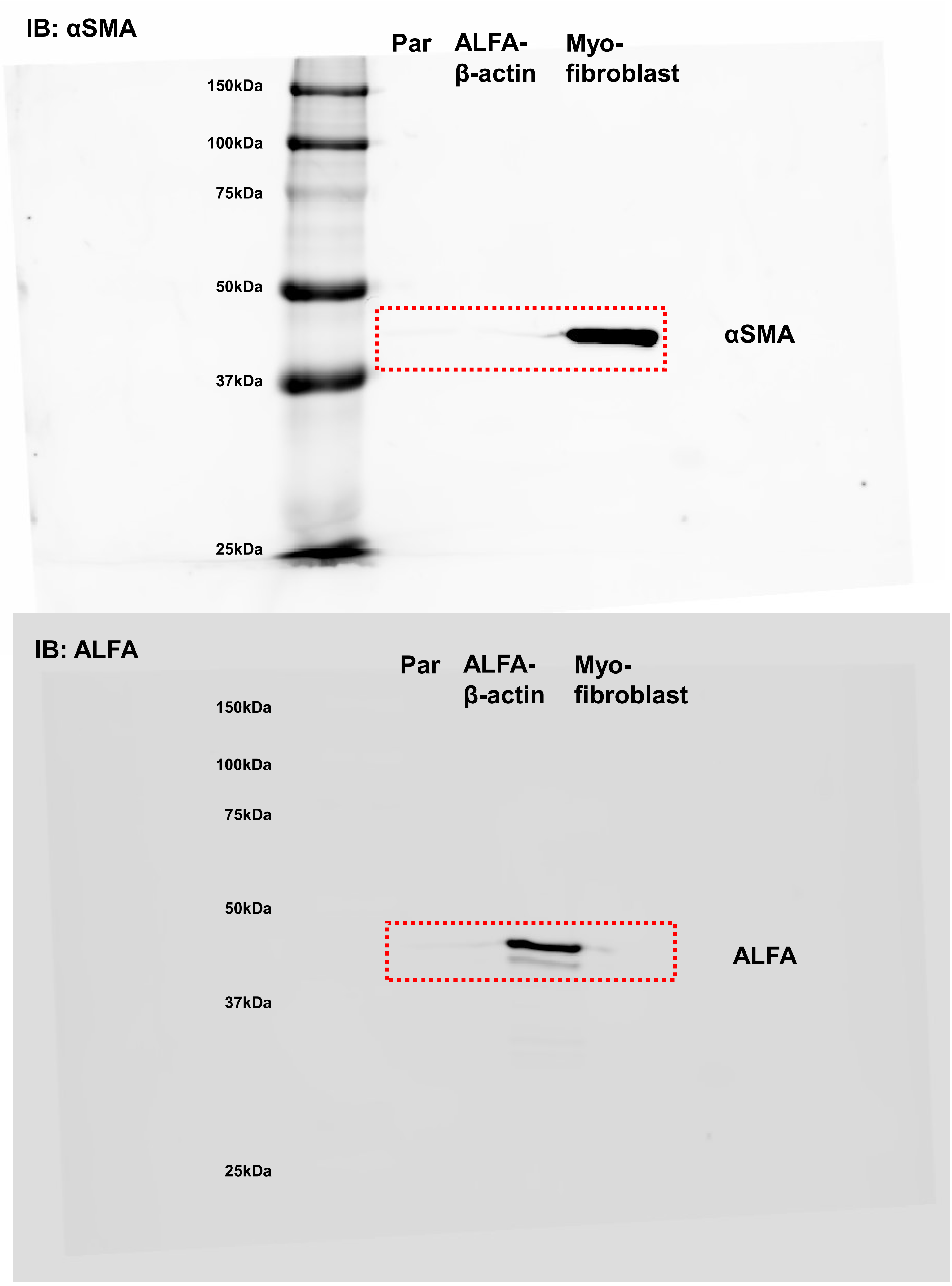
IB: αSMA
Par
ALFA-
β-actin
Myo-
fibroblast
150kDa
100kDa
75kDa
50kDa
37kDa
25kDa
αSMA
IB: ALFA
Par
ALFA-
β-actin
Myo-
fibroblast
150kDa
100kDa
75kDa
50kDa
37kDa
25kDa
ALFA

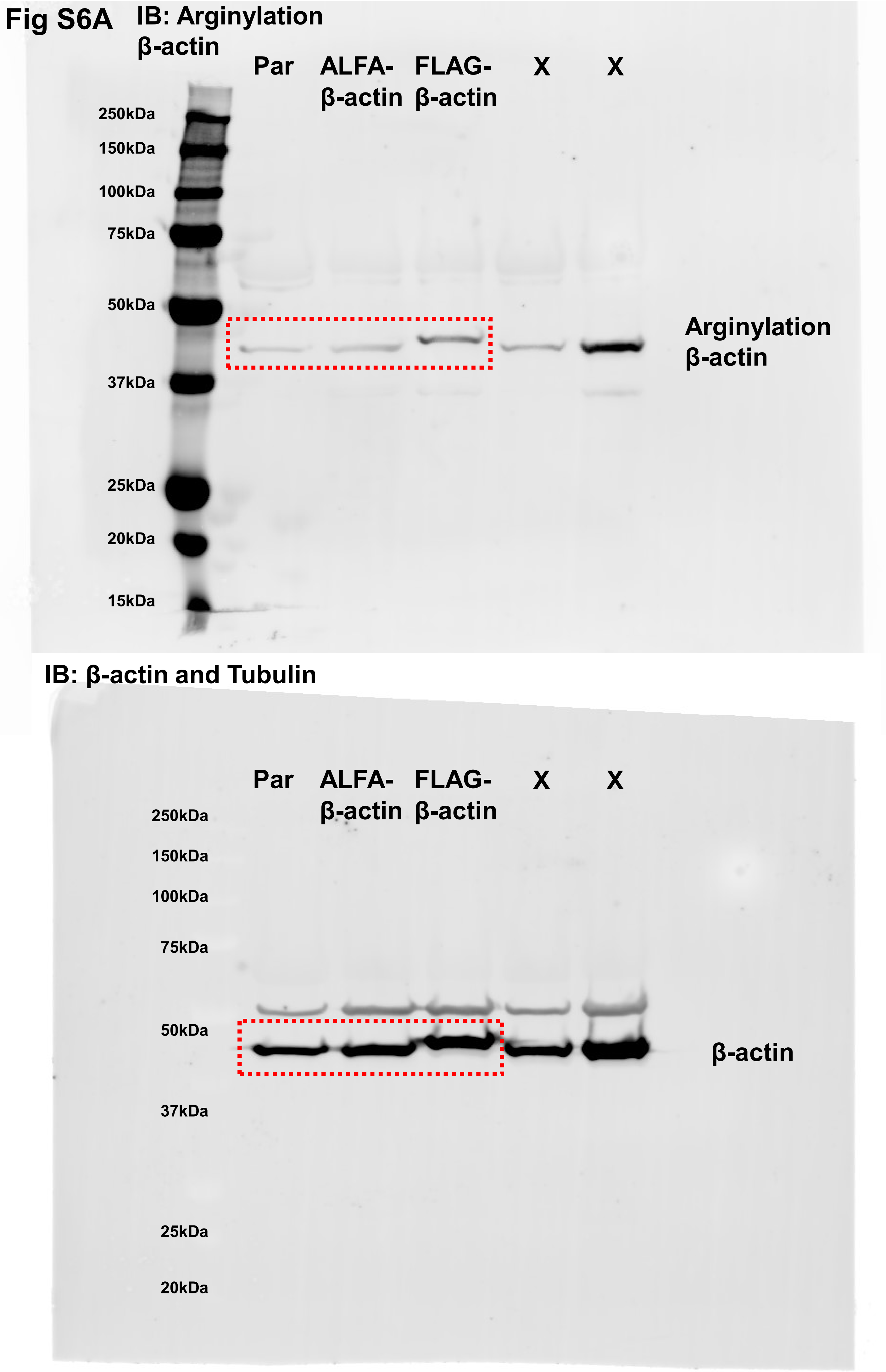
Fig S6A
IB: Arginylation β-actin
Par
ALFA- β-actin
FLAG- β-actin
X
X
250kDa
150kDa
100kDa
75kDa
50kDa
37kDa
25kDa
20kDa
15kDa
Arginylation β-actin
IB: β-actin and Tubulin
Par
ALFA- β-actin
FLAG- β-actin
X
X
250kDa
150kDa
100kDa
75kDa
50kDa
37kDa
25kDa
20kDa
β-actin

Fig S6A

IB: β-actin and Tubulin

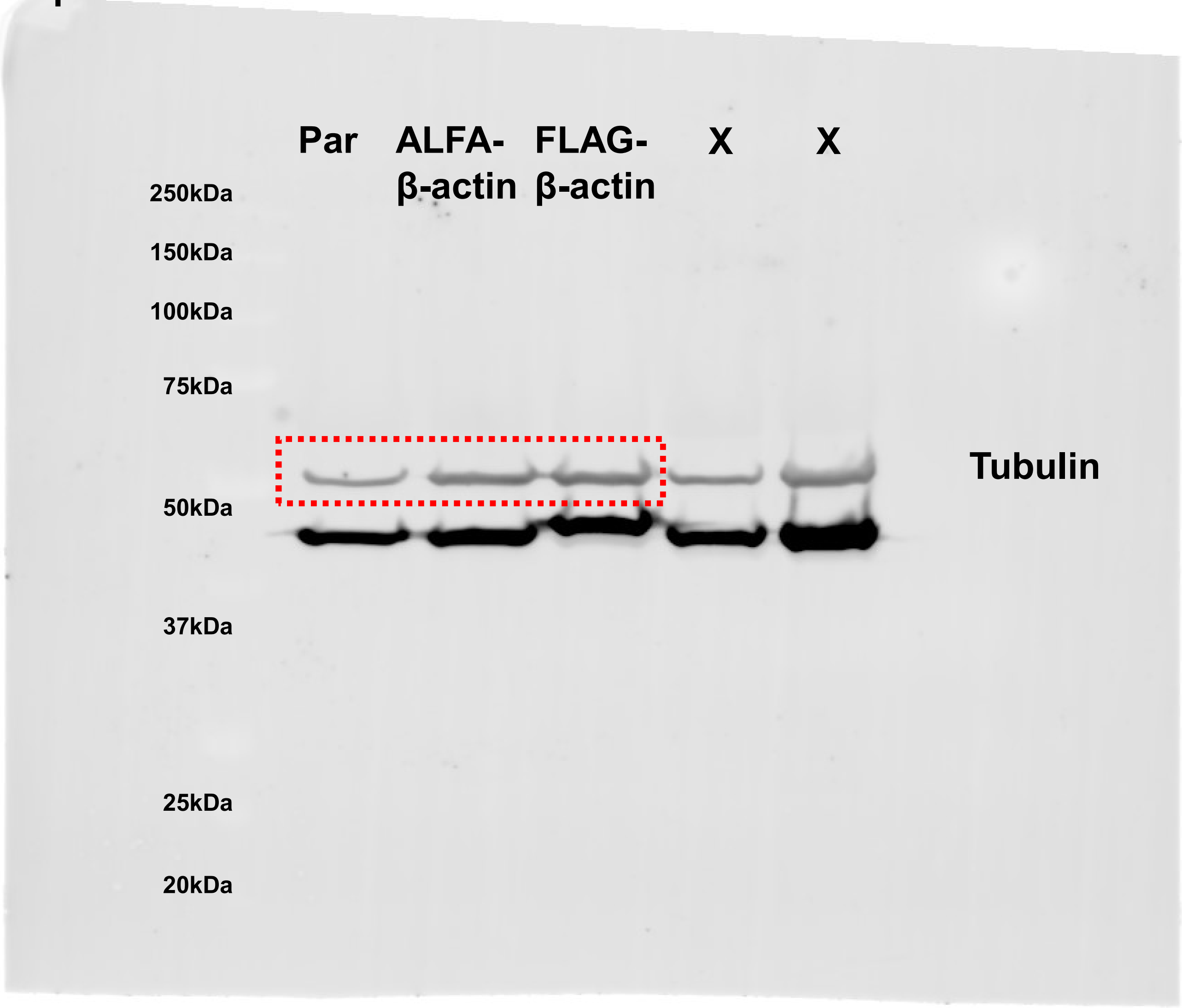

Fig S6B

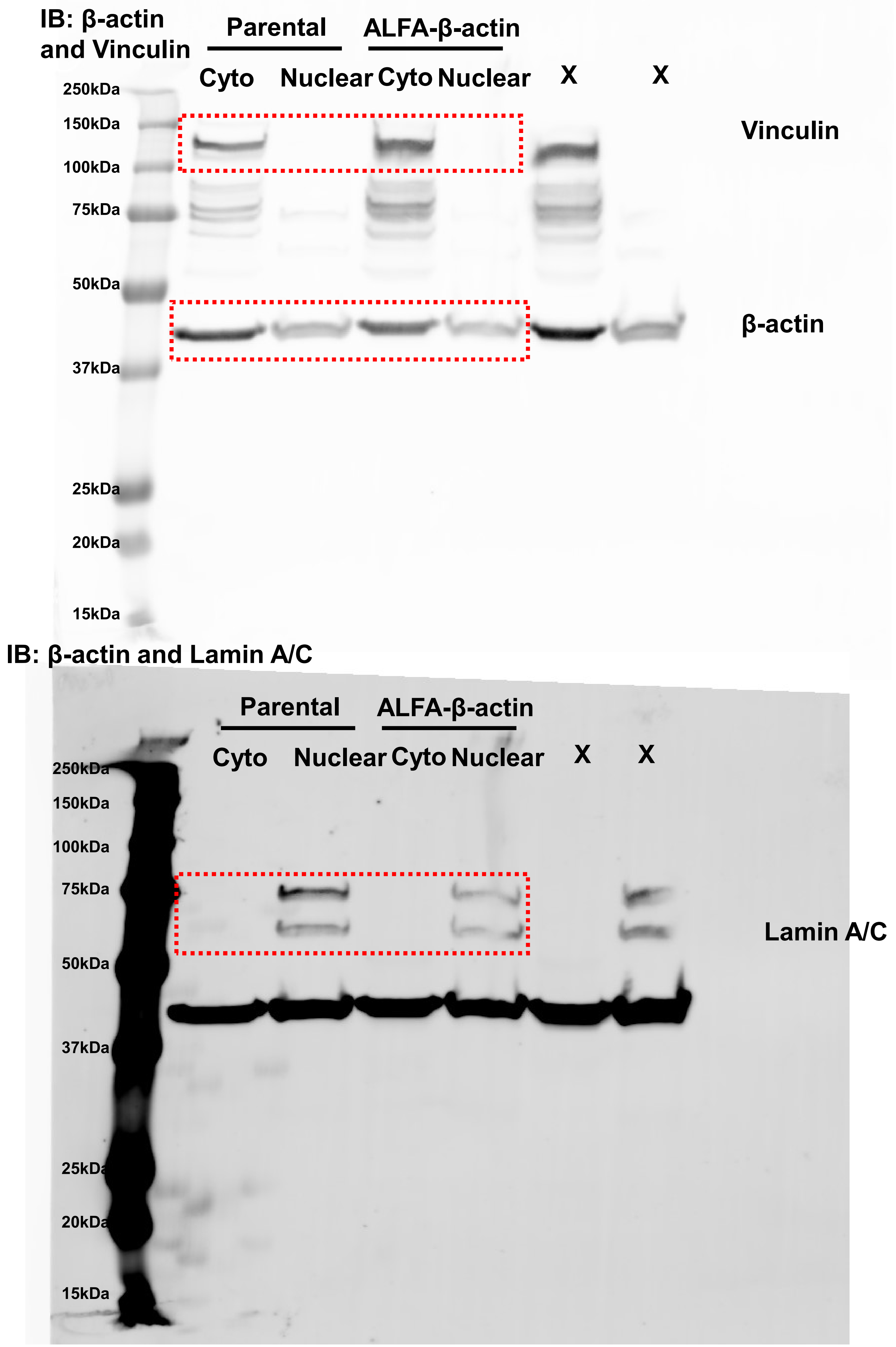
IB: β-actin and Vinculin
Parental
ALFA-β-actin
Cyto
Nuclear
Cyto
Nuclear
X
X
250kDa
150kDa
100kDa
75kDa
50kDa
37kDa
25kDa
20kDa
15kDa
Vinculin
β-actin
IB: β-actin and Lamin A/C
Parental
ALFA-β-actin
Cyto
Nuclear
Cyto
Nuclear
X
X
250kDa
150kDa
100kDa
75kDa
50kDa
37kDa
25kDa
20kDa
15kDa
Lamin A/C

Fig S10A

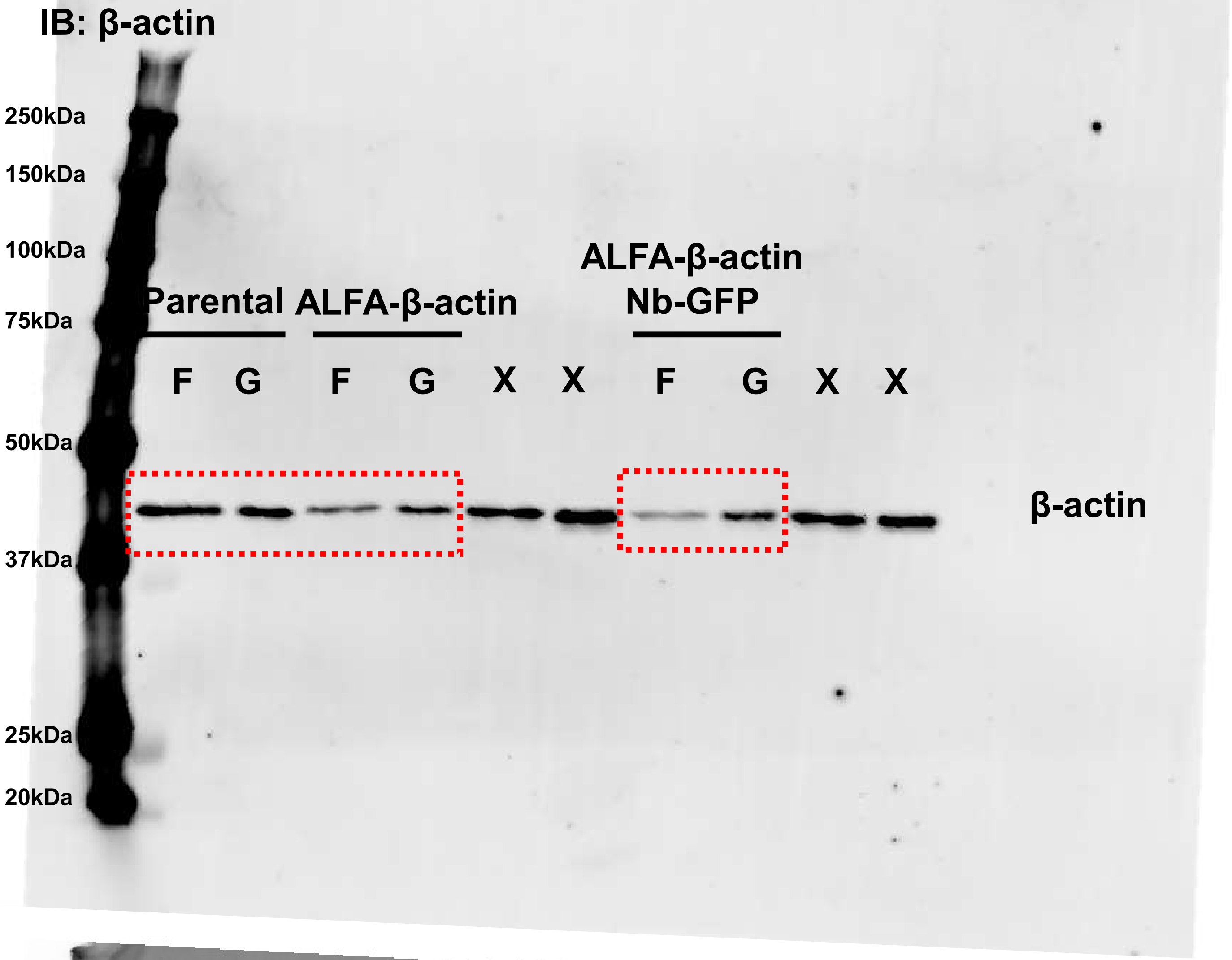
IB: β-actin
250kDa
150kDa
100kDa
75kDa
50kDa
37kDa
25kDa
20kDa
Parental
ALFA-β-actin
ALFA-β-actin
Nb-GFP
F G F G X X F G X X
β-actin

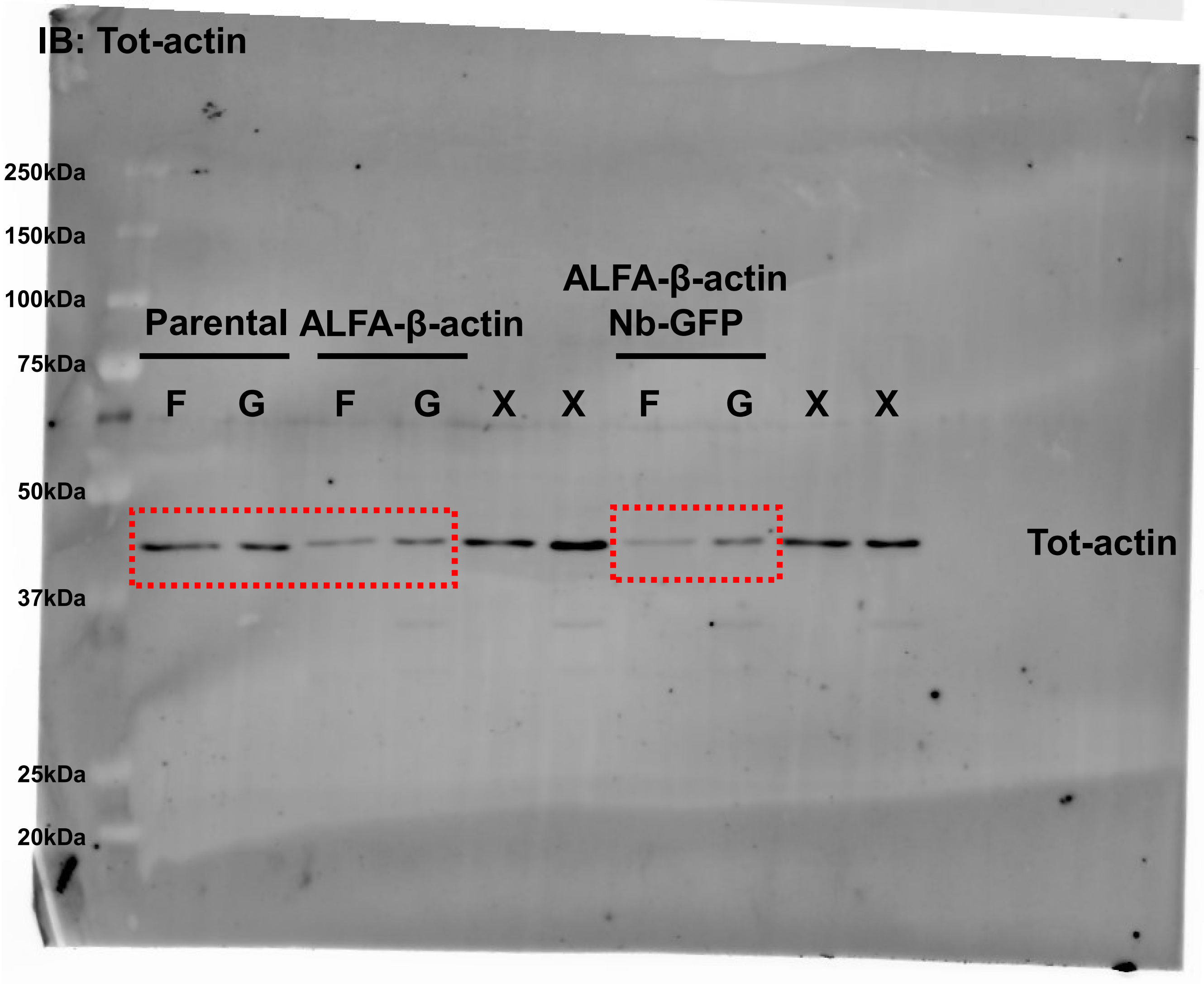
IB: Tot-actin
250kDa
150kDa
100kDa
75kDa
50kDa
37kDa
25kDa
20kDa
Parental
ALFA-β-actin
ALFA-β-actin
Nb-GFP
F G F G X X F G X X
Tot-actin

Fig S11A

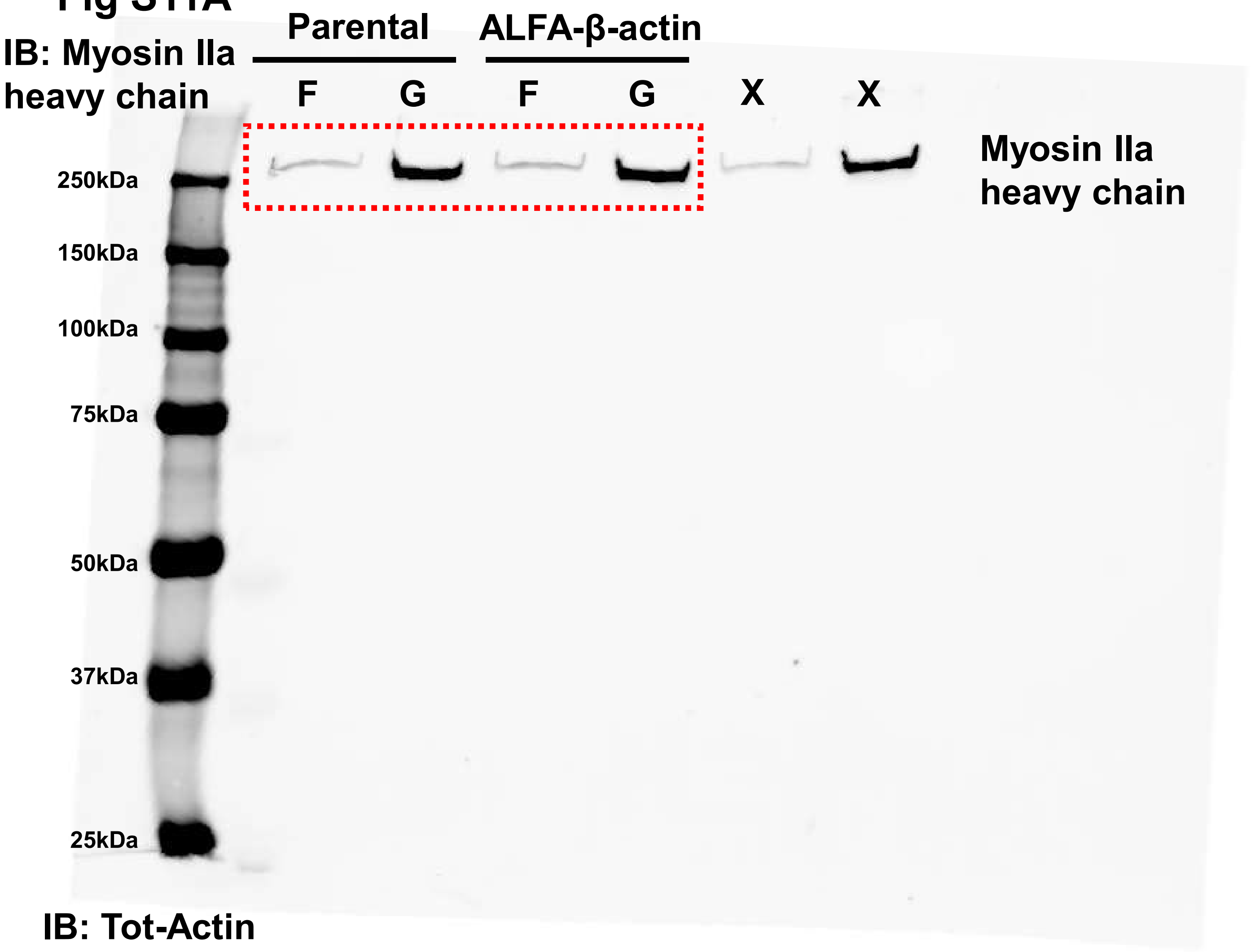
IB: Myosin IIa heavy chain
Parental
ALFA-β-actin
F
G
F
G
X
X
250kDa
150kDa
100kDa
75kDa
50kDa
37kDa
25kDa
Myosin IIa heavy chain

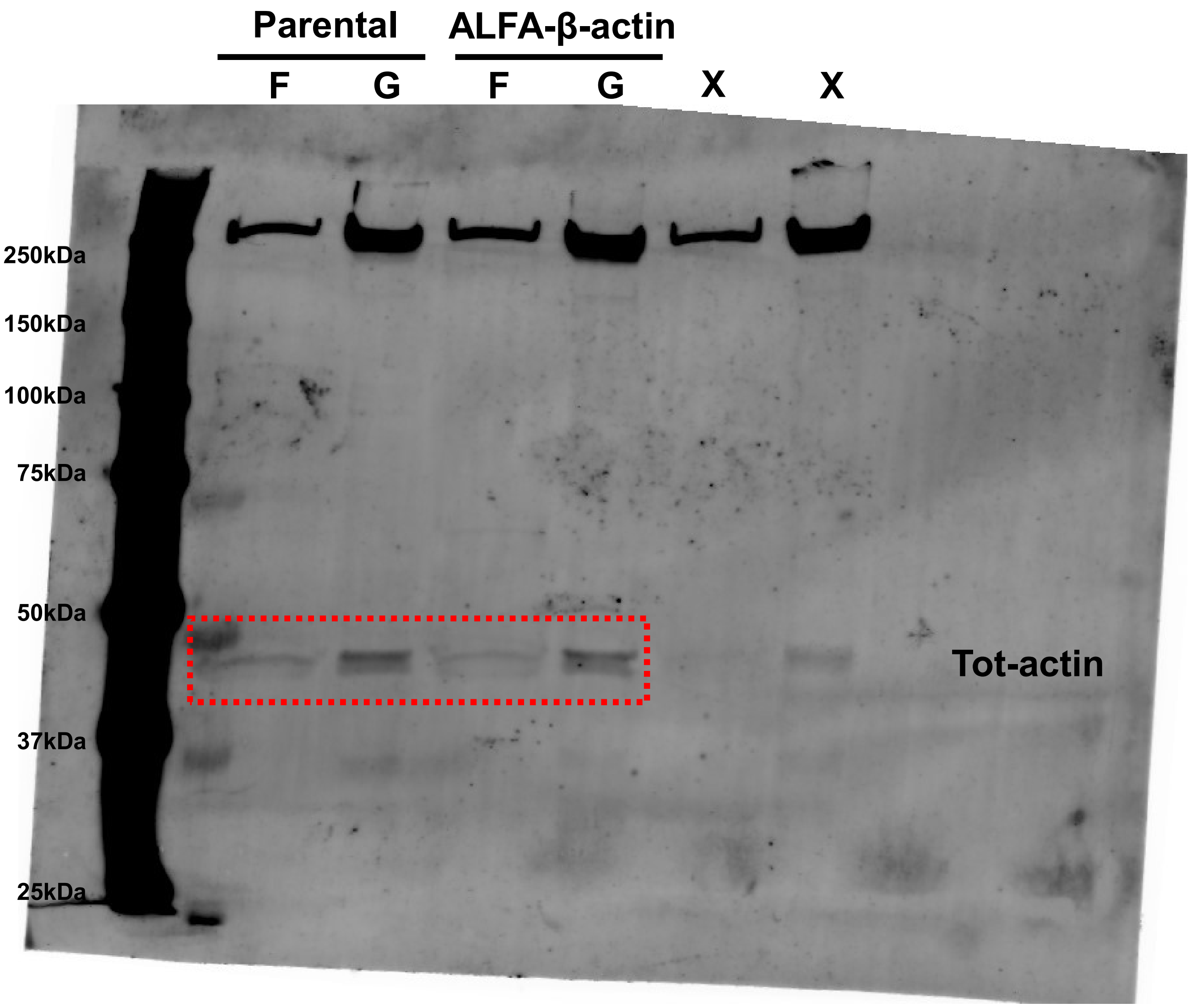
IB: Tot-Actin
Parental
ALFA-β-actin
F
G
F
G
X
X
250kDa
150kDa
100kDa
75kDa
50kDa
37kDa
25kDa
Tot-actin

Fig S11B

IB: α-actinin

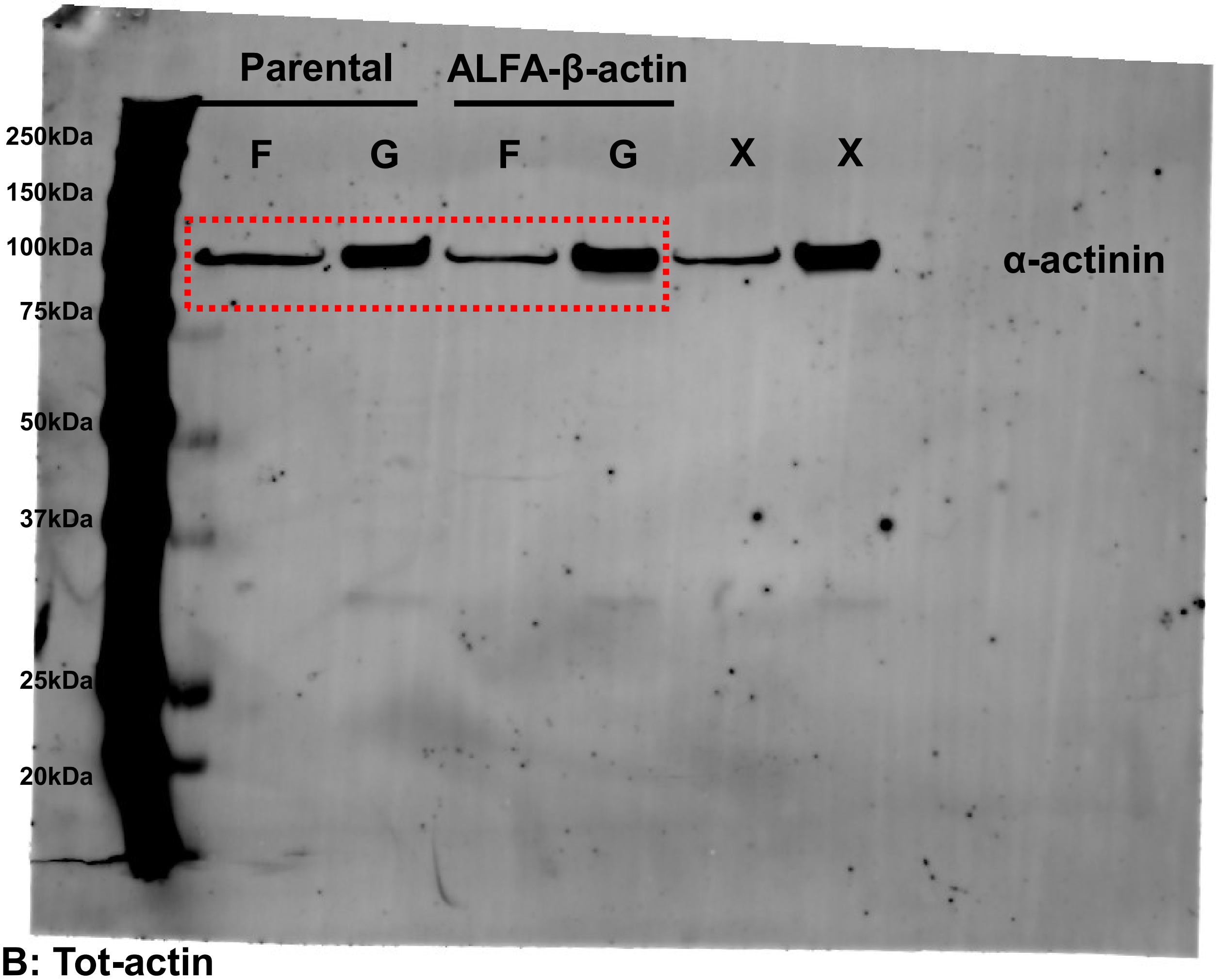

IB: Tot-actin

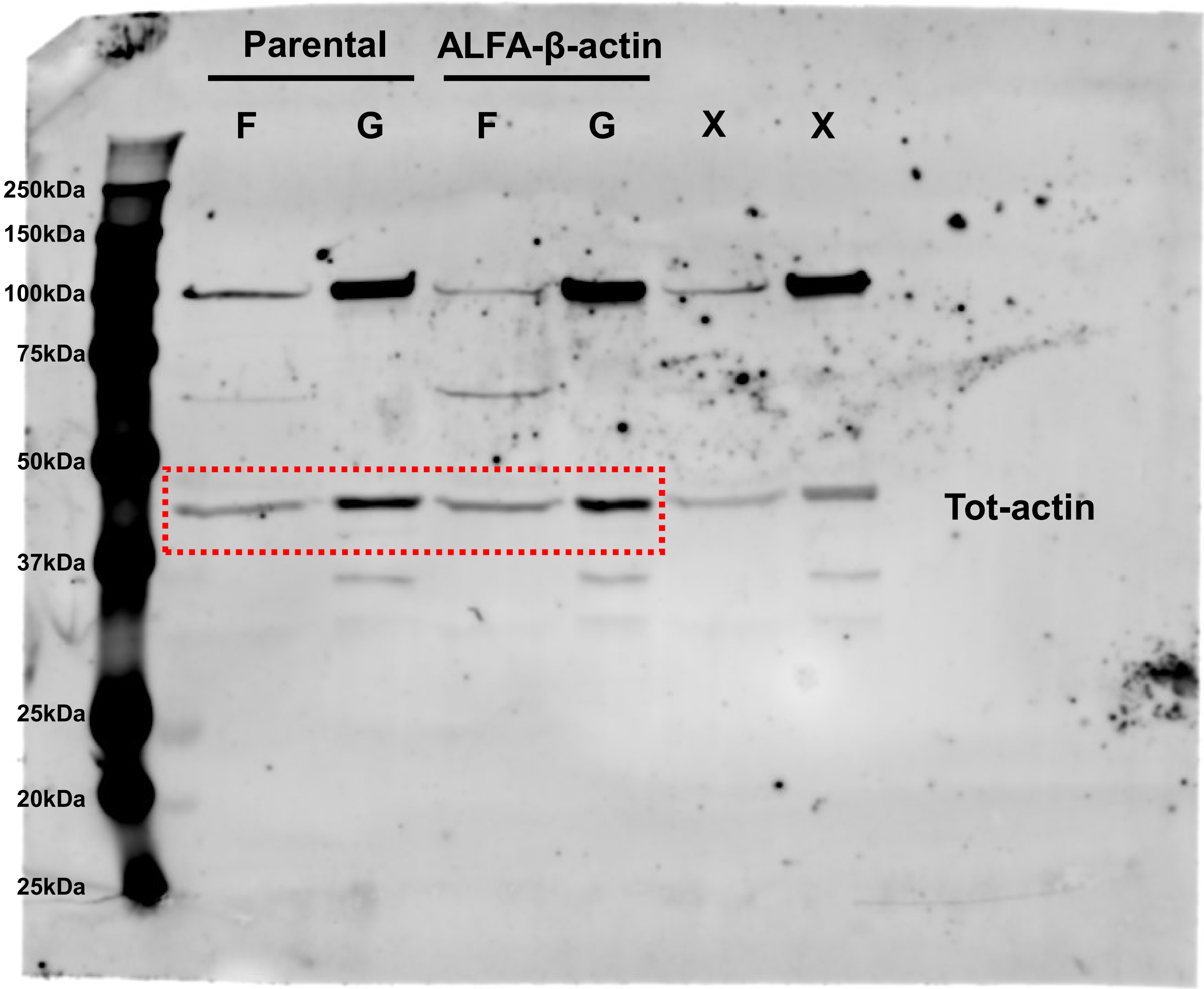

Fig S16B

IB: S.cerivisiae actin (ACT1)

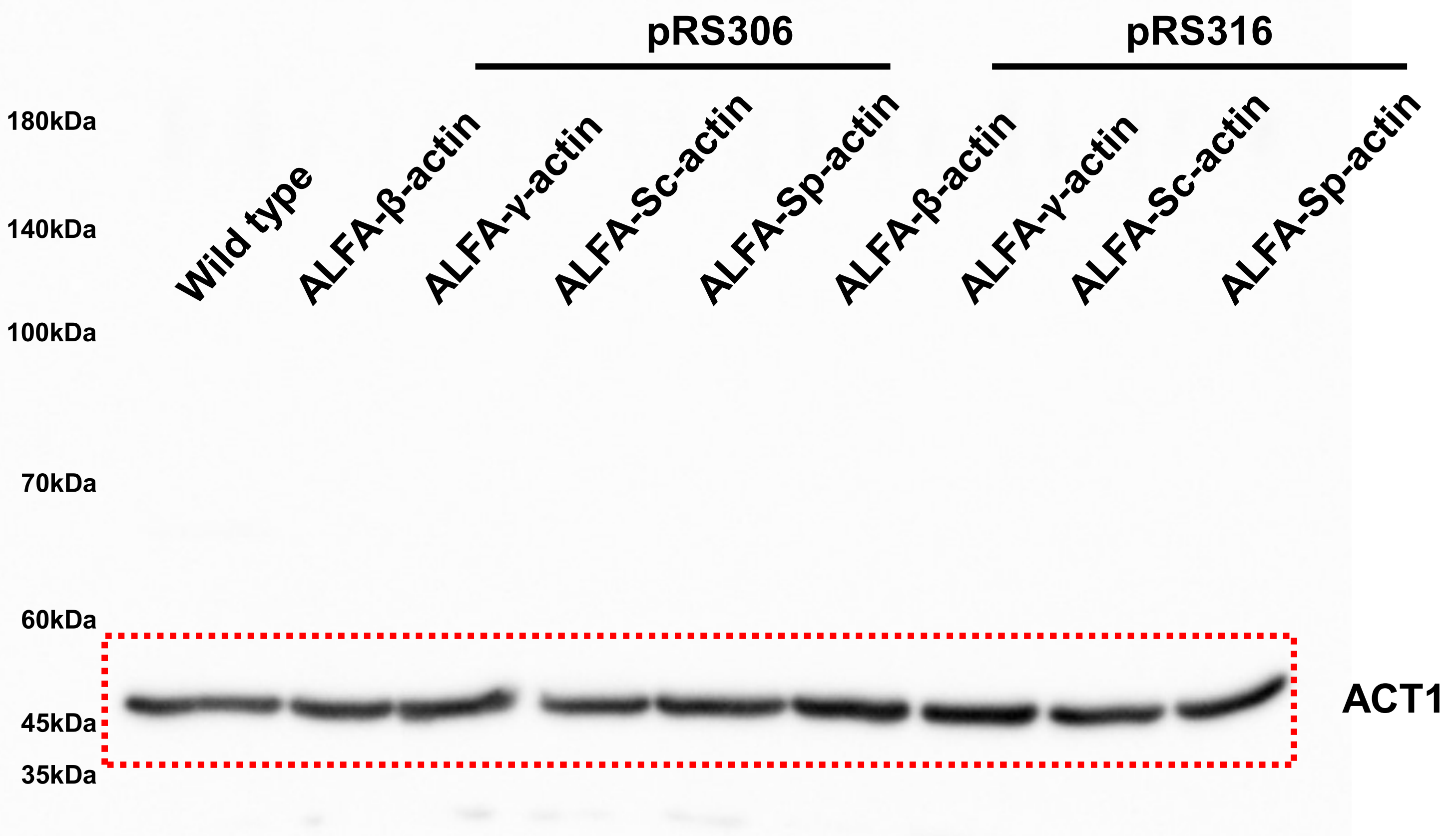

IB: S.cerivisiae actin (ACT1) picture of membrane to show marker

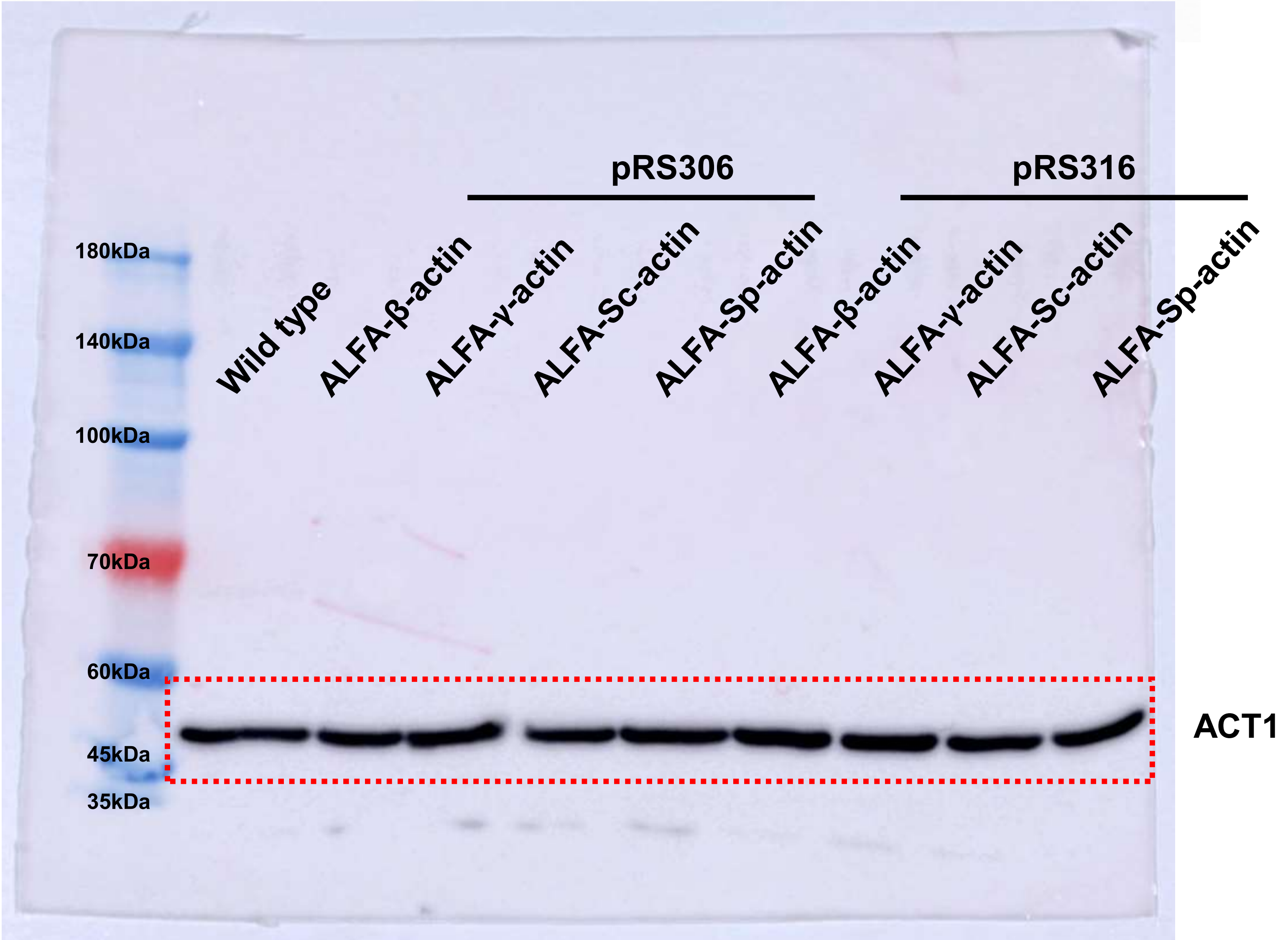

Fig S16B

IB: ALFA

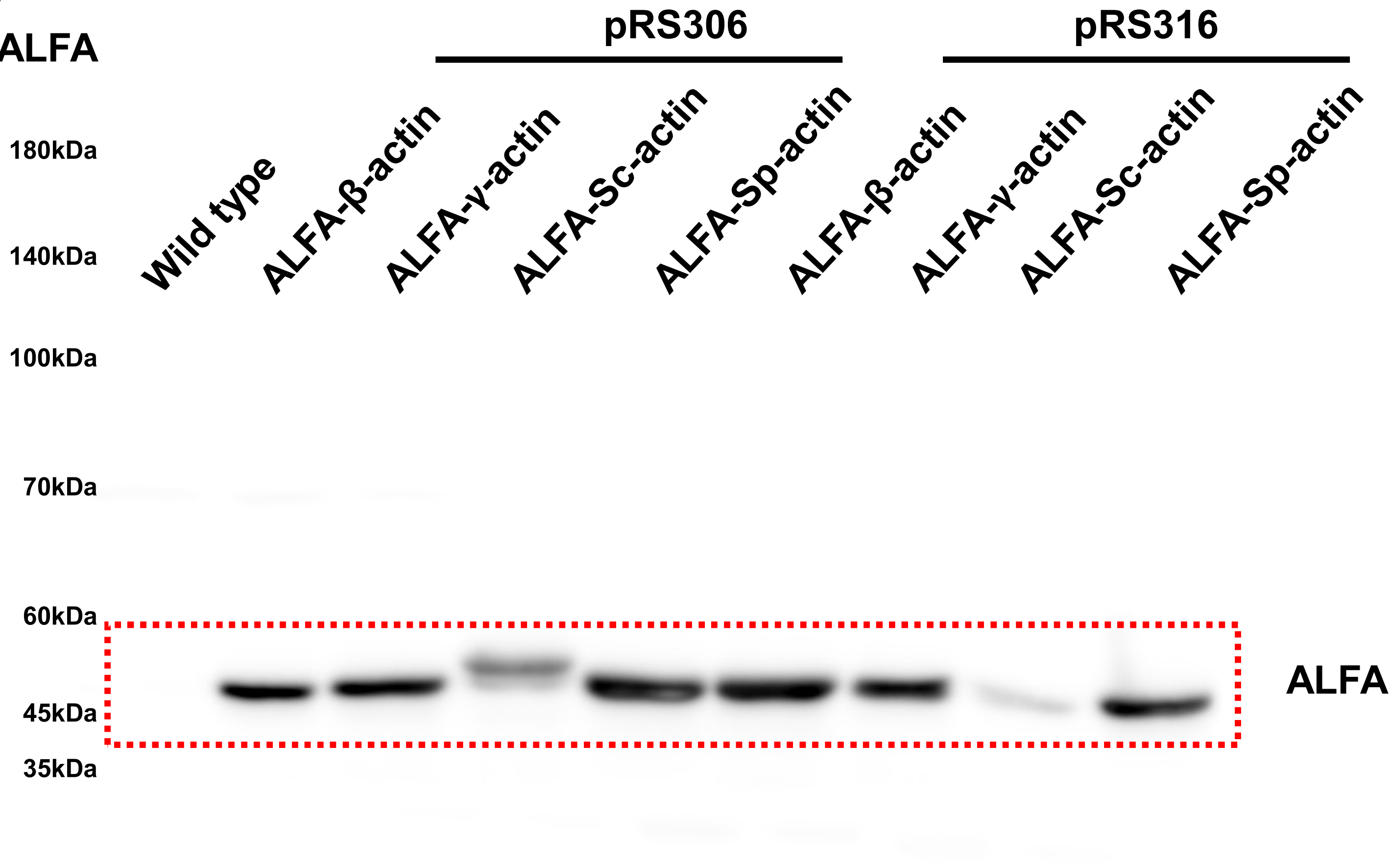

IB: ALFA picture of membrane to show marker

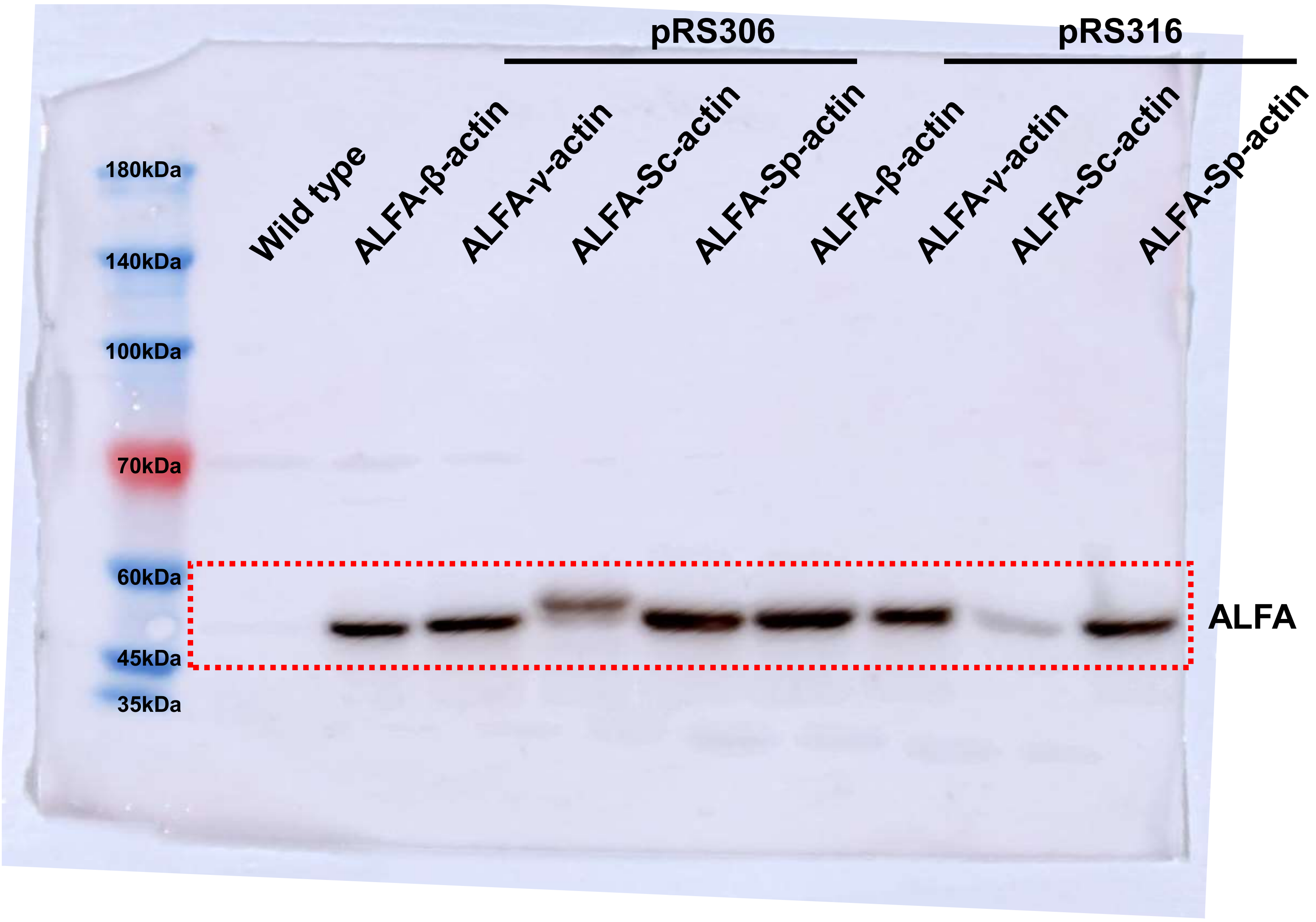

Supplement: S1 Raw Images — The data are organized into separate pages corresponding to the following figure panels: 2A, 2C, 2G, 3A, 3B, 3C, 3D, S4C, S5B, S5D, S5F, S6A, S6B, S10A, S11A, S11B, and S16B. (PDF) [file pbio.3002551.s033.pdf]
