# Supplementary material for: IntAct: A nondisruptive internal tagging strategy to study the organization and function of actin isoforms
Source: PLoS Biol. 2024 Mar 11;22(3):e3002551. doi: 10.1371/journal.pbio.3002551 (PMC10957077; doi:10.1371/journal.pbio.3002551)
Supplement: Attachment — Submitted filename: 137422_1_rebuttal_2781643_s0bstz.pdf [file pbio.3002551.s034.pdf]

Revision Plan

Manuscript number: RC-2023-02049
Corresponding author(s): van den Dries, Koen

1. General Statements

We thank the Referees for their constructive and positive comments on our manuscript. We were happy to see that all three referees acknowledge the general and broad significance of our novel actin tagging approach and its potential for unravelling unknown aspects of actin biology. The Referees have suggested several experiments to further determine how the functionality of the tagged actins compares to wildtype actin. We plan to include the majority of these experiments in our revised manuscript. In fact, we have already performed some of the suggested experiments, for which we include the results below. To avoid confusion, we have not included these additional experiments yet into the submitted manuscript.

2. Description of the planned revisions

In response to Reviewer #1:

In this study, the authors generate several variants of actin that are internally tagged with short peptide tags. They identify one particular position that is able to tolerate various tags of 5-10 amino acids and still shows largely unaltered behavior in cells. They study incorporation of their tagged actins into filaments, characterize the interactions of G-actin variants with different associated proteins and show that retrograde actin flow in lamellipodia and the wound healing response of epithelial cells is not affected by the tagged variants. They then apply the tagged actin to study subcellular distribution of different actin isoforms in mammalian and yeast cells.

The identification of a specific site in the actin protein that tolerates variable peptide insertions is very exciting and of fundamental interest for all research fields that deal with cytoskeletal rearrangements and cellular morphogenesis. The result demonstrating the functionality of actin variants with peptides inserted between aa 229 and 230 are generally convincing and well done. In particular, the generation of CRISPR/Cas9 genome edited versions of beta- and gamma actin are impressive. I therefore generally support publication of this study. There are however several technical and conceptual issues that should be addressed to improve quality and scope of the study. I listed some specific comments below:

We thank the Reviewer for their constructive comments and general support for publication of our study.

Major points

- The biggest issue I have is the last section on the application of tagged actins to study isoform functions. In principle the application is very clear as there are simply no alternative ways to study isoform distribution in live cells. However, the experimental data are simply not convincing. What the authors define as "cortex" in Fig. 5A seems to rather represent cytosolic background mixed with radial fibers. I am not convinced that even the antibody staining with a relatively clear differential distribution of beta and gamma really shows a genuine accumulation of one isoform on stress fibers. It seems to me that the beta-actin staining has as higher cytosolic background and is generally weaker (gamma nicely labels transverse arcs), which reduces signal/noise and therefore yields a relatively increased level in areas with less-bundled actin. My suggestion is to select more clearly defined actin structures and to use micro-patterned cells to normalize the

otherwise obstructing variability in actin organization. Possible structures would be cortical arcs in bow-shaped cells, lamellipodial edges (HT1080 seem to make very nice and large lamellipodia) or cell-cell contacts (confluent monolayer, provided cells don´t grow on top of each other). Stress fibers are possible but need to be segmented very precisely and I did not see any details on this in the methods section. For Fig. 5D: I assume cells were used where only one isoform was tagged? This is technical weak and the double-normalization is probably blurring any difference that might be occurring. Why not use a double-tagging strategy with ALFA/FLAG or ALFA/AU5 tags to exploit the constructs introduced in the previous figures? Also, the unique selling point of the strategy is the possibility of actual live imaging of specific isoforms. Cells that have stably integrated double tags and then transiently express nanobodies for ALFA and either AU5 or FLAG (or other if those don't exist) would make this possible. Considering the work already done in this manuscript, such an approach should actually be possible - did the authors attempt this or is there are reason it is not discussed? If double tagged cells are not possible for some reason it should at the very least be possible to combine ALFA-detection with the specific antibody against the other isoform and get rid of the double normalization.

The Reviewer presents various suggestions regarding the comparison between the localization of the tagged and native isoforms. In our response, we will separately discuss the possibilities and our considerations for fixed samples and live-cell imaging. The response for the fixed samples can be found directly below. For the live-cell imaging, we kindly refer to the section "*Description of analyses that authors prefer not to carry out*".

We apologize for the lengthy response but for transparency reasons, we would like to give a thorough overview of our efforts for isoform-specific localization in cells, something for which we have limited space in the manuscript.

Fixed samples:

It was a significant experimental challenge to comparing the labeling of the β- and γ-actin specific antibodies with our internally tagged actin system (**Fig. 5A-D**). The reason for this is that the labeling of the samples with the β- and γ-actin specific antibodies requires treatment with methanol (*Dugina et al., J Cell Sci, 2009*), most likely to disturb the interaction of actin with actin-binding proteins that prevent the binding of the antibodies due to steric hindrance. Methanol treatment, however, precludes the co-labeling with phalloidin, likely due to changes in the tertiary/quaternary protein structure of F-actin. Initially, we have put a lot of effort in trying to simultaneously label phalloidin with the actin specific antibodies but even very brief methanol treatment (seconds), before or after phalloidin labeling, completely prevents/reverses the binding of phalloidin. Importantly, also the ALFA tag labeling was suboptimal after methanol treatment.

The fact that we could not perform these double labelings led us to perform different ratio calculations for the β- and γ-actin antibody and the ALFA tag labeling. In the case of the antibody immunofluorescence labeling, we simply divided the signal of the β-actin and γ-actin since we could simultaneously label the isoforms in the same cell. In the case of the ALFA tag labeling, we used phalloidin for independent signal normalization and then performed a second normalization. Although this complicates the normalization procedure (ALFA tag signal of β- and γ-actin is first normalized to total F-actin and then a ratio is calculated) and understandably leads to some confusion, this was the only way forward to obtain the results presented in the manuscript.

The Reviewer points out that "*What the authors define as "cortex" in Fig. 5A seems to rather represent cytosolic background mixed with radial fibers.*". In our images, we observe very little

cytosolic background from both antibody stainings. More importantly, for the quantitative analysis, the fluorescence intensity values were corrected for the background values observed in cytosolic areas so even if the signal is present, it should not affect our analysis. We do admit though that we could have been more careful with the term "cortex" since the observed signal could indeed be a mix of radial fibers and the actin cortex. The reviewer further states that "*I am not convinced that even the antibody staining with a relatively clear differential distribution of beta and gamma really shows a genuine accumulation of one isoform on stress fibers.*" Although the differences are small, we consistently observe a differential fluorescence intensity of β- and γ-actin in actin-based structures with a relatively stronger signal of γ-actin in stress fibers (**Fig. 5C**). Since we always normalize the fluorescent signal intensity per cell, this strongly indicates a genuine accumulation of one isoform over the other in specific actin-based structures. This observation is very consistent in our experiments and also aligns with many published studies where differences in the localization of β- and γ-actin are reported in various cell types (Pasquier et al., Vasc Cell, 2015; van den Dries et al., Nat Comms, 2019; Malek et al., Int J Mol Sci, 2020). As for the segmentation, we mentioned in the Methods section that we selected small regions (0.5x0.5μm) that exclusively contain stress fiber or "cortex" regions. The regions shown in **Fig. 5B** are therefore larger than the analyzed regions, something which we will better indicate in the revised manuscript.

Planned revision: We will provide a more detailed explanation of our quantitative analysis in the Methods section such that it is more clear how our normalization procedure was performed. Furthermore, we will adapt **Fig. 5A-B** such that it better visualizes how we defined the regions for quantification. As per the Reviewer's suggestion, we will also apply a different experimental method to show that the tagged isoforms properly localize to actin-based structures. For this, we will attempt to use micropatterned cells to induce clearly define actin-bases structures (the crossbows as suggested by the Reviewer) and also explore the possibilities of investigating the differential localization in double-tagged cells. We will also reconsider the use of the term "cortex" for the region that is pointed out in **Fig. 5A-B**.

- Expression of tagged actins in yeast is a very nice idea but it would be far more informative to express the tagged forms as the only copy of actin. This can either be done by directly replacing endogenous actin gene in S. cerevisiae, or (if the tagged versions are not viable) - using the established plasmid shuffle system (express actin on counter-selectable plasmid, then knock out endogenous copy and introduce additional plasmid with tagged actin, then force original plasmid out). In the presence of endogenous S. cerevisiae actin the shown effects are very hard to interpret as nothing is known about relative protein levels (endogenous vs. introduced). Also, if constitutive expression of the ALFA nanobody is harmful for integration into cables, why not perform inducible expression of the nanobody and observe labeling after induction. For the live imaging a robust cable marker is needed, like Abp140-GFP. Finally, indicate the sequence differences between the used actin forms in yeast (supplementary figure with sequence alignment and clear indication of all variations)

We thank the reviewer for their positive comments and feedback regarding expression of IntAct variants in yeast. Currently, we have expressed IntAct as an extra copy in the presence of native Act1 of *S. cerevisiae*. All the IntAct variants have been expressed under a commonly used

constitutive TEF1 promoter. We agree with the Reviewer that it would be valuable to attempt to express the tagged forms as the only copy of actin.

Planned revisions:
1) As per the Reviewer's suggestion, we will attempt to make yeast strains with IntAct as the sole expressing actin copy by using the well-established 5-FOA-based plasmid shuffle system in yeast. We will use a Δact1 strain containing wildtype act1 in a centromeric ura-plasmid described in *Harrer et. al*, 2007 (generously shared by Prof. Jessica and Prof. Amberg at Upstate Medical University of New York, USA) and express IntAct exogenously via additional plasmids. Shuffling of these strains on 5-FOA will cause the loss of ura-plasmid containing the wildtype act1 copy and will determine whether yeast cells will be able to survive with IntAct as the sole source of actin. If the cells do survive with IntAct as a sole copy, we will perform subsequent analysis for assessing actin cytoskeleton organization under these conditions.
2) As the reviewer has mentioned, expression of NbALFA during live-cell imaging experiments hindered incorporation of IntAct into linear actin cables in yeast (**Suppl. Fig. S13**). As per the reviewer's suggestion, we will now try to create an inducible-expression system for the NbALFA-mNG and observe its effects on incorporation into formin-made actin cables after induction. We have already created NbALFA-mNG constructs under galactose-inducible GALS and GAL1 promoters and are currently constructing yeast strains for these experiments.
3) We will add an extra supplementary Figure to indicate the sequence differences of the various actin variants that we have expressed in yeast.

- For any claims regarding usability of tagged variants for isoform research it would be very important to characterize the known posttranslational modifications of tagged actin variants - are the differences between beta and gamma maintained on this level as well?

Planned revision: Following the Reviewer's suggestion, we will perform a western blot analysis to compare posttranslational modification (arginylation) of tagged and wildtype actins.

- In Fig. 2A only heterozygous FLAG-actin cells are used. Why not use a homozygous line (for both beta and gamma actin)? The nice band shift of the FLAG version would allow the precise quantification of the fraction of total actin covered by beta and gamma actin, which then could provide some additional info for the apparently weaker beta staining in Fig. 5 (if beta expression is simply weaker). This would be a very simple and useful advantage of the internal tags that could be widely applied.

In **Fig. 2A**, we used the heterozygous FLAG-actin cells to directly compare the production of β-actin from the knock-in allele and the wildtype allele in the same cells. The fact that the two bands observed in this western blot analysis (upper and lower) are almost the same (with the FLAG band being a bit more intense) provides the strongest indication that the tag does not interfere with the expression of actin. In **Suppl. Fig. 5D**, we show that the expression of β-actin is also unaffected in the hemizygous FLAG actin cells, which exclusively express tagged actin.

Planned revision: As per the Reviewer's suggestion, we will also add a western blot analysis on the expression of both actin isoforms and total actin in hemizygous cells.

- Fig. 3: control with N-terminal tag is missing. Also, why is it not possible to assay filament binding factors like Myosin, Filamin or alpha actinin - instead of co-IP a simple co-sedimentation assay with cell extracts in F-buffer should pick up any major difference in decoration of filaments

containing the ALFA tag. Using two speeds for centrifugation it might even be possible to observe effects on filament bundling. The best approach for this would of course be to purify tagged actins and perform in vitro assays but this is clearly beyond the scope of what the authors intended here. I personally think that a broad acceptance of the marker will only come once the biochemistry has been sufficiently characterized so this is a future direction I would strongly encourage.
We kindly refer to Page 19/20 for why we have not included the N-terminal control.

Planned revision: The co-sedimentation assay is an excellent suggestion by the reviewer. We will perform F/G-actin fractionation and assess the presence of several F-actin associated proteins in the F-actin fraction.

Technical issues
- The y-scales for Fig. 5C and D need to be identical to allow direct comparison

***Planned revision**:* We will adapt the scale of **Fig. 5D** to make it identical to **Fig. 5C**. Following the other suggestions of the reviewer, we will also critically evaluate our normalization procedure and present those numbers in **Fig. 5C-D** if the values turn out to be different.

- Pearson coefficient should not be normalized to a control value as its already a dimensionless parameter. Always report actual R-value - also remove R2 values for Pearson as this makes no sense in this context (not sure if it was a typo or intended).
We normalized the Pearson coefficient values for visual representation of the results. The majority of the raw coefficient values (more than 80%) are between 0.20 and 0.75 (see raw values in the associated excel file). Theoretically, Pearson coefficient values are possible between 1 (or-1 for negative correlations) and 0. The much smaller window in our values as compared to the theoretical window (0.55 vs 1) led us to normalize the values such that they can be presented on a scale from "maximum expected colocalization" to "minimum expected colocalization". In this way, the differences between the various tagged actins are much better appreciated in the Figure. As to reporting the R2, the Reviewer is correct. Reporting the R2 is an inadvertent mistake from our side and we will correct it.

Planned revision: We will change the R2 in the text to PCC or Pearson Correlation Coefficient.

- All values on subcellular regions (like stress fiber or cortex) dependet critically on the way thesese regions were thresholded or identified. Provide all details on how this was done in the methods section and ensure that adequate background subtraction and normalization is applied. Optimally, an unbiased (AI or automated) approach based on simple image statistics is used for this to avoid personal bias.

Planned revision: As also indicated above, we will add new experiments to better compare the localization of the isoforms in tagged and parental cells. These new experiments will also be accompanied by a more detailed explanation of how the regions were selected and quantified.

- In Fig. 2A only heterozygous FLAG-actin cells are used. Why not use a homozygous line (for both beta and gamma actin)? The nice band shift of the FLAG version would allow the precise quantification of the fraction of total actin covered by beta and gamma actin, which then could provide some additional info for the apparently weaker beta staining in Fig. 5 (if beta expression is simply weaker). This would be a very simple and useful advantage of the internal tags that could be widely applied.

In **Fig. 2A**, we used the heterozygous FLAG-actin cells to directly compare the production of β-actin from the knock-in allele and the wildtype allele in the same cells. The fact that the two bands observed in this western blot analysis (upper and lower) are almost the same (with the FLAG band being a bit more intense) provides the strongest indication that the tag does not interfere with the expression of actin. In **Suppl. Fig. 5D**, we show that the expression of β-actin is also unaffected in the hemizygous FLAG actin cells, which exclusively express tagged actin.

Planned revision: As per the Reviewer's suggestion, we will also add a western blot analysis on the expression of both actin isoforms and total actin in hemizygous cells.

- Fig. 2A has no loading control

We show this western blot to indicate that the WT actin and tagged actin are expressed at similar levels in the heterozygous knock-in cells. For this, no loading control is needed because we only compare the intensity of the upper band (tagged actin) with the lower band (WT actin).

- The RPE-1 data are confusing as several constructs show very different localization (completely cytosolic) to HT1080 cells and there is no possible explanation given for this. Maybe simply remove this data set?

We agree with the reviewer that the differences in the localization between some of the internally tagged actins between the HT1080 and RPE1 cells might be confusing, especially for the A230-A231 variant for example. Yet, the fact that also in these cells, the T229-A230 variant performs equally well as compared to N-terminally tagged actin is an important confirmation that this variant is properly integrated into actin-based structures, independent of cell type. This makes the support for choosing this variant to continue with our studies stronger. A possible explanation for the differences is that RPE1 cells in general tend to form more stress fibers as compared to the HT1080. Since the localization to stress fibers is different between the internally tagged actins, this may explain the differences observed in colocalization.

Planned revision: We will add a short text, in the Results or the Discussion, on the differences between the colocalization values between HT1080 and RPE1 cells.

- The angel measurements for lamellipodial actin is not very meaningful: the angel is determined for the radial bundles, which do not correspond to the Arp2/3 angel of single filaments and is likely the results of different nucleation factors, I would suggest to remove this. If angel measurement are really intended, cryoEM needs to be performed.

We apologize for this misapprehension from our side which is also noted by the other two reviewers. In the treadmilling videos of the lamellipodia in HT1080 cells, which were obtained using Airyscan super-resolution microscopy, we clearly observe a consistent filament formation at a constant angle, something which we interpreted as the angle between the mother filament and the daughter filament. After consulting the literature, we indeed have to admit that this cannot be interpreted as such and we will remove these datasets.

Planned revision: We will remove the datasets with the angle measurements (**Suppl. Fig. 7A-B**) from our manuscript.

- Replace all SEM with SD values - use at least 3 biological replicates (4D SEM of n=2)

Planned revision: We will carefully check our statistics and revise where appropriate.

Minor points
- Intro: after listing all the details already understood on actin isoforms it is not very convincing to simply state the molecular principles remain largely unclear (l 34) - maybe better "there is no way to study actin dynamics due to current limitations of specific antibodies to fixed samples. Interesting option would be actually to develop nanobodies that are isoform specific.

We will rephrase the text in the introduction. Regarding the development isoform-specific nanobodies. Although this sounds like a promising way forward, this would likely not result in isoform-specific targeting in living cells. Similar to the antibodies, isoform-specific nanobodies would have to be generated against the N-terminus which, under native conditions, is likely not available due to the occupation with actin-binding protein. Also, since the N-terminus is not structured, it may be extremely challenging to generate nanobodies against these epitopes.

- L 71: "involved" in the kinetics is not a good term - maybe affects or regulates....

We will rephrase the text.

- L148: "suspect" instead of "expect" - this clonal variation is actually a big danger of the employed approach as possible defects in actin organization could be masked by compensatory changes - it would generally be good to show critical data for at least 3 independent clones to rule out dominant selection effects.

We will rephrase. We agree that clonal variation could be a danger if actin levels are to be investigated. For future follow-up studies, we plan to make additional cell lines to avoid clone-specific conclusions.

Referees cross-commenting
I completely agree with the comments by reviewer 2 on the various missing controls - adding several or all of those will make the results much more convincing. The key for the adaptation of any new actin probe will be the level of confidence researchers have on the doumented effects. Even some negative effects on actin behavior (I am sure there will be some) should not prevent usage of the strategy as long as there is robust and convincing documentation of those effects. I also agree that including some basic in vitro characterization will go a long way to convince people dierectly working on actin (there is a very high level of biochemical understanding in that field).

Planned revision: We will perform the essential controls as suggested by Reviewer 2. Furthermore, for future experiments, we do envisage the production and purification of internally tagged actins and investigate their binding properties in *in vitro* reconstitution assays. We have already started with optimizing these approaches through our ongoing collaboration (KD, SP).

Reviewer #1 (Significance (Required)):
Significance: Very useful finding that can be applied to any question related to actin-dependent cellular processes (morphogenesis, cell division, cell polarization, cell migration etc.)
Strength: main finding convincing, strong genome edited cell lines
Limitations: application to study of isoforms very limited and data not convincing, statistics and image quantifications need improvement
Advance: identify new location for integral tagging of actin, which was not really possible before. The main relevance is for fundamental cell biology but the approach can also be applied to the study of disease variants in actin.
Audience: general cell biology - very broad interest

Revision Plan

In response to Reviewer 2:

Actin is highly sensitive to modifications, and tagging it with fluorescent proteins or even smaller motifs can affect its function. The most well-known example of this is that fission yeast where actin has been replaced with GFP-actin are inviable (Wu and Pollard, Science 2005) because the labeled actin cannot incorporate into the formin-dependent filaments that make up the cytokinetic ring. Subsequent experiments revealed that formins filter out GFP-actin monomers, as well as monomers that are labeled with smaller fluorescent motifs (Chen et al, J. Structural Biology 2012). Further, attempts to make mammalian cells lines where GFP-beta-actin was knocked into one allele resulted in extreme down-regulation of the GFP-labeled actin, indicating that there is some implicit toxicity with the labeled version. To my knowledge, all attempts at making homozygous GFP-actin knock-ins have been unsuccessful. Therefore, while GFP-actin or other labeled variants can be over-expressed in many different cell types with some success, there is always the question of how faithful the labeled actin represents bona fide actin localization and dynamics. To address this van Zwam et al. have developed a clever strategy of screening actin for internal motifs that can tolerate incorporation of a tag without affecting its function. They appear to have found a good candidate, named IntAct, and provide evidence that this tagging position allows the actin to be functional in both human and yeast cells. The work is very promising, and many of the assays performed satisfy the criteria of rigor and reproducibility. Importantly, the authors have created knock-in human cell lines where the tagged actin is expressed at normal levels, including a double allele knock-in that is viable and has normal proliferation and motility. Additionally, the authors show that labeled S. cerevisiae actin can incorporate into actin cables, which are formin dependent. IntAct constructs were shown to interact with several well-known actin binding proteins and localized well to many different actin structures. There was also interesting data obtained from tagging both beta and gamma actin in human cells. However, as an actin scientist eager for new probes to visualize actin in cells, there are still questions about the functionality of these probes. Addressing these issues, listed below, would alleviate the concerns I still have about IntActs after going through the manuscript. IntActs have the potential to have a large impact on cytoskeletal research if it can be rigorously documented that they are functionally as close to unlabeled actin as possible.

We thank the Reviewer for their constructive comments and general positive evaluation of our study.

1. There are no negative controls performed for either the fixed or live-cell imaging of IntAct. Since the fixed cell data is heavily reliant on the presence of flag-labeled puncta at actin filaments, it is important to show that the immunocytochemistry protocol doesn't produce anything that would mimic the localization of actin. For the live cell data, there has been no effort made to show that the binding of the nanobody to the ALFA tag on InAct is specific.

Planned revision: We will add the following controls to exclude that any of the labeling procedures produces anything that would mimic the localization of actin: ***1)*** Immunofluorescence staining of the used tags (FLAG/ALFA) in cells that do not have tagged actins ***2)*** Expression of ALFA-Nb-GFP and ALFA-Nb-mScarlet in cells that do not have tagged actins ***3)*** Expression of free GFP in cells that have tagged actins. We will co-stain these cells with phalloidin to visualize F-actin and determine if any signal is specifically localized to the actin cytoskeleton.

Review COMMONS

- 3. It is not addressed if expressing the ALFA-Nb-GFP construct in ALFA-IntAct cells alter actin properties? This is essential information for live cell imaging experiments.

Planned revision (Experiments already performed): We have already performed proliferation and migration experiments in cells that stably express the ALFA-Nb-GFP. These data indicated that proliferation and migration are not affected by the presence of the nanobody (**Fig. 1**, to be included in the revised manuscript) and these data will be included in the revised manuscript. To note, in the original manuscript, we already showed that treadmilling of actin at the lamellipodia is not affected by the presence of the ALFA-Nb-GFP.

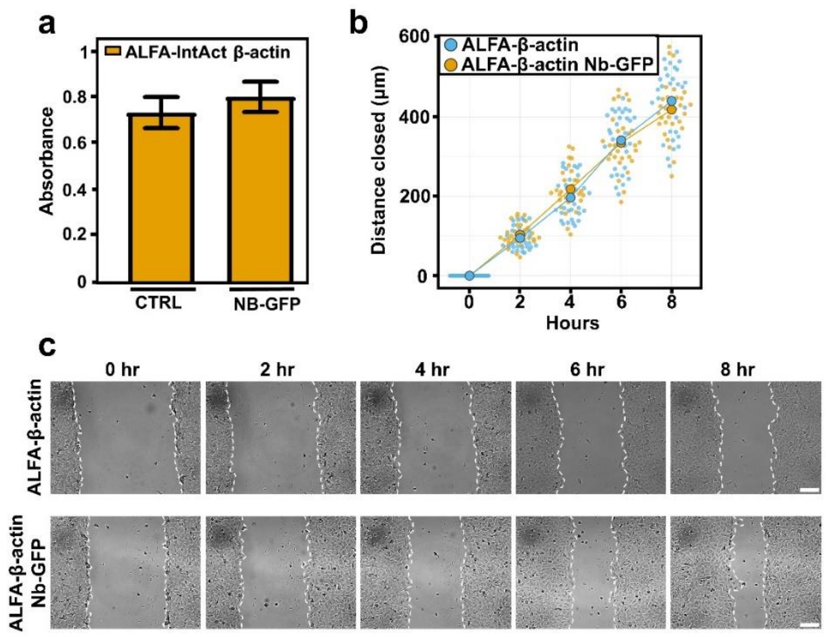

Figure 1. ***a)*** Proliferation and ***b-c)*** migration are not affected in ALFA-IntAct cells that stably express ALFA-Nb-GFP.

5. To assess lamellapodia architecture, "branched actin angle" is measured using AiryScan imaging of actin filaments. This type of microscopy does not offer the ability to image individual actin filaments; what is actually being measured is the orientation of actin bundles to each other. It should be impossible to image the orientation of actin filaments in Arp2/3 dendritic networks and it is surprising that the measurements average to 70 degrees. A suitable substitute for this would be to measure the size and amount of F-actin in phalloidin-stained lamellipodia using kymograph analysis.

We apologize for this misapprehension from our side which is also noted by the other two reviewers. In the treadmilling videos of the lamellipodia in HT1080 cells, which were obtained using Airyscan super-resolution microscopy, we clearly observe a consistent filament formation at a constant angle, something which we interpreted as the angle between the mother filament and the daughter filament. After consulting the literature, we indeed have to admit that this cannot be interpreted as such and we will remove these datasets.

Planned revision: We will remove the datasets with the angle measurements (**Suppl. Fig. 7A-B**) from our manuscript.

- 6. Was it possible to make an IntAct gene substitution in yeast?

Planned revision: We thank the reviewer for this interesting question and as also suggested by Reviewer 1, we are now constructing yeast strains with IntAct as the sole expressing actin copy

by using the well-established plasmid shuffle system in yeast. The results of these experiments will determine the ability of IntAct to completely substitute actin function in yeast.

Also, while this is not necessary for this manuscript, making a fission yeast strain where actin has been substituted with IntAct and demonstrating that IntAct gets incorporated into the cytoplasmic ring and into Cdc12p-polymerized filaments would alleviate MANY potential concerns people would have about these probes by directly assessing situations were other labeled actins have been documented to fail. Along the same lines, it would have been nice to see a comparison in some of the assays of ALFA-IntAct and GFP-actin or another labeled actin variant.

We appreciate the reviewer for their constructive feedback and completely agree that it is important to document how IntAct behaves in scenarios where other labelled actins have failed. As a proof of principle, IntAct incorporates into both formin- and Arp2/3- made linear and branched actin filaments in yeast (**Fig.5E, Suppl. Fig. 14**) and this data shows that IntAct labelling strategy is the first to achieve good integration into both these structures as previous efforts with labelled actin such as GFP-Actin fail to incorporate into formin-made actin filaments (Doyle et al., PNAS, 1996). Thus, we believe that IntAct does perform better than other labelled actins in yeast, although, further optimizations are required to overcome limitations regarding incorporation into actin cables in the presence of the ALFA nanobody.

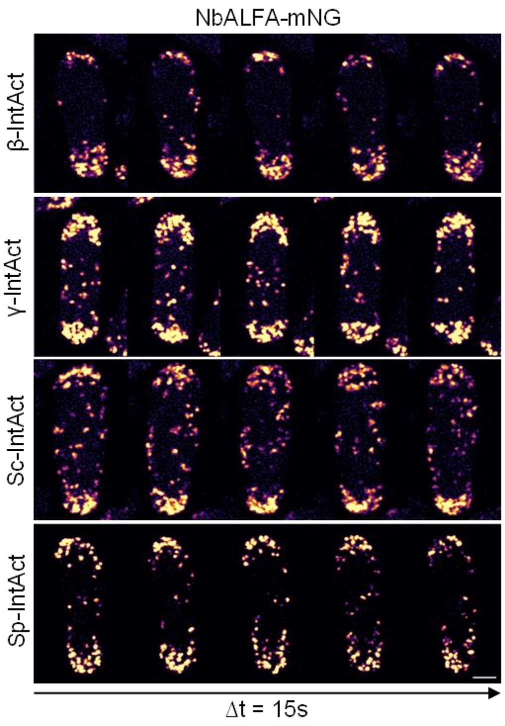

Figure 2. Representative montages of *S. pombe* cells co-expressing indicated IntAct variants and NbALFA-mNG. (Scale Bar: 2μm)

<u>Planned revision (Experiments already performed):</u>

We have already extended applicability of IntAct to another well-known fungal model system, the fission yeast *Schizosaccharomyces pombe* (*S. pombe*). We expressed IntAct variants of human β- and γ- actin, budding yeast actin (Sc-IntAct) and fission yeast actin (Sp-IntAct) from an exogenous plasmid under the native *S. pombe* actin promoter in an *S. pombe* strain that constitutively expresses the Nb-ALFA-mNG. Live-cell microscopy of *S. pombe* cells expressing these proteins revealed that all IntAct localize to actin patch-like structures located at the cell poles and cell division site (during cytokinesis) (**Fig. 2**, to be included in the revised manuscript). These structures show similar dynamics as reported for actin patches of *S. pombe* previously (Pelham et al., Nat Cell Biol, 2001). These preliminary results suggest that IntAct proteins show a similar localization pattern to only branched actin networks found in the actin patches of *S. pombe* like we had previously observed for the budding yeast, *S. cerevisiae* (**Fig. S13** in manuscript). The underlying mechanism for this exclusion from linear actin cable network from both budding and fission yeast remain unknown and may represent an inherent specificity and sensitivity of yeast formins. Our current and future experiments will express IntAct variants in

absence of the ALFA nanobody and determine the level of incorporation into actin cables, patches, and actomyosin ring.

__Planned revision (Experiments already performed):__ We have also already performed a quantitative analysis to ascertain the effect of Sc-IntAct expression of cortical actin patch dynamics which represent sites of endocytosis in yeast (Young et al., J Cell Biol, 2004; Winter et al., Curr Biol, 1997). We compared actin cortical patch lifetimes between wildtype cells and cells expressing Sc-Act1 or Sc-IntAct as an extra copy. We used Abp1-3xmcherry as a marker for actin patches and quantified the time window between the appearance and disappearance of a patch (actin patch lifetime) from time-lapse microscopy experiments. Our preliminary results indicate that actin patch lifetimes are unaffected by exogenous expression of both Sc-Act1 or Sc-IntAct suggesting that IntAct does not negatively influence or alter actin patch dynamics (**Fig. 3**, to be included in the revised manuscript). These observations suggest its applicability as a direct visualization strategy for actin at the cortical patches in budding yeast alongside existing surrogate markers like Abp1, Arc15, etc (Goode et al., Genetics, 2015; Wirshing et al., J Cell Biol, 2023).

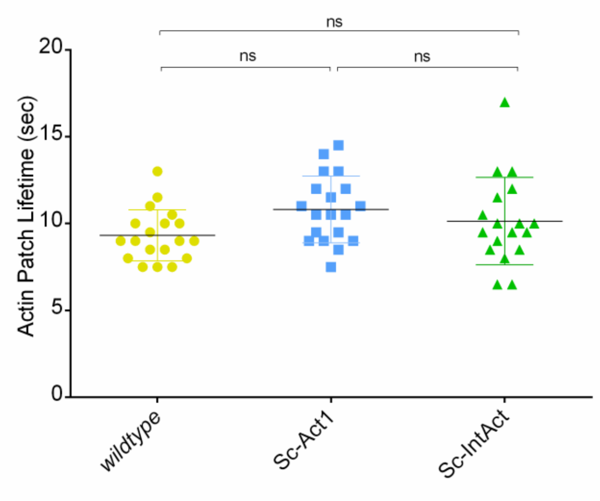

Figure 3. Graph showing actin patch lifetimes (seconds) for indicated strains (wildtype, wildtype cells expressing Sc-Act1, wildtype cells expressing Sc-IntAct). Abp1-3xmCherry was used as an actin patch marker. (Kruskal-Wallis test was performed to determine significance)

In response to Reviewer 3:

Summary:

This paper tackles a new strategy to tag actin in cells, by identifying that incorporation of a tag of moderate size in subdomain 4 of actin minimally affects actin dynamics in cells, and does not perturb its interaction with known partners, as observed in pull-down assays.

Major comments:

The paper is interesting and experiments are convincing.

My main concerns are the following :

- It is now well established that actin plays active and important roles in the nucleus : is ALFA-actin correctly translocated to the nucleus ?

__Planned revision:__ This is an interesting suggestion. We will perform nuclear-cytosol fractionation experiments and determine whether ALFA-actin is still correctly translocated to the nucleus.

Minor comments:

Below are points that could be addressed by the authors to improve the manuscript readability and highlight some important points that are sometimes missing or are not properly discussed:

-line 40 "...but the distinct N-terminal epitope is not available under native conditions preventing" is a bit too obscure. Can the authors say clearly what is meant by 'native conditions'?

In our understanding, the term 'native' is generally used when referring to conditions in which proteins are in their natural state, without alterations due to heat or denaturants, and possibly also still interacting with their binding partners. We will rephrase to better indicate that in this specific case, we mean that the region that harbors the N-terminus is usually occupied by actin-binding proteins, preventing the binding of the antibody due to steric hindrance.

- figure 1A : make a clearer correspondance between the number shown in panel A and the amino acid numbers displayed in panel C and G.

This is a good point, we will add extra annotation in the graph to better link the panels with each other. We will also add additional annotation in **Fig. 1D-F** for the same purpose.

- figure 1A : it could be informative to indicate subdomains in this panel.

We will add the numbers for the subdomains in **Fig. 1A**.

- figure 1C : normalized correlation cell : I am not sure I understand how the normalization of the Pearson coefficient is done. It is therefore not clear how can it >1 or >-1 ? This should be clearly explained in the method section of the paper.

We will better explain the normalization procedure in the Methods section.

- figure S4 : comes a bit too early when ALFA-actin has not been yet introduced in the main text. Please, reposition this part or provide data with the FLAG-tag version.

This is a good point and completely overlooked by us. We will introduce this Figure later such that the ALFA tag is already introduced.

- section starting line 121 : this section should be better motivated = Why are different tags being tested ? This comes later in the discussion, but the reader fails at following the reasoning/motivation here.

We will add extra motivation for why we added multiple tags.

- figure 2D, line 145 "We also evaluated actin protein expression in the homozygous ALFA-β-actin cells and this showed that the total amount of β-actin was slightly lower in the ALFA-β-actin cells compared to parental HT1080 cells (Fig. 2C-D)." 'Slightly' is not a very quantitative nor accurate term. please rephrase. Besides, a statistical test for the paired data would also be informative. Besides, data in figure S6B-D indeed show a correlated increase in the expression of Gamma-actin that compensate for the decrease in the Beta-actin level in ALFA-Beta-actin. Can the authors explain why they conclude otherwise?

This indeed is an important point and we will change the phrasing of this section to provide a more quantitative and accurate description of the western blot quantifications.

- figure S7B: I am not ure anyone has ever reported measurement of angle of branched actin filament using epifluorescence microscopy. I would remove this panel, or the authors should explain how this measurement can be done objectively.

We apologize for this misapprehension from our side which is also noted by the other two reviewers. In the treadmilling videos of the lamellipodia in HT1080 cells, which were obtained using Airyscan super-resolution microscopy, we clearly observe a consistent filament formation at a constant angle, something which we interpreted as the angle between the mother filament

and the daughter filament. After consulting the literature, we indeed have to admit that this cannot be interpreted as such and we will remove these datasets.
Planned revision: We will remove the datasets with the angle measurements (**Suppl. Fig. 7A-B**) from our manuscript.

- Figure 2F : can the authors comment on the (significant ?) lower value for FLAG-tag actin ?
The lower value for FLAG-tag actin has likely to do with the properties of the antibody and suitability for immunofluorescence. For reason that we do not know, we usually detect more background for the FLAG tag antibody as compared to the other antibodies/ALFA tag nanobody. Since the Pearson correlation coefficient quickly decreases with suboptimal labeling, this is likely the reason that the values for FLAG-actin are lower as compared to the other tagged actins. Importantly, in our biochemistry experiments (F/G-actin), we detect no difference between FLAG-actin and ALFA-actin indicating that it is rather the immunofluorescence and sensitive Pearson correlation analysis than the integration of actin that causes this difference.

- line 205 "The results from these experiments show that both DIAPH1 and FMNL2 associate with ALFA-β-actin (Fig. 3D),". It is not so obvious that these formins directly interact with monomeric actin via their FH2 domains in co-immunoprecipitation assays. It might very well be mediated by the interaction with profilin, that in turn bind to the FH1 domain of formins. For me, this assay does not make a correct proof that epitope-labelled actin do not interfere with formin activity.
The point that the co-immunoprecipitation does not demonstrate direct interactions between formins and actin is well taken. We, however, do not claim that this assay proofs that formin activity, or formin-based integration of actin monomers, is similar with tagged actin as compared to wildtype actin. Nonetheless, we will critically re-evaluate the relevant passages and rephrase the text to avoid any confusion.

- figure 5C&D : both graph should use the same scale for the y-axis for easier comparison.
We will adapt the scale of **Fig. 5D** to make it identical to **Fig. 5C**. Following the other suggestions of the Reviewer (and of Reviewer #1), we will also critically evaluate our normalization procedure and present those numbers in the Figures if the values turn out to be different.

- figure 5D: I think the way the ratio is performed is misleading. Why not look at the Beta/Gamma ratio using the isoform specific antibodies used in parental cells, and show the results for ALFA-Beta-actin and for ALFA-Gamma-actin separately ?
We kindly refer to our answer to Reviewer #1 on Page 2 for a detailed explanation on the experimental challenge of comparing the localization of wildtype and tagged actin isoforms.

Planned revision: We will critically evaluate our normalization procedure and present those numbers in the Figures if the values turn out to be different. Furthermore, we will add a different experimental method to show that the tagged isoforms properly localize to actin-based structures. For this, we will attempt to use micropatterned cells to induce clearly define actin-bases structures and also explore the possibilities of investigating the differential localization in double-tagged cells.

- The limitation observed for unbranched cables in yeast that nanobody-tagged ALFA-actin does not incorporate correctly should be discussed and stressed further in the discussion, as it might prove to be a strong limitation for live-cell imaging to reliably study any type of actin networks.

We acknowledge the reviewer's concern regarding the inability of ALFA-tagged actin to incorporate into yeast actin cables when NbALFA is co-expressed and will discuss this point further in the revised manuscript. We have now observed the same limitation for fission yeast actin cables as well and combined, these observations may represent a tighter control and sensitivity of yeast formins towards any perturbations in actin size (since NbALFA binds to ALFA tag with picomolar affinity). To address this issue and as also suggested by Reviewer 1, we are now creating yeast strains with inducible control of NbALFA expression under GALS/GAL1 promoters and observe the labelling of actin structures after this approach. Additionally, expression of variants of NbALFA with high dissociation rates may also allow labelling of actin cables and would be certainly worth a try in the future. A structural comparison between mammalian and yeast formins may be required to shed some light on the molecular basis of this fundamental difference.

However, since in the absence of the nanobody, this limitation is overcome (**Fig. 5E, Suppl. Fig. 14)**, we believe that with additional modifications and fast developments in imaging technologies, this limitation can be overcome in the future. Thus, IntAct as a labeling strategy represents an advancement over existing labelled actins with the most important aspect being the identification of the T229/A230 residue pair to be permissive for integration of various tags even as large as GFP11 fragment including a linker (26AA) (**Reviewer Fig. 2**). Importantly, the T229/A230 site is conserved across many organisms (such as Chlamydomonas reinhardatii, Cryptococcus neoformans, etc) and may act as a framework to study the actin cytoskeleton especially in organisms where known surrogate markers like phalloidin and Lifeact may not work or work only sub optimally.

Reviewer #3 (Significance (Required)):
General assessment:
This paper provides a new tagging strategy to monitor actin activity in cells, by specifically inserting the tag along the amino acid sequence.
Advance:
This is a very useful tool, as most existing available probes bind to actin in regions that are common to many other actin binding proteins. The authors provide extensive experiments to validate that tagged-actin are functional and do not perturb the actin expression level, actin network architecture nor dynamics.
Audience:
This research paper will be of interest to a rather broad audience (many cell biologists) that are either sutyding actin dynamics or know that actin is involved in the cell functions they study.
Expertise:
My expertise is in vitro actin biochemistry.

3. Description of the revisions that have already been incorporated in the transferred manuscript

Review COMMONS

4. Description of analyses that authors prefer not to carry out

In Response to Reviewer 1:

Major points

- "…Also, the unique selling point of the strategy is the possibility of actual live imaging of specific isoforms. Cells that have stably integrated double tags and then transiently express nanobodies for ALFA and either AU5 or FLAG (or other if those don't exist) would make this possible. Considering the work already done in this manuscript, such an approach should actually be possible - did the authors attempt this or is there are reason it is not discussed?.."

Live cell imaging:

We agree with the Reviewer that it would be very valuable to attempt simultaneous live cell imaging of two isoforms. Yet, for this, we would need two tag/fluorophore systems that allow the visualization of internally tagged isoforms in living cells. As presented in our original manuscript, we have successfully inserted many different epitope tags (FLAG/AU1/AU5/ALFA) in the T229/A230 position to demonstrate the versatility of our tagging approach. Yet, despite significant efforts to identify a second tag/fluorophore system that would allow isoform-specific live cell imaging, we only succeeded in designing one strategy to perform live cell imaging, i.e. with the ALFA tag (Götzke, Nat Comms, 2019). Part of the reason for this is that so far, no high affinity nanobodies have been generated against the classical epitope tags (FLAG, AU5 etc.). This is an established challenge since classical epitope tags are typically linear/unstructured while nanobodies require folded secondary structures for epitope recognition such as alpha helices (the ALFA tag was specifically designed as such).

Besides the successful ALFA tag approach we have tried the following additional approaches for live cell imaging: ***1)*** full-length GFP, ***2)*** full-length GFP with linker, ***3)*** GFP11 (to complement with GFP1-10 (Cabantous et al., Nat Biotech, 2005) ***4)*** GFP11 with linker ***5)*** FLAG Frankenbodies (Zhao et al., Nat Comms, 2019; Liu et al., Genes Cells, 2021) in FLAG IntAct cells and ***6)*** Tetracysteine/FlAsH labeling. Importantly, each of these additional internally tagged actins, except for those that contained full-length GFP, showed a high colocalization with the cytoskeleton, again demonstrating the versatility of the T229/A230 position to tag actin. Unfortunately, none of these approaches satisfactorily visualized the actin isoforms in living cells. We will therefore briefly summarize our findings here for the Reviewer:

(***1-2, integration of full-length GFP and GFP with linker***) Probably not surprisingly, but integrating the entire coding sequence of GFP or GFP flanked by linkers (each 5AA in length) within the T229/A230 position did not results in a proper localization of actin (**Reviewer Fig. 1**).

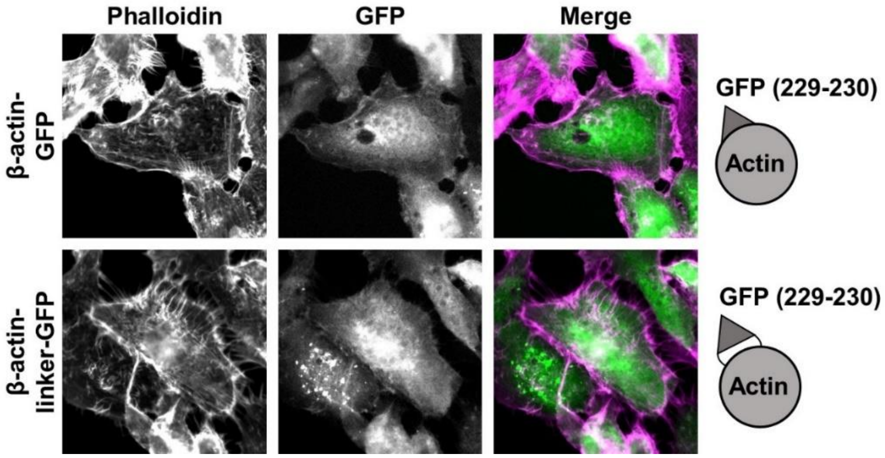

Reviewer Figure 1. Mislocalization of internally tagged GFP-actin.

(***3-4, integration of GFP11 and GFP11 with linker***) Next, we assessed the localization of the GFP11 tagged actin versions (GFP11: 16AA, GFP11+linker: 26AA). Because GFP11 is not visible without GFP1-10 complementation, we also tagged actin at the N-terminus simply for proof of concept where the internally tagged actins would end up. Interestingly, both GFP11-actin and GFP11+linker-actin properly integrated within the cytoskeleton as demonstrated by the FLAG staining (**Reviewer Fig. 2**). This again demonstrates the versatility of the T229/A230 position and strongly suggests that even the integration of ***26AA*** within this position does only minimally affect the polymerization of actin into the cytoskeleton.

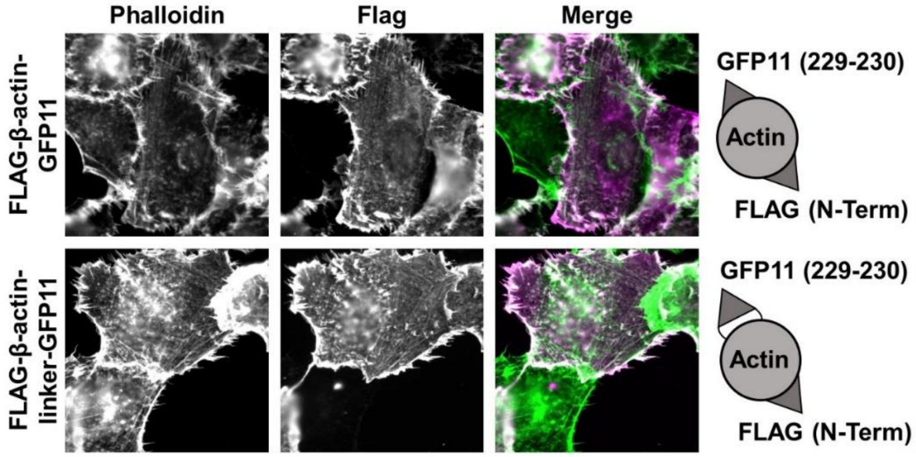

Reviewer Figure 2. Proper integration of GFP11-actin into the F-actin cytoskeleton.

(***3-4***) After confirmation of the proper integration of GP11-actin and GFP+linker-actin we continue to express the GFP1-10 in these cells. Unfortunately, this resulted in no or only very minimal localization of the actin to the cytoskeleton (**Reviewer Fig. 3**), demonstrating that GFP-complementation hampers the integration into the cytoskeleton.

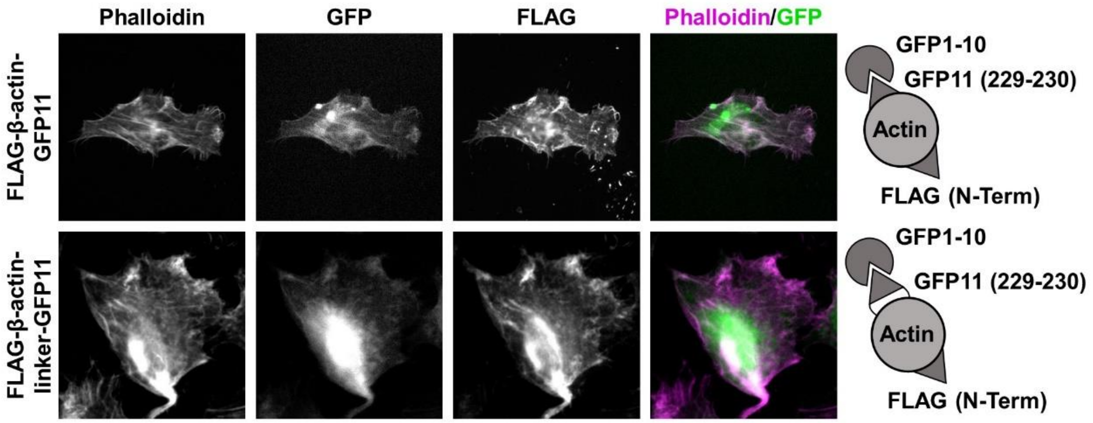

Reviewer Figure 3. Minimal colocalization between complemented GFP11-actin and phalloidin.

(***5, use of FLAG Frankenbodies***) We also expressed FLAG Frankenbodies into our FLAG IntAct cells in an attempt to visualize the isoforms in living cells. FLAG Frankenbodies are single chain antibodies fused to GFP and can be expressed in cells to visualize FLAG-tagged proteins (Liu et al., Genes Cells, 2021). Although a cytoskeletal labeling was indeed discernable in some cells, the FLAG Frankenbody signal overlapped much less with the total actin signal as compared to the FLAG immunofluorescence labeling, indicating that the incorporation of the FLAG-tagged actin was much less in the presence of the FLAG Frankenbody. Also, a significant fraction of the cells demonstrated a homogenous cytosolic signal (**Reviewer Fig. 4**).

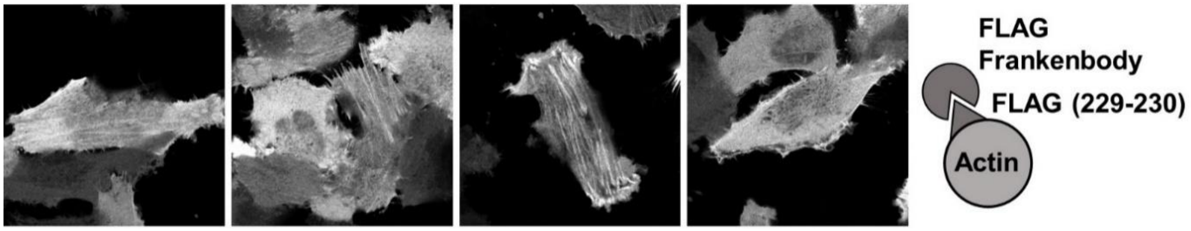

Reviewer Figure 4. Localization of FLAG Frankenbodies in FLAG IntAct cells

(***6, Use of tetracysteine/FlAsH***) Although the tetracysteine tag/FlAsH system is widely known to induce artefacts, we still aimed to evaluate if for live cell imaging of IntAct actins. Similar to GFP11, we first determined the integration of tetracysteine-actin into the cytoskeleton with the use of an additional N-terminal FLAG tag and demonstrate that it was properly integrated into the actin cytoskeleton (**Reviewer Fig. 5**). Unfortunately, after brief incubation with FlAsH-EDT2, we noted *1)* a significant amount of background fluorescence, preventing proper actin visualization (**Reviewer Fig. 6**) and *2)* that the cell became static indicating toxicity of the FlAsH-EDT2

compound (Data not shown). Titrating down the amount of FlAsH-EDT2 did not alleviate these drawbacks and only resulted in less fluorescence.

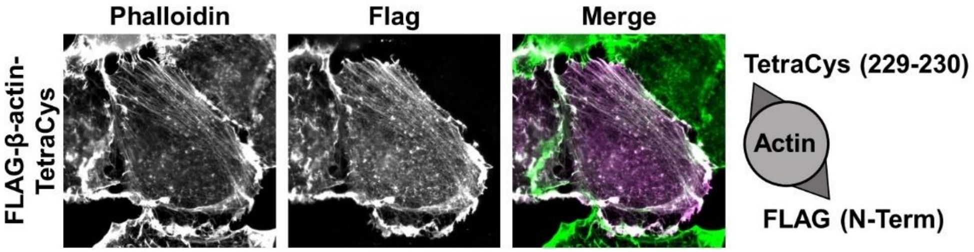

Reviewer Figure 5. Colocalization of TetraCys-actin with F-actin.

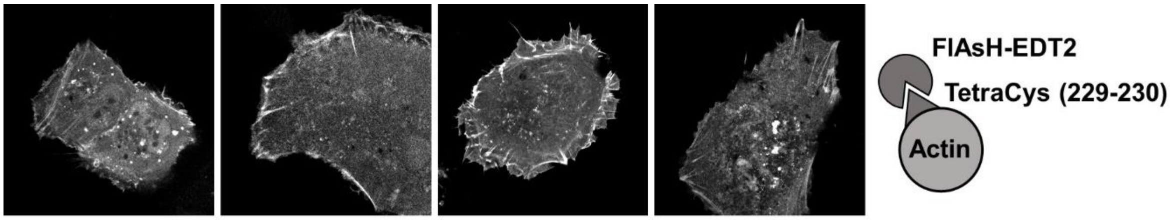

Reviewer Figure 6. Visualization of the TetraCys tag using FlAsH-EDT2.

Overall, based on these experiments, we concluded that the T229/A230 position itself is very versatile, as demonstrated by the proper localization of the GFP11-actin variants and the TetraCys-actin. At the same time, none of these tag/fluorophore systems properly visualized actin in living cells. Although we are unsure what the reason is for this, it is easily imaginable that the on/off kinetics of the split GFP system and the FLAG Frankenbodies are suboptimal to allow for the rapid and continuous integration of actin monomers into the F-actin cytoskeleton. We therefore also concluded that currently, the ALFA tag/nanobody system is apparently unique in its ability to visualize epitope tagged actin in living cells (as shown in the manuscript). For simultaneous visualization of multiple isoforms, we rely on progress on the development of novel nanobody-based tags, something we hope the Reviewer will agree is outside the scope of the current work.

- The authors make a point of comparing the internally tagged actin to N-terminal tags that are mostly functional but have been shown to affect translational efficiency. I would strongly suggest to include N-terminally tagged actin as control for all assays in this study. Also for the physiological assays (retrograde flow, wound healing), a positive control is missing that shows some effect. Previous studies showed defects with transiently expressed actin with an N-terminal GFP. As retrograde flow measurements are very sensitive to the exact position of the kymographs and wound healing assays is a very crude and indirect readout, such a positive control is essential.

We acknowledge that N-terminally tagged actin has been used extensively for actin research (especially before the introduction of Lifeact). For our studies, however, we were specifically interested in whether the internally tagged actins show similar characteristics as compared to wildtype actin. We have not included N-terminally tagged actin in all of our experiments, since this would not affect our conclusions with respect to the functionality of our internally tagged actins. We expect that for future investigations to for example further establish the importance of actin N-terminal modifications in the differential regulation of actin isoforms, the comparison between

internally and N-terminally tagged actins could be very instrumental. Yet, we consider this comparison outside the scope of the current manuscript. For now, the results in the manuscript provide evidence that our approach is unique with respect to the fact that it allows isoform-specific tagging without manipulating the N-terminus. As such, our internal tagging system complements the already existing repertoire of actin reporting methods (N-terminal fusion, Lifeact, F-Tractin, actin nanobodies) and allows researchers to study so far unknown properties of actin variants.

- As the authors clearly show good integration of several tagged actins into filaments I would expand the structural characterization: perform alpha fold predictions of actin monomer structures including the various tags to show the expected orientation. It is striking that the only integration site that seems to work well is at the last position of a short helix, indicating that the orientation of the integrated peptide might be fixed in space and be optimal to minimize interference. Also, a docking of the tag onto the recently published cryoEM structures of the actin filament should be shown to indicate where it resides compared to tropomyosin or the major groove where most side binding proteins seem to bind.

We already performed AlphaFold predictions of the tagged actin monomers (**Reviewer Fig. 7A-C**), but we have decided to not include these predictions in the manuscript because of two reasons. First and foremost, while the prediction confidence of the non-tagged region is very high (pLDDT > 90), the prediction confidence of the tagged region is very low (pLDDT < 50 for both FLAG and the ALFA tag). As indicated on the AlphaFold website (https://alphafold.ebi.ac.uk/faq), pLDDT values below 70 should be treated with caution and values below 50 should not be interpreted. Intriguingly, the low confidence aligns with the fact that for both tags, the prediction does not match with known features of the tag. The FLAG tag should be a linear/unstructured region in order to be recognized by the antibody and the ALFA tag should organize into an alpha helix (Götzke et al., Nat Comms, 2019). Yet, in the prediction, the FLAG tag partially continues as an alpha helix and the ALFA tag is only a small helix with part of the tag being unstructured. Second, more minor, reason for not including the predictions is that AlphaFold does not predict to what extend the tag is flexible, which means that even if the tagged region is predicted correctly, it is difficult to say whether the regions will interfere with binding of proteins.

Despite the low prediction confidence, we used the published actin-tropomyosin cryoEM structure (von der Ecken et al., Nature, 2015) to replace WT actin with ALFA tag actin and the results are shown below (**Reviewer Fig. 7D-E**). Again, although results should be interpreted with caution, the tag does not seem to obstruct monomer-monomer interactions within an F-actin filament and also the tropomyosin binding surface is relatively distant from the tag region, suggesting that these interactions are likely not disturbed by introducing the tag.

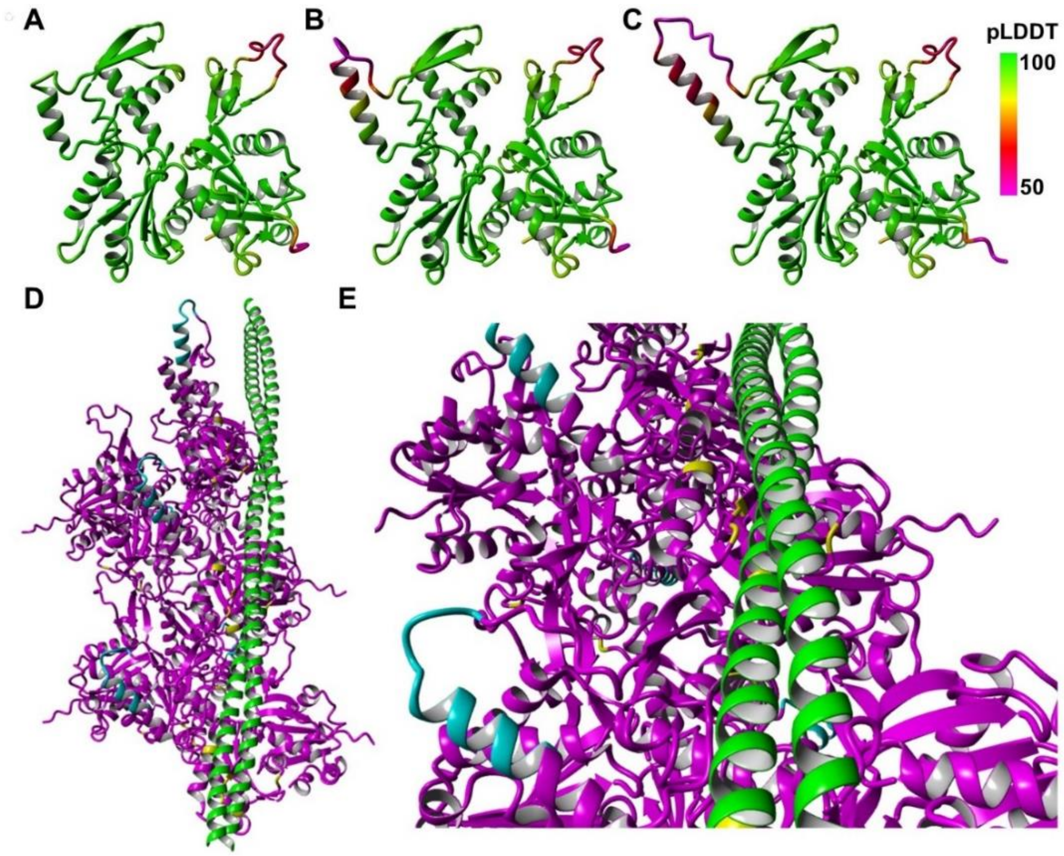

Reviewer Figure 7. Predicted structure of tagged actins and overlay with actin-tropomyosin complex.
A-C) AlphaFold predictions of WT actin (A), FLAG IntAct actin (B) and ALFA IntAct actin (C). The color indicates the pLDDT score which is a measure for the prediction confidence. Besides the D-loop, the prediction of the tagged region is very low and is difficult to interpret based on the prediction. **D-E)** Two views of the cryoEM structure of the F-actin-tropomyosin complex (PDB: 3J8A) with the WT actin monomers being replaced for ALFA IntAct actin. Actin is colored magenta with the ALFA tag in cyan and the residues that facilitate interaction with tropomyosin in yellow. Tropomyosin is colored green.

Technical issues
- There is no scale for the color coding in Fig. 5A, B
We deliberately did not add a numerical scale because the images are normalized which means that presenting the actual numbers might be misleading. The numbers could be interpreted as if they actually present the amount of β-actin relative to γ-actin which is not the case due to staining differences and the normalization procedure. Since the affinities of the β- and γ-actin antibodies are different, it has so far been impossible to reliably estimate the proportion of β- vs γ-actin in actin filaments.

In Response to Reviewer 2:
2. The homozygous ALFA-tagged IntAct cells have a 50% reduction in the amount of actin expression (Fig. 2D). What is the F:G ratio in these cells? The F:G measurement is only shown for the FLAG-tagged heterozygous IntAct cells, which have the worst co-localization with phalloidin (Fig. 2F) and were not used for subsequent figures. I appreciate that motility and

proliferation were measured and shown to not be affected (Fig. 4D,E) , but in our lab reducing the amount of polymerized actin by 50% (which may be more in ALFA-tagged IntAct cells if the F:G changes) has catastrophic effects on other cytoskeletal and organelle systems. Since the homozygous ALFA IntAct cells are the main ones used in the manuscript, they should be the ones that are fully characterized.

We would like to point out that the reduction is only 20-25 percent depending on the specific western blot analysis and the loading control. Still, the Reviewer is correct about the necessity of the F:G actin measurements of the ALFA-tagged IntAct cells and we therefore already included those as **Suppl. Fig. 9** in the original manuscript (text on page 9). The quantification of these assays clearly demonstrated that the F-G actin ratio in the ALFA-tagged IntAct cells is the same as in parental cells.

4. It is not addressed how much of the ALFA-IntAct gets labeled with ALFA-Nb-GFP and how uniform the labelling.

We do not understand this specific request of the Reviewer. To our knowledge, it is not possible to assess how much of a probe (in this case the ALFA-Nb-GFP) binds the target (in this case the ALFA-IntAct actins) in living cells. This is not only the case for the ALFA-Nb-GFP but also for any other probe. As an example, when expressing Lifeact, we also do not know how much of the actin molecules within F-actin get labeled with Lifeact and how uniform the labeling is. From the results of the live-cell imaging we can only conclude that the binding is at least so effective that we can readily observe and discern all the actin-based structures that are also observed by Lifeact (see **Suppl. Fig. 8** for Lifeact-GFP/ALFA-Nb-mScarlet cotransfection). Whether the regions that do not have F-actin only contain ALFA-Nb-GFP that is bound to actin monomers or also contains a significant fraction of free ALFA-Nb-GFP seems an issue that cannot be addressed.

In response to Reviewer #3:

- Varland et al, is reporting a phosphorylation on Thr229 : I think the authors should mention and discuss this potential PTM that could be affected in IntAct.

We thank the Reviewer for pointing this out. We are aware of this review that includes phosphorylation on Thr229 as a possible PTM. Yet, this PTM is only reported in one of the Tables of the Review and not further discussed in the text. It is also unclear how the authors determined that Thr229 is a possible phosphorylation site except for the notion that this residue is a threonine and exposed at the surface of the actin molecule. Together with the fact that there is no evidence from primary studies that Thr229 is phosphorylated, we therefore decided to not include it in our discussion.

- The sequence in subdomain 4 (the alpha helix containing T229A230) is extremely conserved in animals, as well as in between the 6 human actin isoforms. This usually indicates a strong selection pressure on the residues. I think the authors should discuss how surprising it is that the T229A230 position can accomodate various tags while it is probably the place of interaction with other proteins and is playing an important role in the mechanical structural integrity of the actin itself.

We thank the Reviewer for bringing up this important point. To a certain extent, the conservation argument is true for all of the residues/domains in actin. Any manipulation will change a conserved part of the actin molecule in one way or another and thereby potentially modify its function. This is also evident from the fact that for most of the internally tagged actins, we observed a very poor

colocalization with the actin cytoskeleton (**Fig. 1**). While for the T229/A230, we have not observed any major effects yet, this certainly does not mean that no further changes or defects will be uncovered in future experiments. Nonetheless, since our approach is unique with respect to the fact that it allows isoform-specific tagging without manipulating the N-terminus, our internal tagging system complements the already existing repertoire of actin reporting methods (N-terminal fusion, Lifeact, F-Tractin, actin nanobodies) and allows researchers to study so far unknown properties of actin variants. We have already included in the discussion that, at this point, we can only speculate as to why this variant performs much better than the others (Page 16 of the manuscript) and that possible explanations are the location at the inner domain and the higher structural plasticity of this region as compared to the rest of the molecule, as found during an alanine mutagenesis screen (Rommelaere et al., Structure, 2003).

- OPTIONAL: one may regret that there is no classical in vitro assays, such as pyrene assays to assess some kinetcis parameters on epitope-tagged actins. I guess this would make the paper a bit too large. Although, it will prove useful to better understand how much formin activity is affected (see below)

For further biochemical characterization and a detailed investigation of the precise assembly kinetics of the tagged actins, we (KD, SP) are already working together to set up *in vitro* reconstitution experiments. Yet, as also indicated by the Reviewer, we consider these experiments outside of the scope of the current work.
