# Supplementary material for: IntAct: A nondisruptive internal tagging strategy to study the organization and function of actin isoforms
Source: PLoS Biol. 2024 Mar 11;22(3):e3002551. doi: 10.1371/journal.pbio.3002551 (PMC10957077; doi:10.1371/journal.pbio.3002551)
Supplement: Attachment — Submitted filename: ResponsetoReviewers_PLoS Biology_Revision.pdf [file pbio.3002551.s036.pdf]

Reviewer #1 (Evidence, reproducibility and clarity (Required)):

In this study, the authors generate several variants of actin that are internally tagged with short peptide tags. They identify one particular position that is able to tolerate various tags of 5-10 amino acids and still shows largely unaltered behavior in cells. They study incorporation of their tagged actins into filaments, characterize the interactions of G-actin variants with different associated proteins and show that retrograde actin flow in lamellipodia and the wound healing response of epithelial cells is not affected by the tagged variants. They then apply the tagged actin to study subcellular distribution of different actin isoforms in mammalian and yeast cells.

The identification of a specific site in the actin protein that tolerates variable peptide insertions is very exciting and of fundamental interest for all research fields that deal with cytoskeletal rearrangements and cellular morphogenesis. The result demonstrating the functionality of actin variants with peptides inserted between aa 229 and 230 are generally convincing and well done. In particular, the generation of CRISPR/Cas9 genome edited versions of beta- and gamma actin are impressive. I therefore generally support publication of this study. There are however several technical and conceptual issues that should be addressed to improve quality and scope of the study. I listed some specific comments below:

We thank the Reviewer for their constructive comments and general support for publication of our study.

Major points

- The biggest issue I have is the last section on the application of tagged actins to study isoform functions. In principle the application is very clear as there are simply no alternative ways to study isoform distribution in live cells. However, the experimental data are simply not convincing. What the authors define as "cortex" in Fig. 5A seems to rather represent cytosolic background mixed with radial fibers. I am not convinced that even the antibody staining with a relatively clear differential distribution of beta and gamma really shows a genuine accumulation of one isoform on stress fibers. It seems to me that the beta-actin staining has as higher cytosolic background and is generally weaker (gamma nicely labels transverse arcs), which reduces signal/noise and therefore yields a relatively increased level in areas with less-bundled actin. My suggestion is to select more clearly defined actin structures and to use micro-patterned cells to normalize the otherwise obstructing variability in actin organization. Possible structures would be cortical arcs in bow-shaped cells, lamellipodial edges (HT1080 seem to make very nice and large lamellipodia) or cell-cell contacts (confluent monolayer, provided cells don´t grow on top of each other). Stress fibers are possible but need to be segmented very precisely and I did not see any details on this in the methods section. For Fig. 5D: I assume cells were used where only one isoform was tagged? This is technical weak and the double-normalization is probably blurring any difference that might be occurring. Why not use a double-tagging strategy with ALFA/FLAG or ALFA/AU5 tags to exploit the constructs introduced in the previous figures? Also, the unique selling point of the strategy is the possibility of actual live imaging of specific isoforms. Cells that have stably integrated double tags and then transiently express nanobodies for ALFA and either AU5 or FLAG (or other if those don't exist) would make this possible. Considering the work already done in this manuscript, such an approach should actually be possible - did the authors attempt this or is there are reason it is not discussed? If double tagged cells are not possible for some reason it should at the very least be possible to combine ALFA-detection with the specific antibody against the other isoform and get rid of the double normalization.

We thank the Reviewer for the various suggestions regarding the comparison between the localization of the tagged and native isoforms. In our reply below, we will separately discuss the possibilities and our considerations for fixed samples and live cell imaging. We apologize for the lengthy response but for transparency reasons, we would like to give a thorough overview of our efforts for isoform-specific localization in cells, something for which we have limited space in the manuscript.

Fixed samples:

It was a significant experimental challenge to compare the labeling of the β- and γ-actin specific antibodies with our internally tagged actin system (**Fig. 5A-D** in the original manuscript, now **Fig. 5B and Fig. S14**). The reason for this is that the labeling of the samples with the β- and γ-actin specific antibodies requires treatment with methanol (*Dugina et al., J Cell Sci, 2009*), most likely to disturb the interaction of actin with actin-binding proteins that prevent the binding of the antibodies due to steric hindrance. Methanol treatment, however, precludes the co-labeling with phalloidin, likely due to changes in the tertiary/quaternary protein structure of F-actin. Initially, we have put a lot of effort in trying to simultaneously label phalloidin with the actin specific antibodies but even very brief methanol treatment (seconds), before or after phalloidin labeling, completely prevents/reverses the binding of phalloidin. Importantly, also the ALFA tag labeling was suboptimal after methanol treatment.

The fact that we could not perform these double labelings led us to perform different ratio calculations for the β- and γ-actin antibody and the ALFA tag labeling. In the case of the antibody immunofluorescence labeling, we simply divided the signal of the β-actin and γ-actin since we could simultaneously label the isoforms in the same cell. In the case of the ALFA tag labeling, we used phalloidin for independent signal normalization and then performed a second normalization. Although this complicates the normalization procedure (ALFA tag signal of β- and γ-actin is first normalized to total F-actin and then a ratio is calculated) and understandably leads to some confusion, this was the only way forward to obtain the results presented in the manuscript.

The Reviewer points out that "*What the authors define as "cortex" in Fig. 5A seems to rather represent cytosolic background mixed with radial fibers.*". In our images, we observe very little cytosolic background from both antibody stainings. More importantly, for the quantitative analysis, the fluorescence intensity values were corrected for the background values observed in cytosolic areas so even if the signal is present, it should not affect our analysis. Nonetheless, we do agree with the Reviewer that the term "cortex" is misleading in this context since the observed signal could indeed be a mix of radial fibers and the actin cortex. We have therefore decided to remove these data and replace them by new experiments and quantifications (see details on Revisions below). The Reviewer further states that "*I am not convinced that even the antibody staining with a relatively clear differential distribution of beta and gamma really shows a genuine accumulation of one isoform on stress fibers.*" Although the differences are small, we consistently observe a differential fluorescence intensity of β- and γ-actin in actin-based structures (Original **Fig. 5C**). Since we always normalize the fluorescent signal intensity per cell, this strongly indicates a genuine accumulation of one isoform over the other for specific actin-based structures. This observation is very consistent across our experiments and also aligns with many published studies where differences in the localization of β- and γ-actin are reported for actin-based structures in a large variety of cell types (Pasquier et al., Vasc Cell, 2015; Chen et al., Nat Comms, 2017; van den Dries et al., Nat Comms, 2019; Malek et al., Int J Mol Sci, 2020; Chen et al., Nat Comms, 2021; Nietmann et al., Nat Comms, 2023). Importantly, also our new quantifications on stress fibers, lamellipodia and filopodia (see details on Revisions below) indicate a differential recruitment of β- and γ-actin to actin-based structures, further supporting the notion that there is a true difference in the localization of these cytosolic actin isoforms.

Revisions: Based on the Reviewers suggestion (and the suggestions from the other Reviewers), we have completely revised the original **Figure 5**. We have left out the data on the "cortex", due to the ambiguous interpretation of these regions of interest, and included β-/γ-actin ratio calculations for stress fibers, lamellipodia and filopodia. Moreover, we have performed our analysis on cells seeded on normal coverslips as well as on 2D crossbow-shaped micropatterns, as suggested by the Reviewer (the latter in collaboration with dr. Jeffrey van Haren who is now included in the author list). In these experiments, we first noted that on both substrates, HT1080 cells assembled a sufficient number of stress fibers, lamellipodia and filopodia to perform a thorough quantification. Moreover, based on the ratio calculations, we consistently observed higher β-/γ-actin ratios in filopodia and lamellipodia as

compared to stress fibers in both parental cells as well as the ALFA IntAct cells. This observation was consistent irrespective of whether we performed our analysis on cells seeded on glass coverslips or on 2D crossbow-shaped micropatterns. Notably, we further frequently observed that the β-/γ-actin ratio was even higher in filopodia as compared to lamellipodia but this observation was not consistent enough to reach significance in all conditions. The results of these immunofluorescence experiments and ratio calculations are now shown in the revised **Fig. 5A-C and Fig. S14** and associated text on **Page 15-16**. Altogether, we conclude that these new and more extensive analyses provide a strong support for the notion that ALFA-tagged β- and γ-actin behave similarly to the WT isoactins in HT1080 cells.

In addition to the newly added datasets, we have now also provided a more detailed explanation of the quantitative analysis of the β-/γ-actin ratio in the revised Methods section, including a simple equation that clarifies how the double normalization was performed (**Page 35-36** of the revised manuscript). Lastly, we have now also better highlighted how we performed the selection of the regions of interest for the different actin-based structures (**Page 35** of the revised manuscript).

<u>*Live cell imaging:*</u>
We agree with the Reviewer that it would be very valuable to attempt simultaneous live cell imaging of two isoforms. Yet, for this, we would need two tag/fluorophore systems that allow the visualization of internally tagged isoforms in living cells. As presented in our original manuscript, we have successfully inserted many different epitope tags (FLAG/AU1/AU5/ALFA) in the T229/A230 position to demonstrate the versatility of our tagging approach. Yet, despite significant efforts to identify a second tag/fluorophore system that would allow isoform-specific live cell imaging, we only succeeded in designing one strategy to perform live cell imaging, i.e. with the ALFA tag (Götzke, Nat Comms, 2019). Part of the reason for this is that so far, no high affinity nanobodies have been generated against the classical epitope tags (FLAG, AU5 etc.). This is an established challenge since classical epitope tags are typically linear/unstructured while nanobodies require folded secondary structures for epitope recognition such as alpha helices (the ALFA tag was specifically designed as such).

Besides the successful ALFA tag approach we have tried the following additional approaches for live cell imaging: ***1)*** full-length GFP, ***2)*** full-length GFP with linker, ***3)*** GFP11 (to complement with GFP1-10 (Cabantous et al., Nat Biotech, 2005) ***4)*** GFP11 with linker ***5)*** FLAG Frankenbodies (Zhao et al., Nat Comms, 2019; Liu et al., Genes Cells, 2021) in FLAG IntAct cells and ***6)*** Tetracysteine/FlAsH labeling. Importantly, each of these additional internally tagged actins, except for those that contained full-length GFP, showed a high colocalization with the cytoskeleton, again demonstrating the versatility of the T229/A230 position to tag actin. Unfortunately, none of these approaches satisfactorily visualized the actin isoforms in living cells. We will therefore briefly summarize our findings here.

(***1-2, integration of full-length GFP and GFP with linker***) Probably not surprisingly, but integrating the entire coding sequence of GFP or GFP flanked by linkers (each 5AA in length) within the T229/A230 position did not results in a proper localization of actin (**Reviewer Fig. 1**).

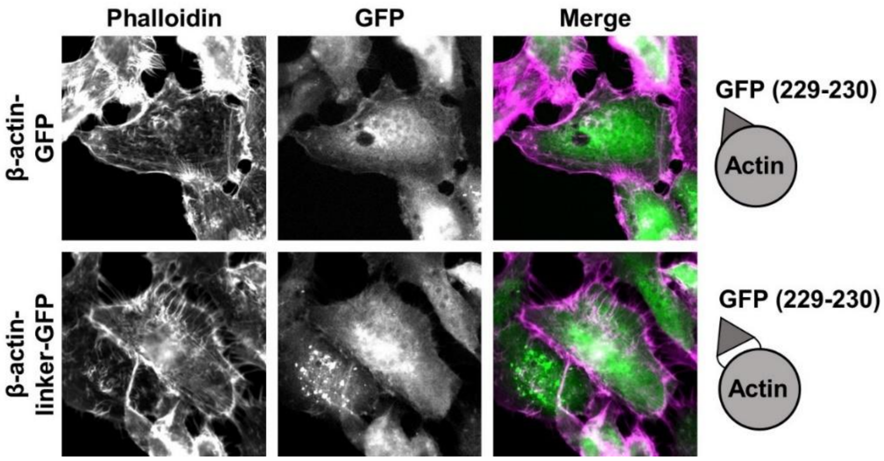

Reviewer Figure 1. Mislocalization of internally tagged GFP-actin.

(***3-4, integration of GFP11 and GFP11 with linker***) Next, we assessed the localization of the GFP11 tagged actin versions (GFP11: 16AA, GFP11+linker: 26AA). Because GFP11 is not visible without GFP1-10 complementation, we also tagged actin at the N-terminus simply for proof of concept where the internally tagged actins would end up. Interestingly, both GFP11-actin and GFP11+linker-actin properly integrated within the cytoskeleton as demonstrated by the FLAG staining (**Reviewer Fig. 2**). This again demonstrates the versatility of the T229/A230 position and strongly suggests that even the integration of ***26AA*** within this position does only minimally affect the polymerization of actin into the cytoskeleton.

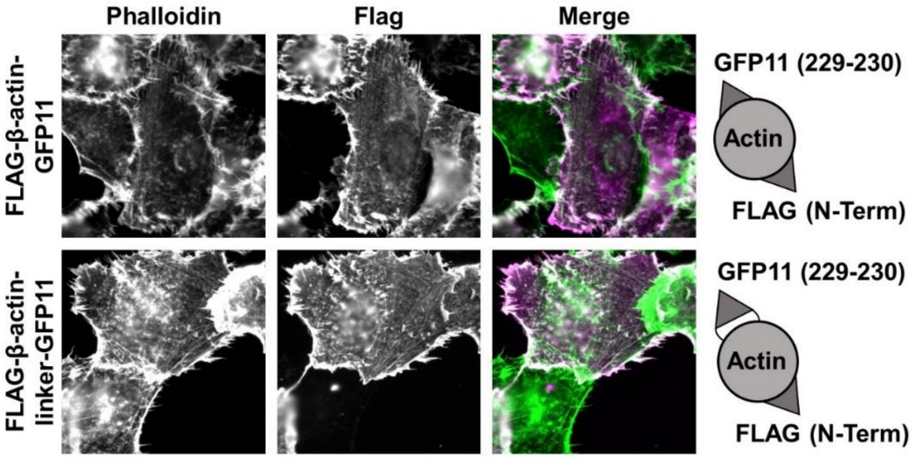

Reviewer Figure 2. Proper integration of GFP11-actin into the F-actin cytoskeleton.

(***3-4***) After confirmation of the proper integration of GP11-actin and GFP+linker-actin we continue to express the GFP1-10 in these cells. Unfortunately, this resulted in no or only very minimal localization of the actin to the cytoskeleton (**Reviewer Fig. 3**), demonstrating that GFP-complementation hampers the integration into the cytoskeleton.

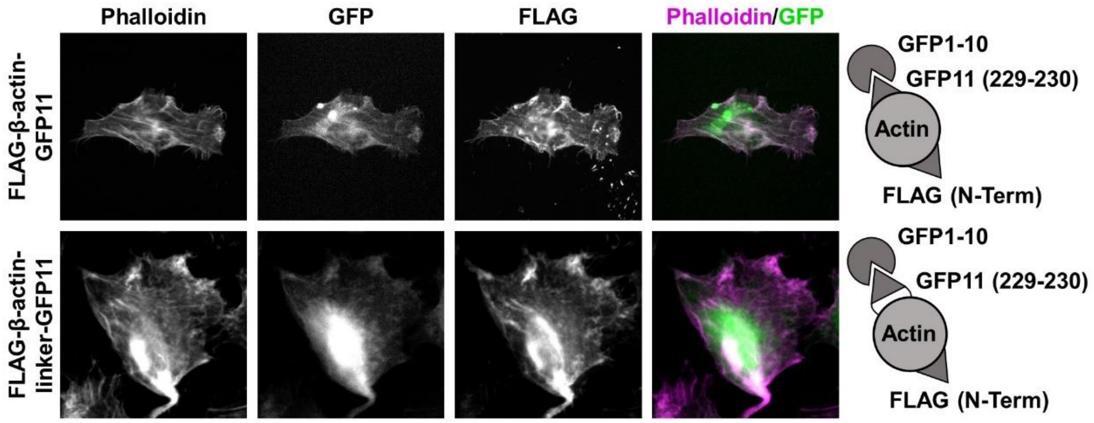

Reviewer Figure 3. Minimal colocalization between complemented GFP11-actin and phalloidin.

(***5, use of FLAG Frankenbodies***) We also expressed FLAG Frankenbodies into our FLAG IntAct cells in an attempt to visualize the isoforms in living cells. FLAG Frankenbodies are single chain antibodies fused to GFP and can be expressed in cells to visualize FLAG-tagged proteins (Liu et al., Genes Cells, 2021). Although a cytoskeletal labeling was indeed discernable in some cells, the FLAG Frankenbody signal overlapped much less with the total actin signal as compared to the FLAG immunofluorescence labeling, indicating that the incorporation of the FLAG-tagged actin was much less in the presence of the FLAG Frankenbody. Also, a significant fraction of the cells demonstrated a homogenous cytosolic signal (**Reviewer Fig. 4**).

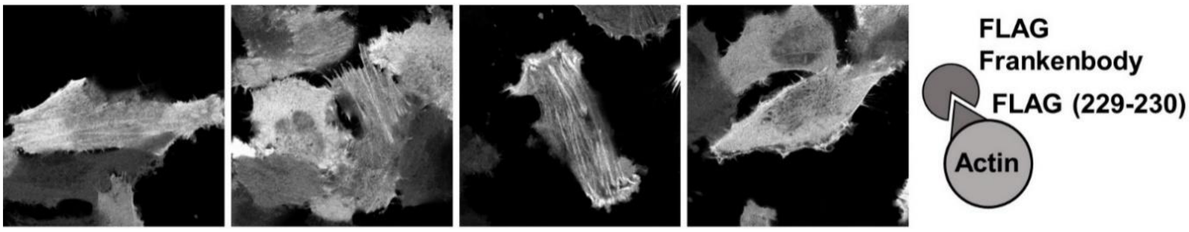

Reviewer Figure 4. Localization of FLAG Frankenbodies in FLAG IntAct cells

(***6, Use of tetracysteine/FlAsH***) Although the tetracysteine tag/FlAsH system is widely known to induce artefacts, we still aimed to evaluate if for live cell imaging of IntAct actins. Similar to GFP11, we first determined the integration of tetracysteine-actin into the cytoskeleton with the use of an additional N-terminal FLAG tag and demonstrate that it was properly integrated into the actin cytoskeleton (**Reviewer Fig. 5**). Unfortunately, after brief incubation with FlAsH-EDT2, we noted *1)* a significant amount of background fluorescence, preventing proper actin visualization (**Reviewer Fig. 6**) and *2)* that the cell became static indicating toxicity of the FlAsH-EDT2 compound. Titrating down the amount of FlAsH-EDT2 did not alleviate these drawbacks and only resulted in less fluorescence.

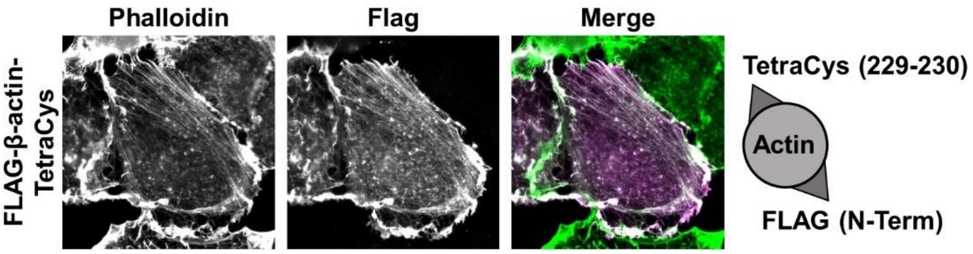

Reviewer Figure 5. Colocalization of TetraCys-actin with F-actin.

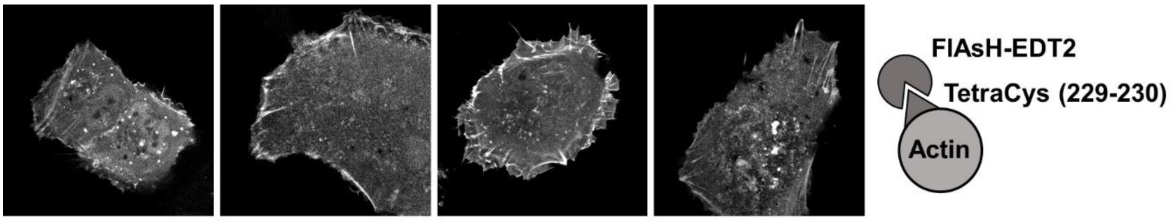

Reviewer Figure 6. Visualization of the TetraCys tag using FlAsH-EDT2.

Overall, based on these experiments, we concluded that the T229/A230 position itself is very versatile, as demonstrated by the proper localization of the GFP11-actin variants and the TetraCys-actin. At the same time, none of these tag/fluorophore systems properly visualized actin in living cells. Although we are unsure what the reason is for this, it is easily imaginable that the on/off kinetics of the split GFP system and the FLAG Frankenbodies are suboptimal to allow for the rapid and continuous integration of actin monomers into the F-actin cytoskeleton. We therefore also concluded that currently, the ALFA tag/nanobody system is apparently unique in its ability to visualize epitope tagged actin in living cells (as shown in the manuscript). For simultaneous visualization of multiple isoforms, we rely on progress on the development of novel nanobody-based tags, something we hope the Reviewer will agree is outside the scope of the current work.

- The authors make a point of comparing the internally tagged actin to N-terminal tags that are mostly functional but have been shown to affect translational efficiency. I would strongly suggest to include N-terminally tagged actin as control for all assays in this study. Also for the physiological assays (retrograde flow, wound healing), a positive control is missing that shows some effect. Previous studies showed defects with transiently expressed actin with an N-terminal GFP. As retrograde flow measurements are very sensitive to the exact position of the kymographs and wound healing assays is a very crude and indirect readout, such a positive control is essential.

For our studies, we specifically aimed to design an internally tagged actin with similar characteristics as wildtype (WT) actin. The decision to go for an internal position was made because the N-terminus has been shown to undergo functionally relevant post-translational modifications and the C-terminus is unavailable for tagging since it disrupts filament incorporation of actin. To initially determine the degree to which the internally tagged actins are integrated into actin-based structures, we have used the N-terminal control since this allowed us to compare and normalize the colocalization values of our newly tagged actins to a well-established control. For these overexpression experiments, we could not compare the tagged actins with WT actin because there would be no method to detect overexpressed WT from endogenous WT.

For the remaining of our manuscript, we have used CRISPR/Cas9 knockin (KI) cell lines. These KI cell lines allowed us to directly compare tagged actin with WT actin in different cell lines or even tagged actin protein with WT actin protein in the same cells in the case of heterozygous insertion. We have not included N-terminally tagged actin in these experiments, since such inclusion would not affect our conclusions with respect to the functionality of the IntAct actins as compared to WT actin. We do certainly expect that for future investigations into the importance of actin N-terminal modifications in

the differential regulation of actin isoforms, the comparison between IntAct actins and N-terminally tagged actins (either with GFP or with ALFA) could be very instrumental. Yet, this would require the generation of new KI cell lines with the ALFA tag at the N-terminus in the endogenous β-/γ-actin locus followed by an extensive comparison between N-terminally tagged and internally tagged actin. We consider this comparison outside the scope of the current manuscript. At present, the results in the manuscript provide strong evidence that our approach is unique with respect to the fact that it allows isoform-specific tagging without manipulating the N-terminus. As such, our internal tagging system complements the already existing repertoire of actin reporting methods (N-terminal fusion, Lifeact, F-Tractin, actin nanobodies) and allows researchers to study so far unknown properties of actin variants. We have now better highlighted the specific additional value of IntAct as compared to other probes in the discussion of the revised manuscript (**Page 24**).

- Expression of tagged actins in yeast is a very nice idea but it would be far more informative to express the tagged forms as the only copy of actin. This can either be done by directly replacing endogenous actin gene in S. cerevisiae, or (if the tagged versions are not viable) - using the established plasmid shuffle system (express actin on counter-selectable plasmid, then knock out endogenous copy and introduce additional plasmid with tagged actin, then force original plasmid out). In the presence of endogenous S. cerevisiae actin the shown effects are very hard to interpret as nothing is known about relative protein levels (endogenous vs. introduced). Also, if constitutive expression of the ALFA nanobody is harmful for integration into cables, why not perform inducible expression of the nanobody and observe labeling after induction. For the live imaging a robust cable marker is needed, like Abp140-GFP. Finally, indicate the sequence differences between the used actin forms in yeast (supplementary figure with sequence alignment and clear indication of all variations)

We thank the reviewer for their positive comments and feedback regarding expression of IntAct variants in yeast. In all our experiments, we have expressed IntAct variants as an extra copy under the constitutive expression of *TEF1* promoter in the presence of native *act1* of *S. cerevisiae*. pTEF is the promoter of the gene encoding for the translation elongation factor EF-1 alpha and is a strong constitutive promoter commonly used in budding yeast which would express IntAct at equal or likely higher levels as compared to endogenous actin as per estimates from previous studies (Thakur et al., Mol Cell Prot, 2011).

Revisions:

1. As per the Reviewer's suggestion, we have now attempted to generate budding yeast strains with IntAct as the sole expressing actin copy using the well-established 5'-FOA-based plasmid shuffle system. We have used a *Δact1* strain containing wildtype *act1* in a centromeric ura-plasmid described in *Harrer et. al*, 2007 (generously shared by Prof. Henty-Ridilla and Prof. Amberg at Upstate Medical University of New York, USA) and expressed IntAct exogenously via additional plasmids (low-copy and high-copy plasmids). Shuffling of these strains on media containing 5'-FOA causes the loss of ura-plasmid containing the wildtype *act1* copy and will determine whether yeast cells will be able to survive with IntAct as the sole source of actin. We performed the 5'-FOA shuffling experiment as a spot assay and unfortunately, our results showed that Sc-IntAct could not restore viability in the absence of native *act1*, while native *act1* under the same plasmid could restore viability as a positive control. The results of these experiments are now included as **Fig. S17A** and described on **Page 18** of the revised manuscript. Because of the unsuccessful actin substitution experiments, we were unable to test the effects of IntAct as a sole copy of actin in yeast. Based on previous studies with yeast actin and the known strict structural requirements for formin-based incorporation of actin subunits (Chen et al., J Struct Biol, 2012), we were aware that substituting native actin with tagged actin would be a significant challenge and this will require additional directed efforts towards optimization in the future.

2. As the reviewer rightly mentioned, expression of ALFA-Nb-mNG during live-cell imaging experiments hindered incorporation of IntAct into linear actin cables in yeast (**Fig. S18**) and as per the Reviewer's suggestion, we have now created an inducible expression system for the ALFA-Nb-mNG under the GALS promoter and observed its effects on incorporation into formin-made actin cables after induction. We added galactose to a final concentration of 2% and imaged the cells after 2 hrs of galactose addition. Faint ALFA-Nb-mNG signal was observed during imaging which could be observed only at very high laser intensities confirming that ALFA-Nb levels were very low. We acquired 3D-stacks of cells but only observed signal resembling actin patches and no actin cables (**Fig. S22** and text on **Page 19**). This may suggest that even very low amounts of ALFA-Nb-mNG could interfere with incorporation of IntAct into formin-made cables or incorporated IntAct may not be accessible to ALFA-Nb binding in living cells due to steric hindrance from other actin cable-binding proteins. Future studies, possibly including the use of ALFA-Nbs with a high dissociation rate (Zhang et al., Cell Rep Meth, 2022), will shed more light on the mechanisms underlying this possible exclusion or hindrance and provides indicatations for how to improve the utility of IntAct to visualize actin cables. These considerations are now added to the discussion of the revised manuscript on **Page 23**.

3. We have now performed live-cell imaging of Sc-IntAct with the actin patch marker protein Abp1 C-terminally tagged with 3xmCherry at its native locus (**Fig. S21A**). Time-lapse movies showed co-localization with Abp1 at actin patches which represent sites of endocytosis in yeast (Winter et al., Curr Biol, 1997; Young et al., J Cell Biol, 2004). This allowed us to quantitatively compare the effect of IntAct expression on actin patch lifetime which is defined as the time window between the appearance and disappearance of a patch. Using Abp1-3xmCherry as an actin patch marker, we compared actin patch lifetimes between *wildtype* cells and cells expressing *act1* or Sc-IntAct with/without ALFA-Nb-mNG co-expression. The results are shown in **Fig. S21B** (text on **Page 19**) and demonstrate that IntAct expression does not affect actin patch lifetime. These observations demonstrate the potential of using IntAct as a direct marker for actin dynamics at yeast actin patches, complementing existing surrogate markers such as Abp1 or Arc15 (Goode et al., Genetics, 2015; Wirshing et al., J Cell Biol, 2023) and also support its non-disruptive nature.

4. We have now included a multiple sequence alignment of all IntAct variants expressed in yeast in supplementary **Fig. S15**.

5. Additionally, we have now also performed experiments with expression of IntAct variants in *S. pombe* as suggested by ***Reviewer 2*** and the results of these experiments are presented in **Fig. 5E, Fig. S18 and Fig. 23B** in the revised manuscript. All IntAct variants in *S. pombe* were expressed under the control of *S. pombe act1* promoter from an exogenous *ura*-based plasmid. We kindly refer to our answer to ***Reviewer 2*** (**Page 16-17** of this response letter) for more details on our observations from IntAct expression in *S. pombe.*

- As the authors clearly show good integration of several tagged actins into filaments I would expand the structural characterization: perform alpha fold predictions of actin monomer structures including the various tags to show the expected orientation. It is striking that the only integration site that seems to work well is at the last position of a short helix, indicating that the orientation of the integrated peptide might be fixed in space and be optimal to minimize interference. Also, a docking of the tag onto the recently published cryoEM structures of the actin filament should be shown to indicate where it resides compared to tropomyosin or the major groove where most side binding proteins seem to bind.
We already performed AlphaFold predictions of the tagged actin monomers (**Reviewer Fig. 7A-C**), but decided to not include these predictions in the manuscript because of two reasons. First and foremost, while the prediction confidence of the non-tagged region is very high (pLDDT > 90), the prediction confidence of the tagged region is very low (pLDDT < 50 for both FLAG and the ALFA tag). As indicated

on the AlphaFold website (https://alphafold.ebi.ac.uk/faq), pLDDT values below 70 should be treated with caution and values below 50 should not be used for interpretation. Intriguingly, the low confidence aligns with the fact that for both tags, the prediction does not match with known features of the tag. The FLAG tag should be a linear/unstructured region in order to be recognized by the antibody and the ALFA tag should organize into an alpha helix (Götzke et al., Nat Comms, 2019). Yet, in the prediction, the FLAG tag partially continues as an alpha helix and the ALFA tag is only a small helix with part of the tag being unstructured. Second, more minor, reason for not including the predictions is that AlphaFold does not predict to what extend the tag is flexible, which means that even if the tagged region is predicted correctly, it is difficult to say whether the regions will interfere with binding of proteins.

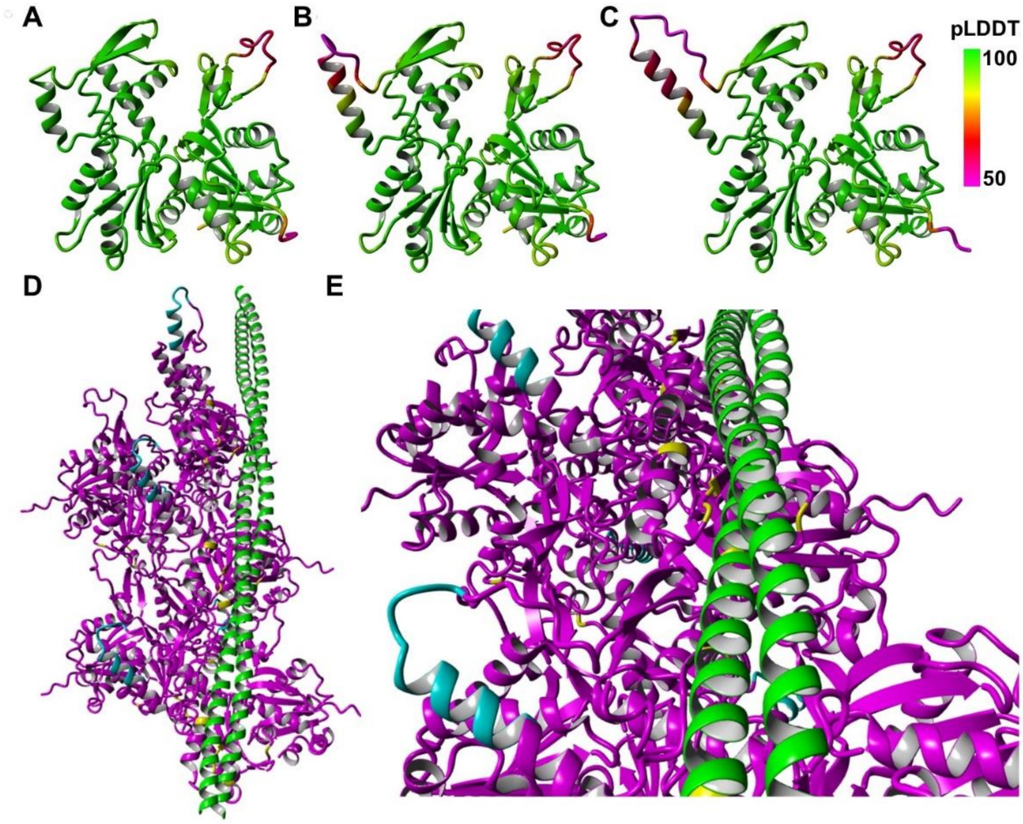

Reviewer Figure 7. Predicted structure of tagged actins and overlay with actin-tropomyosin complex.
A-C) AlphaFold predictions of WT actin (A), FLAG IntAct actin (B) and ALFA IntAct actin (C). The color indicates the pLDDT score which is a measure for the prediction confidence. Besides the D-loop, the prediction of the tagged region is very low and is difficult to interpret based on the prediction. **D-E)** Two views of the cryoEM structure of the F-actin-tropomyosin complex (PDB: 3J8A) with the WT actin monomers being replaced for ALFA IntAct actin. Actin is colored magenta with the ALFA tag in cyan and the residues that facilitate interaction with tropomyosin in yellow. Tropomyosin is colored green.

Despite the low prediction confidence, we used the published actin-tropomyosin cryoEM structure (von der Ecken et al., Nature, 2015) to replace WT actin with ALFA tag actin and the results are shown in **Reviewer Fig. 7D-E**. Again, although results should be interpreted with caution, the tag does not seem to obstruct monomer-monomer interactions within an F-actin filament and also the tropomyosin binding surface is relatively distant from the tag region, suggesting that these interactions are likely not disturbed by introducing the tag.

- For any claims regarding usability of tagged variants for isoform research it would be very important to characterize the known posttranslational modifications of tagged actin variants - are the differences between beta and gamma maintained on this level as well?

Revision: We have now performed a western blot to compare the arginylation of WT and IntAct actins using an antibody that specifically recognizes arginylated β-actin and the results are shown in **Fig. S6A** (Text on **Page 8**). Based on this analysis, we conclude that there are no differences in β-actin arginylation between WT and tagged actins.

Technical issues

- There is no scale for the color coding in Fig. 5A, B

We deliberately did not add a numerical scale because the images are normalized which means that presenting the actual numbers might be misleading. The numbers could be interpreted as if they actually present the amount of β-actin relative to γ-actin which is not the case due to staining differences and the normalization procedure. Since the affinities of the β- and γ-actin antibodies are different, it has so far been impossible to reliably estimate the proportion of β- vs γ-actin in actin filaments. We hope that the revised **Fig. 5** and **Fig. S14** now better demonstrate the observed differences between the localization of β- and γ-actin in parental and IntAct cells.

- The y-scales for Fig. 5C and D need to be identical to allow direct comparison

Revision: In revising **Fig. 5** (see response to the first comment of this Reviewer on **Page 2-3**), we have also included identical y-scales for all of the graphs.

- Pearson coefficient should not be normalized to a control value as its already a dimensionless parameter. Always report actual R-value - also remove R2 values for Pearson as this makes no sense in this context (not sure if it was a typo or intended).

We normalized the Pearson coefficient values for visual representation of the results. The majority of the raw coefficient values (more than 80%) are between 0.20 and 0.75 (see raw values in the associated excel file). Theoretically, Pearson coefficient values are possible between 1 (or-1 for negative correlations) and 0. The much smaller window in our values as compared to the theoretical window (0.55 vs 1) led us to normalize the values such that they can be presented on a scale from "maximum expected colocalization" to "minimum expected colocalization". In this way, the differences between the various tagged actins are much better appreciated in the Figure. Importantly, in the Source Data file that will be accompanied with the manuscript, we do list the raw R-values for transparency.

Revision: We have now better explained the normalization procedure in the Methods section of the revised manuscript (**Page 35**). As to reporting the R^2, the Reviewer is correct. Reporting the R2 is an inadvertent mistake from our side and we have now changed the R^2 in the text to PCC or Pearson Correlation Coefficient (**Page 4 and Page 10** of the revised manuscript).

- All values on subcellular regions (like stress fiber or cortex) dependet critically on the way thesese regions were thresholded or identified. Provide all details on how this was done in the methods section and ensure that adequate background subtraction and normalization is applied. Optimally, an unbiased (AI or automated) approach based on simple image statistics is used for this to avoid personal bias.

Revision: As also mentioned above, while revising **Figure 5** we have now provided a more detailed explanation of how the background subtraction and the selection of the regions of interest was performed (**Page 35-36** of the revised manuscript).

- In Fig. 2A only heterozygous FLAG-actin cells are used. Why not use a homozygous line (for both beta and gamma actin)? The nice band shift of the FLAG version would allow the precise quantification of the fraction of total actin covered by beta and gamma actin, which then could provide some additional

info for the apparently weaker beta staining in Fig. 5 (if beta expression is simply weaker). This would be a very simple and useful advantage of the internal tags that could be widely applied.

In **Fig. 2A-B**, we used the heterozygous FLAG-actin cells to directly compare the production of β-actin from the knock-in allele and the wildtype allele in the same cells. The fact that the two bands observed in this western blot analysis (upper and lower) are almost identical (with the FLAG band being a bit more intense) provides a strong indication that the tag does not interfere with the expression of actin. Moreover, in our original manuscript (original **Fig. S5D**, now **S4D**), we already demonstrated that the expression of β-actin is also unaffected in the hemizygous FLAG actin cells, which exclusively express tagged actin.

Revision: As per the Reviewer's suggestion, we have now also added a western blot analysis on the expression of both actin isoforms and total actin in hemizygous cells, which are shown in **Fig. S5B-C** of the revised manuscript (Text on **Page 7**). This showed that the expression of the tagged actins is comparable to wildtype actin. As also suggested by the Reviewer, we have now assessed the fraction of total actin covered by β-actin and γ-actin. This showed that β-actin covered ~55% of total actin and γ-actin covered ~45% of total actin (**Reviewer Fig. 8A-B**). Although to us, this indicated that possible differences in the immunofluorescence staining in Figure 5 are not the result of much lower expression of β-actin, we decided to only include these data here for the Reviewer since in our work, we are primarily interested in comparing the expression of the tagged and WT actin and want to avoid making strong claims about the respective fractions of each isoform, since we do not have a control in parental cells to confirm if the estimates of the fractions are correct. We do agree, however, with the Reviewer that for future investigations of actin isoforms, this could be an interesting option.

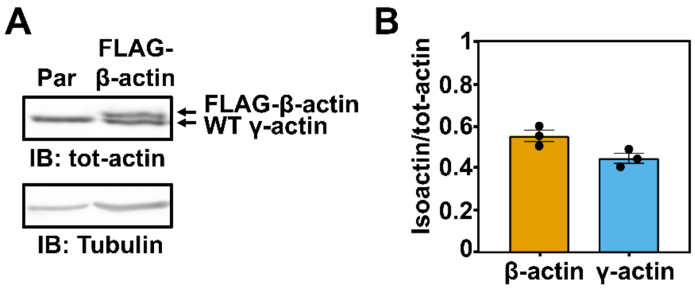

Reviewer Figure 8. Western blot analysis of total actin fraction covered by actin isoforms. (**A**) Representative western blot showing the expression of total actin and tubulin in parental and hemizygous FLAG-β-actin cells. (**B**) Quantification of total actin fraction covered by actin isoforms.

- Fig. 3: control with N-terminal tag is missing. Also, why is it not possible to assay filament binding factors like Myosin, Filamin or alpha actinin - instead of co-IP a simple co-sedimentation assay with cell extracts in F-buffer should pick up any major difference in decoration of filaments containing the ALFA tag. Using two speeds for centrifugation it might even be possible to observe effects on filament bundling. The best approach for this would of course be to purify tagged actins and perform in vitro assays but this is clearly beyond the scope of what the authors intended here. I personally think that a broad acceptance of the marker will only come once the biochemistry has been sufficiently characterized so this is a future direction I would strongly encourage.

We kindly refer to our response on **Page 6-7** for why we have not included the N-terminal control.

Revision: The co-sedimentation assay was an excellent suggestion by the reviewer. Following this suggestion, we have now performed F/G-actin fractionation and determined the presence of the F-actin associated proteins myosin IIA and α-actinin in the F-actin fraction (revised **Fig. S11**, text on **Page 11**). This demonstrated that myosin IIA and α-actinin were present in similar amounts in the F-actin fraction between parental and tagged cells. These findings aligns well with the comparable functionality of parental and tagged cells.

- Fig. 2A has no loading control

We show this western blot to indicate that the WT actin and tagged actin are expressed at similar levels in the heterozygous knock-in cells. For this, no loading control is needed because we only compare the intensity of the upper band (tagged actin) with the lower band (WT actin).

- The RPE-1 data are confusing as several constructs show very different localization (completely cytosolic) to HT1080 cells and there is no possible explanation given for this. Maybe simply remove this data set?

We agree with the reviewer that the differences in the localization between some of the internally tagged actins between the HT1080 and RPE1 cells might be confusing, especially for the A230-A231 variant for example. Yet, the fact that for both cell types, the T229-A230 variant performs the best is an important confirmation that this variant is properly integrated into actin-based structures, independent of cell type. This makes the support for choosing this variant to continue with our studies stronger. A possible explanation for the differences in colocalization that we observe is that RPE1 cells form more stress fibers as compared to HT1080 cells. Since the localization to stress fibers is different between the internally tagged actins, this may explain the differences observed in colocalization.

Revision: We have now added a few sentences in the Discussion (**Page 22** of the revised manuscript) on the differences between the colocalization values between HT1080 and RPE1 cells.

- The angel measurements for lamellipodial actin is not very meaningful: the angel is determined for the radial bundles, which do not correspond to the Arp2/3 angel of single filaments and is likely the results of different nucleation factors, I would suggest to remove this. If angel measurement are really intended, cryoEM needs to be performed.

We apologize for this misapprehension from our side which is also noted by the other two reviewers. In the treadmilling videos of the lamellipodia in HT1080 cells, which were obtained using Airyscan super-resolution microscopy, we clearly observe a consistent filament formation at a constant angle, something which we interpreted as the angle between the mother filament and the daughter filament. After consulting the literature, we indeed have to admit that this cannot be interpreted as such and we will remove these datasets.

Planned revision: We have now removed the datasets with the angle measurements from our manuscript.

- Replace all SEM with SD values - use at least 3 biological replicates (4D SEM of n=2)

Revision: We agree with the Reviewer that the SEM was not always appropriate. Therefore, we have carefully checked all of our statistics and revised where necessary. All statistical information is included in the Figure legends and a Source Data file is included for full transparency.

Minor points

- Intro: after listing all the details already understood on actin isoforms it is not very convincing to simply state the molecular principles remain largely unclear (l 34) - maybe better "there is no way to study actin dynamics due to current limitations of specific antibodies to fixed samples. Interesting option would be actually to develop nanobodies that are isoform specific.

We have now rephrased the text in the introduction (**Page 2** of the revised manuscript). Regarding the development isoform-specific nanobodies. Although this sounds like a promising way forward, this would likely not result in isoform-specific targeting in living cells. Similar to the antibodies, isoform-specific nanobodies would have to be generated against the N-terminus which, under native conditions, is likely not available due to the occupation with actin-binding protein. Also, since the N-terminus is not structured, it may be extremely challenging to generate nanobodies against these epitopes.

- L 71: "involved" in the kinetics is not a good term - maybe affects or regulates....
We have rephrased the text (**Page 4** of the revised manuscript).

- L148: "suspect" instead of "expect" - this clonal variation is actually a big danger of the employed approach as possible defects in actin organization could be masked by compensatory changes - it would generally be good to show critical data for at least 3 independent clones to rule out dominant selection effects.
We have rephrased "expect" to "suspect" (**Page 8** of the revised manuscript). We agree that clonal variation could be a danger if actin levels are to be investigated. For future follow-up studies, we plan to make additional cell lines to avoid clone-specific conclusions.

Referees cross-commenting
I completely agree with the comments by reviewer 2 on the various missing controls - adding several or all of those will make the results much more convincing. The key for the adaptation of any new actin probe will be the level of confidence researchers have on the doumented effects. Even some negative effects on actin behavior (I am sure there will be some) should not prevent usage of the strategy as long as there is robust and convincing documentation of those effects. I also agree that including some basic in vitro characterization will go a long way to convince people dierectly working on actin (there is a very high level of biochemical understanding in that field).
Revision: We have now performed the essential controls as suggested by ***Reviewer 2***. Furthermore, for future experiments, we do envisage the production and purification of internally tagged actins and investigate their binding properties in *in vitro* reconstitution assays. We have already started with optimizing these approaches through our ongoing collaboration (KD, SP).

Reviewer #1 (Significance (Required)):
Significance: Very useful finding that can be applied to any question related to actin-dependent cellular processes (morphogenesis, cell division, cell polarization, cell migration etc.)
Strength: main finding convincing, strong genome edited cell lines
Limitations: application to study of isoforms very limited and data not convincing, statistics and image quantifications need improvement
Advance: identify new location for integral tagging of actin, which was not really possible before. The main relevance is for fundamental cell biology but the approach can also be applied to the study of disease variants in actin.
Audience: general cell biology - very broad interest

Reviewer #2 (Evidence, reproducibility and clarity (Required)):

Actin is highly sensitive to modifications, and tagging it with fluorescent proteins or even smaller motifs can affect its function. The most well-known example of this is that fission yeast where actin has been replaced with GFP-actin are inviable (Wu and Pollard, Science 2005) because the labeled actin cannot incorporate into the formin-dependent filaments that make up the cytokinetic ring. Subsequent experiments revealed that formins filter out GFP-actin monomers, as well as monomers that are labeled with smaller fluorescent motifs (Chen et al, J. Structural Biology 2012). Further, attempts to make mammalian cells lines where GFP-beta-actin was knocked into one allele resulted in extreme down-regulation of the GFP-labeled actin, indicating that there is some implicit toxicity with the labeled version. To my knowledge, all attempts at making homozygous GFP-actin knock-ins have been unsuccessful. Therefore, while GFP-actin or other labeled variants can be over-expressed in many different cell types with some success, there is always the question of how faithful the labeled actin represents bona fide actin localization and dynamics.

To address this van Zwam et al. have developed a clever strategy of screening actin for internal motifs that can tolerate incorporation of a tag without affecting its function. They appear to have found a good candidate, named IntAct, and provide evidence that this tagging position allows the actin to be functional in both human and yeast cells. The work is very promising, and many of the assays performed satisfy the criteria of rigor and reproducibility. Importantly, the authors have created knock-in human cell lines where the tagged actin is expressed at normal levels, including a double allele knock-in that is viable and has normal proliferation and motility. Additionally, the authors show that labeled S. cerevisiae actin can incorporate into actin cables, which are formin dependent. IntAct constructs were shown to interact with several well-known actin binding proteins and localized well to many different actin structures. There was also interesting data obtained from tagging both beta and gamma actin in human cells. However, as an actin scientist eager for new probes to visualize actin in cells, there are still questions about the functionality of these probes. Addressing these issues, listed below, would alleviate the concerns I still have about IntActs after going through the manuscript. IntActs have the potential to have a large impact on cytoskeletal research if it can be rigorously documented that they are functionally as close to unlabeled actin as possible.

We thank the Reviewer for their constructive comments and general positive evaluation of our study.

Reviewer #2 (Significance (Required)):

Concerns:

1. There are no negative controls performed for either the fixed or live-cell imaging of IntAct. Since the fixed cell data is heavily reliant on the presence of flag-labeled puncta at actin filaments, it is important to show that the immunocytochemistry protocol doesn't produce anything that would mimic the localization of actin. For the live cell data, there has been no effort made to show that the binding of the nanobody to the ALFA tag on InAct is specific.

Revision: The Reviewer is raising an important, which is acknowledged by ***Reviewer 1***. We have now included the following controls in the manuscript: ***1)*** Expression of ALFA-Nb-GFP and ALFA-Nb-mScarlet in cells that do not have tagged actins and ***2)*** Expression of free GFP in cells that have tagged actins. In these experiments, the localization of the fluorescent proteins is clearly cytosolic under all conditions and does not mimic the localization of actin in any way. This demonstrates that the localization of the ALFA-Nb-GFP and ALFA-Nb-mScarlet to the F-actin cytoskeleton in the IntAct cells is specific. The results of these experiments are now included as **Fig. S9** of the revised manuscript (Text on **Page 10**)

Furthermore, we here also share the immunofluorescence staining of the FLAG tag and the ALFA tag in cells that do not express any tag (**Reviewer Figure 9**). For this, we used images from the experiments in which we transiently expressed the tagged actins or the pools of CRISPR/Cas9 knockin cells, since in these samples, the cells that express the tagged actin frequently neighbour cells that do not express the tag and therefore function as a direct control for the immunolabeling in the same sample. As can be seen below, no or very low signal is observed for both the FLAG labeling as well as

the ALFA labeling and we therefore concluded that aspecific labeling of the antibody or nanobody does not result in anything that mimics the localization of actin.

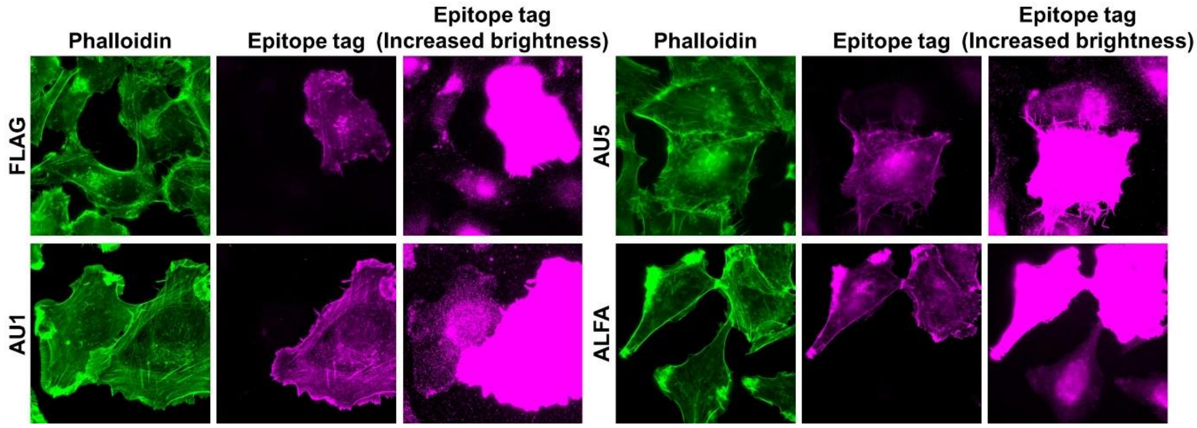

Reviewer Figure 9. Negative controls for immunofluorescence. Depicted are widefield images of cells that express or not express an actin variant with the indicated epitope tag. Under all conditions, very low or no signal of antibody or nanobody fluorescence staining is detected in cells that do not express the tag.

2. The homozygous ALFA-tagged IntAct cells have a 50% reduction in the amount of actin expression (Fig. 2D). What is the F:G ratio in these cells? The F:G measurement is only shown for the FLAG-tagged heterozygous IntAct cells, which have the worst co-localization with phalloidin (Fig. 2F) and were not used for subsequent figures. I appreciate that motility and proliferation were measured and shown to not be affected (Fig. 4D,E) , but in our lab reducing the amount of polymerized actin by 50% (which may be more in ALFA-tagged IntAct cells if the F:G changes) has catastrophic effects on other cytoskeletal and organelle systems. Since the homozygous ALFA IntAct cells are the main ones used in the manuscript, they should be the ones that are fully characterized.

The reduction in actin expression in the homozygous ALFA-tagged IntAct cells is only 25-30 percent depending on the specific western blot analysis and the loading control. Still, the Reviewer is correct about the necessity of the F/G-actin measurements of the ALFA-tagged IntAct cells and we therefore already included those as **Fig. S10** (also **Fig. S10** in the revised manuscript) in the original manuscript (text on **Page 10)**. The quantification of these assays clearly demonstrated that the F/G-actin ratio in the ALFA-tagged IntAct cells is the same as in parental cells. In addition, we would like to point out that we now also performed the F/G-actin measurements in the present of the ALFA-Nb-GFP and concluded that the stable expression of the nanobody does not affect the F/G-actin ratio (**Fig S10** and text on **Page 10**)

3. It is not addressed if expressing the ALFA-Nb-GFP construct in ALFA-IntAct cells alter actin properties? This is essential information for live cell imaging experiments.

Revision: We have now performed proliferation and migration experiments (Revised **Fig. S12**, text on **Page 13**), as well as F/G-actin measurements (Revised **Fig. S10**, text on **Page 10**), in cells that stably express the ALFA-Nb-GFP. From these experiments, we concluded that proliferation, migration and F/G-actin ratios are not affected by the presence of the nanobody. To note, in the original manuscript, we already showed that treadmilling of actin at the lamellipodia is not affected by the presence of the ALFA-Nb-GFP.

4. It is not addressed how much of the ALFA-IntAct gets labeled with ALFA-Nb-GFP and how uniform the labelling.

We do not understand this comment of the Reviewer. To our knowledge, it is not possible to assess how much of a probe (in this case the ALFA-Nb-GFP) binds the target (in this case the ALFA-IntAct actins)

in living cells. This is not only the case for the ALFA-Nb-GFP but also for any other probe. As an example, when expressing Lifeact, we also cannot assess how much of the actin molecules within F-actin are labeled with Lifeact or how uniform the labeling is. From our live-cell imaging experiments, we can only conclude that the binding is at least so effective that we can readily observe and discern all the actin-based structures that are also observed by Lifeact (see **Fig. S8** for Lifeact-GFP/ALFA-Nb-mScarlet cotransfection). Whether the cytosolic ALFA-Nb-GFP is largely free or bound to actin monomers seems an issue that cannot be addressed.

5. To assess lamellapodia architecture, "branched actin angle" is measured using AiryScan imaging of actin filaments. This type of microscopy does not offer the ability to image individual actin filaments; what is actually being measured is the orientation of actin bundles to each other. It should be impossible to image the orientation of actin filaments in Arp2/3 dendritic networks and it is surprising that the measurements average to 70 degrees. A suitable substitute for this would be to measure the size and amount of F-actin in phalloidin-stained lamellipodia using kymograph analysis.
We apologize for this misapprehension from our side which is also noted by the other two reviewers. In the treadmilling videos of the lamellipodia in HT1080 cells, which were obtained using Airyscan super-resolution microscopy, we clearly observe a consistent filament formation at a constant angle, something which we interpreted as the angle between the mother filament and the daughter filament. After consulting the literature, we indeed have to admit that this cannot be interpreted we have now removed these datasets.

6. Was it possible to make an IntAct gene substitution in yeast?
We thank the Reviewer for this important suggestion. As also suggested by ***Reviewer 1***, we have now tried to make yeast strains with IntAct as sole actin copy using the well-established 5’-FOA plasmid shuffle system. Unfortunately, our experiments suggest that IntAct cannot restore viability in the absence of native actin, the results of which are now included as **Fig. S17A** in the revised manuscript. We kindly refer to our answer to ***Reviewer 1*** (**Page 7-8** of this response letter) for a detailed explanation of our approach.

Also, while this is not necessary for this manuscript, making a fission yeast strain where actin has been substituted with IntAct and demonstrating that IntAct gets incorporated into the cytoplasmic ring and into Cdc12p-polymerized filaments would alleviate MANY potential concerns people would have about these probes by directly assessing situations were other labeled actins have been documented to fail. Along the same lines, it would have been nice to see a comparison in some of the assays of ALFA-IntAct and GFP-actin or another labeled actin variant.
We thank the Reviewer for their constructive feedback and completely agree that it is important to document how IntAct behaves in scenarios where other labelled actins have failed. As a proof of principle, IntAct incorporates into both formin- and Arp2/3- made linear and branched actin filaments in yeast (**Fig. 5D, Fig. S18 and Fig. S19**) and this data shows that IntAct labelling strategy is the first to achieve good integration into both these structures as previous efforts with labelled actin such as GFP-actin fail to incorporate into formin-made actin filaments (Doyle et al., PNAS, 1996). While this indicates that IntAct does perform better than other labelled actins in yeast, further optimizations are required to overcome limitations regarding incorporation into actin cables in the presence of the ALFA nanobody for live-cell imaging.

Revisions:
1. As per the Reviewer’s suggestion, we have now extended the applicability of IntAct to the fission yeast *Schizosaccharomyces pombe* (*S. pombe*). Fo this, we first assessed IntAct incorporation into fission yeast actin cables, patches, and rings in the absence of the ALFA-Nb-mNG. We expressed IntAct variants of human β- and γ-actin, budding yeast actin (Sc-IntAct) and fission yeast actin (Sp-IntAct) from

an exogenous plasmid under the native *S. pombe* actin promoter in a *wildtype S. pombe* strain expressing Lifeact-mCherry. The cells were then fixed and stained for IntAct using ALFA-Nb-Alexa647 to assess IntAct localization. Interestingly, similar to our observations in budding yeast, we observed that IntAct actins show a variant-specific incorporation into actin structures. β-IntAct, γ-IntAct, and Sc-IntAct only displayed consistent incorporation into the branched actin patches (**Fig. 5E and Fig. S23**), while Sp-IntAct was clearly observable in all three actin structures (i.e. cables, patches and the actomyosin ring) (**Fig. 5E**). These observations further emphasize the permissibility of the T229/A230 position for internal tagging of actin and represents a major advance towards direct actin visualization as previous tagged actins (GFP-actin) failed to incorporate into actin cables and specifically actomyosin rings made by the formins in *S. pombe* and *S. cerevisiae* (Okreglak et al., J Cell Biol, 2007; Wu et al., Science, 2005).

In addition to the experiments in fixed cells, we also expressed IntAct actins in a *S. pombe* strain that constitutively expresses the ALFA-Nb-mNG. Again, similar to our observations in *S. cerevisiae*, we observed that all IntAct variants only localize to actin patches located at the cell poles and cell division site (during cytokinesis), but that actin cables and rings were not detected (**Fig. S20**). The underlying mechanism for this exclusion from linear actin cable network from both budding and fission yeast remains unknown and may represent an inherent specificity and sensitivity of yeast formins (Chen et al., J Struct Biol, 2012). We have rephrased several sentences in the discussion of our manuscript (**Page 23**) to better emphasize this limitation. To improve our IntAct system for live cell imaging of all actin-based structures in yeast, we are fully committed to direct future efforts to extend and optimize the use of tags and fluorescent probes in *S. pombe* and *S. cerevisiae*. We, however, consider this outside the scope of the current manuscript.

2. To address the applicability and effects of IntAct expression on normal actin dynamics in yeast, we also compared actin patch lifetimes between *wildtype* cells and cells expressing Sc-IntAct in *Saccharomyces cerevisiae*. Our results show that IntAct expression did not affect actin patch lifetime as compared to *wildtype*, either in the absence or presence of the ALFA-Nb-mNG, something which is now included as **Fig. S21** in the revised manuscript. We kindly refer to our answer to ***Reviewer 1*** on **Page 8** of this response letter for a detailed explanation of our approach.

Overall, our results demonstrate the applicability and future potential of IntAct as a tool to study actin isoform function across model organisms.

Reviewer #3 (Evidence, reproducibility and clarity (Required)):

Summary:

This paper tackles a new strategy to tag actin in cells, by identifying that incorporation of a tag of moderate size in subdomain 4 of actin minimally affects actin dynamics in cells, and does not perturb its interaction with known partners, as observed in pull-down assays.

Major comments:

The paper is interesting and experiments are convincing.

My main concerns are the following :

- Varland et al, is reporting a phosphorylation on Thr229 : I think the authors should mention and discuss this potential PTM that could be affected in IntAct.

We thank the Reviewer for pointing this out. We are aware of this review that includes phosphorylation on Thr229 as a possible PTM. Yet, this PTM is only reported in one of the Tables of the Review and not further discussed in the text or supported by references to primary research articles. It is further unclear how the authors determined that Thr229 is a possible phosphorylation site except for the fact that this residue is a threonine and exposed at the surface of the actin molecule. Together with the fact that there is no evidence from primary studies that Thr229 is phosphorylated, we therefore decided to not include it in our discussion.

- The sequence in subdomain 4 (the alpha helix containing T229A230) is extremely conserved in animals, as well as in between the 6 human actin isoforms. This usually indicates a strong selection pressure on the residues. I think the authors should discuss how surprising it is that the T229A230 position can accomodate various tags while it is probably the place of interaction with other proteins and is playing an important role in the mechanical structural integrity of the actin itself.

We thank the Reviewer for bringing up this important point. To a certain extent, the conservation argument is true for all of the residues/domains in actin. Any manipulation will change a conserved part of the actin molecule in one way or another and thereby potentially modify its function. This is also evident from the fact that for most of the internally tagged actins, we observed a very poor colocalization with the actin cytoskeleton (**Fig. 1**). To emphasize this point in the original manuscript, we had therefore already included in the discussion that, at this point, we can only speculate as to why the T229/A230 variant performs much better than the others (**Page 21** of the manuscript). Possible explanations that we propose are the location of the T229/A230 residues at the inner domain of the actin molecule while most actin-binding proteins associate to the outer domain, and the higher structural plasticity of this region as compared to the rest of the molecule, as found during an alanine mutagenesis screen (Rommelaere et al., Structure, 2003). To note, we have now also included AlphaFold predictions of the tagged actins in this response letter (**Page 8-9**), which strongly suggest that the tag is not obstructing monomer-monomer binding or actin-tropomyosin binding.

- It is now well established that actin plays active and important roles in the nucleus : is ALFA-actin correctly translocated to the nucleus ?

Revision: This is an interesting suggestion by the Reviewer and we have now performed nucleus-cytosol fractionation experiments and determined if ALFA-β-actin is still correctly translocated to the nucleus. The results of these experiments are included as **Fig. S6B** in the revised manuscript (text on **Page 8**) and from the results, we conclude that tagged actin localizes to the nucleus in similar amounts as wildtype actin.

- OPTIONAL: one may regret that there is no classical in vitro assays, such as pyrene assays to assess some kinetcis parameters on epitope-tagged actins. I guess this would make the paper a bit too large. Although, it will prove useful to better understand how much formin activity is affected (see below)

For further biochemical characterization and a detailed investigation of the precise assembly kinetics of the tagged actins, we (KD, SP) are already working together to set up *in vitro* reconstitution experiments. Yet, as also indicated by the Reviewer, we consider these experiments outside of the scope of the current work.

Minor comments:
Below are points that could be addressed by the authors to improve the manuscript readability and highlight some important points that are sometimes missing or are not properly discussed:

-line 40 "...but the distinct N-terminal epitope is not available under native conditions preventing" is a bit too obscure. Can the authors say clearly what is meant by 'native conditions'?
In our understanding, the term 'native' is generally used when referring to conditions in which proteins are in their natural state, without alterations due to heat or denaturants, and possibly also still interacting with their binding partners. Nonetheless, we have now rephrased this sentence (**Page 2** of the revised manuscript) to be more specific. Also on **Page 11**, we have now replaced the word "native" by "whole cell lysates" to further avoid confusion.

- figure 1A : make a clearer correspondance between the number shown in panel A and the amino acid numbers displayed in panel C and G.
Revision: We have now added extra annotation in the graph in **Fig. 1C and G** and **Fig. S3B** to better link the panels with each other. We have also added additional annotation in **Fig. 1D-F** and **Fig. S3B** for the same purpose.

- figure 1A : it could be informative to indicate subdomains in this panel.
Revision: We have now added the numbers for the subdomains in **Fig. 1A**.

- figure 1C : normalized correlation cell : I am not sure I understand how the normalization of the Pearson coefficient is done. It is therefore not clear how can it >1 or >-1 ? This should be clearly explained in the method section of the paper.
We normalized the Pearson coefficient values for visual representation of the results. The majority of the raw coefficient values (more than 80%) are between 0.20 and 0.75 (see raw values in the associated excel file). Theoretically, Pearson coefficient values are possible between 1 (or-1 for negative correlations) and 0. The much smaller window in our values as compared to the theoretical window (0.55 vs 1) led us to normalize the values such that they can be presented on a scale from "maximum expected colocalization" to "minimum expected colocalization". In this way, the differences between the various tagged actins are much better appreciated in the Figure. As also noted by the Reviewer, an important consequence of this normalization procedure is that values can become larger than 1, i.e. if the raw pearson correlation coefficient of a specific variant is larger than mean value of the N-terminal control. Importantly, in the Source Data file that will be accompanied with the manuscript, we do list the raw R-values for transparency.

Revision: We have now better explained the normalization procedure in the Methods section of the revised manuscript, including an equation that describes how we normalized the pearson coefficient values (**Page 35**).

- figure S4 : comes a bit too early when ALFA-actin has not been yet introduced in the main text. Please, reposition this part or provide data with the FLAG-tag version.
Revision: This is a good point and completely overlooked by us. We have now placed this Figure much later in the manuscript (**Fig. S13**), in the context of **Fig. 5** where the other actin variants are presented, such that the ALFA tag is already introduced.

- section starting line 121 : this section should be better motivated = Why are different tags being tested ? This comes later in the discussion, but the reader fails at following the reasoning/motivation here.

Revision: We have now rephrased the sentences at the start of this section (**Page 6-7** of the revised manuscript) to better motivate for why we added multiple tags.

- figure 2D, line 145 "We also evaluated actin protein expression in the homozygous ALFA-β-actin cells and this showed that the total amount of β-actin was slightly lower in the ALFA-β-actin cells compared to parental HT1080 cells (Fig. 2C-D)." 'Slightly' is not a very quantitative nor accurate term. please rephrase. Besides, a statistical test for the paired data would also be informative. Besides, data in figure S6B-D indeed show a correlated increase in the expression of Gamma-actin that compensate for the decrease in the Beta-actin level in ALFA-Beta-actin. Can the authors explain why they conclude otherwise?

Revision: We agree with the Reviewer that the word "slightly" is not very accurate and we have now changed the phrasing of this section (**Page 7-8** of the revised manuscript) to provide a more quantitative description of the western blot quantifications. As to the expression of γ-actin in the homozygous ALFA-β-actin cells, we only observed an approximately 8 percent increase in the band density on a western blot when normalized to the loading control. In combination with our observations that the expression of γ-actin is unchanged in the FLAG-β-actin (now added as **Fig. S5B-C** in the revised manuscript), this led us to conclude that overall regulation of actin isoform expression is not affected. Nonetheless, also here, we have now been more specific in the text and added the fact that an approximately 8 percent increase in γ-actin protein is observed in the ALFA-β-actin cells (**Page 8**).

- figure S7B: I am not ure anyone has ever reported measurement of angle of branched actin filament using epifluorescence microscopy. I would remove this panel, or the authors should explain how this measurement can be done objectively.

We apologize for this misapprehension from our side which is also noted by the other two reviewers. In the treadmilling videos of the lamellipodia in HT1080 cells, which were obtained using Airyscan super-resolution microscopy, we clearly observe a consistent filament formation at a constant angle, something which we interpreted as the angle between the mother filament and the daughter filament. After consulting the literature, we indeed have to admit that this cannot be interpreted as such because of a lack of resolution and we will remove these datasets.

- Figure 2F : can the authors comment on the (significant ?) lower value for FLAG-tag actin ?

The lower value for FLAG-tag actin has likely to do with the properties of the antibody. For reasons that we do not know, we usually detect more background for the FLAG tag antibody as compared to the other antibodies/ALFA tag nanobody. Since the Pearson correlation coefficient is very sensitive for these type of intensity differences, this is likely the reason that the values for FLAG-actin are lower as compared to the other tagged actins. Importantly, in our biochemistry experiments (F/G-actin), we detect no difference between FLAG-actin and ALFA-actin, and we therefore concluded that it is rather the immunofluorescence and sensitive Pearson correlation analysis than a differential integration of actin that causes this difference.

- line 205 "The results from these experiments show that both DIAPH1 and FMNL2 associate with ALFA-β-actin (Fig. 3D),". It is not so obvious that these formins directly interact with monomeric actin via their FH2 domains in co-immunoprecipitation assays. It might very well be mediated by the interaction with profilin, that in turn bind to the FH1 domain of formins. For me, this assay does not make a correct proof that epitope-labelled actin do not interfere with formin activity.

Revision: The point that the co-immunoprecipitation does not demonstrate direct interactions between formins and actin is well taken. We, however, do not claim that this assay proofs that formin activity, or formin-based integration of actin monomers, is similar with tagged actin as compared to wildtype actin. We have deliberately used "associate with" in the context of formin-binding to avoid the suggestion that we are only investigating direct interactions. In the revised manuscript (**Page 11**), we have now rephrased "bind to" to "associate with" consistently throughout this section and added "*either directly or indirectly through profilin*" to further emphasize that the co-immunoprecipitation cannot distinguish between direct and indirect interactions.

- figure 5C&D : both graph should use the same scale for the y-axis for easier comparison.
Revision: We have completely revised **Fig. 5**, including consistent scales on the y-axes, and hope that it now better demonstrates the observed differences between the localization of β- and γ-actin in parental and IntAct cells.

- figure 5D: I think the way the ratio is performed is misleading. Why not look at the Beta/Gamma ratio using the isoform specific antibodies used in parental cells, and show the results for ALFA-Beta-actin and for ALFA-Gamma-actin separately ?
Revision: We kindly refer to our answer to ***Reviewer 1*** on Page 2 of this response letter for a detailed explanation on the experimental challenge of comparing the localization of wildtype and tagged actin isoforms. Although we have considered to use the strategy proposed by the Reviewer, we deliberately avoided this because, besides determining the differential localization of the isoactins, we aimed to emphasize the strong potential of our ALFA-β-actin and ALFA-γ-actin cells for immunofluorescence applications with the use of the ALFA nanobodies and without using methanol as a fixation agent. Avoiding the use of methanol opens up many applications of costaining the actin isoforms with potential binding partners and study their spatial relationschip. Importantly, based on the suggestions of the Reviewer (and ***Reviewer 1***) we have now thoroughly revised the original Figure 5 and we hope the Reviewer agrees that we now much more convincingly demonstrate that the tagged actin isoforms localize similarly to their WT counterparts. Also, to avoid any confusion as to how the ration calculations have been performed, we have now better explained these in the Methods section of the revised manuscript (**Page 35-36**).

- The limitation observed for unbranched cables in yeast that nanobody-tagged ALFA-actin does not incorporate correctly should be discussed and stressed further in the discussion, as it might prove to be a strong limitation for live-cell imaging to reliably study any type of actin networks.
We acknowledge the limitation that the Reviewer points out regarding the inability of ALFA-tagged actin to incorporate into yeast actin cables when ALFA-Nb-mNG is co-expressed, something which we have now also observed for fission yeast (**Fig. 5E and Fig. S23**) and in budding yeast with low expression levels of ALFA-Nb-mNG (**Fig. S22**). To emphasize this limitation, we had already pointed this out in the discussion of our original manuscript, mentioning possible explanations for these observations as well as stressing the importance of the development of new tag/probe combinations for live cell imaging. Based on the Reviewer's suggestion, we have now further extended and rephrased this part of the discussion and explicitly mentioned that our inability to visualize actin cables and rings in living yeast cells represents a limitation of our IntAct system (**Page 23** of the revised manuscript). We also propose options for future improvements such as the expression of ALFA-Nb variants with high dissociation rates (Zhang et al., Cell Rep Meth, 2022).

Reviewer #3 (Significance (Required)):
General assessment:
This paper provides a new tagging strategy to monitor actin activity in cells, by specifically inserting the tag along the amino acid sequence.

Advance:
This is a very useful tool, as most existing available probes bind to actin in regions that are common to many other actin binding proteins. The authors provide extensive experiments to validate that tagged-actin are functional and do not perturb the actin expression level, actin network architecture nor dynamics.
Audience:
This research paper will be of interest to a rather broad audience (many cell biologists) that are either sutyding actin dynamics or know that actin is involved in the cell functions they study.
Expertise:
My expertise is in vitro actin biochemistry.
